# No Influence of Sleep Quality and Chronotype on the Effects of Mental Fatigue: Evidence From an Exploratory Analysis

**DOI:** 10.1002/ejsc.70247

**Published:** 2026-09-10

**Authors:** Jelle Habay, Quinten Ezzy, Y. Laurisa Arenales Arauz, Matthias Proost, Suzanna Russell, Kevin De Pauw, Jeroen Van Cutsem, Bart Roelands, Nathalie Pattyn

**Affiliations:** ^1^ Faculty of Physical Education and Physiotherapy Human Physiology and Sports Physiotherapy Research Group Vrije Universiteit Brussel Brussels Belgium; ^2^ LIFE Department Vital Signs and Performance Monitoring Research Unit Royal Military Academy Brussels Belgium; ^3^ Research Foundation Flanders (FWO) Brussels Belgium; ^4^ BruBotics Vrije Universiteit Brussel Brussels Belgium; ^5^ Laboratory of Sports and Nutrition Research Riga Stradins University Riga Latvia; ^6^ Sleep Health and Human Performance Lab Centre d’Etudes Avancées en Médecine du Sommeil CIUSSS NÏM & Université de Montréal Montréal Quebec Canada

**Keywords:** caffeine consumption, circadian influence, interindividual variability, moderation analyses, PSQI

## Abstract

A recent trial showed a negative impact of mental fatigue (MF) on subjective and behavioural outcomes. However, effects on physical performance outcomes were smaller than previously reported, possibly due to interindividual differences. To explore these differences, we examined whether sleep quality and chronotype modulate subjective and behavioural responses to MF. Mental fatigue was induced using a 45 minute Stroop task, while the control condition consisted of watching a documentary of equal duration. Experiments were conducted at approximately the same time of day across participants. Dependent variables included the level of subjective MF (visual analogue scale), cognitive performance (GoNoGo task), physical performance (time trial) and the Rating of Perceived Exertion (during the time trial). Sleep quality (Pittsburgh Sleep Quality Index) and chronotype (Single Item Chronotype scale) were added as between subject variables in exploratory moderation analyses. Weekly caffeine consumption was included as a covariate in the analyses. Most analyses revealed no meaningful differences in MF response between chronotype or sleep‐quality groups. Any differences that were found were focused within the subjective variables, with a significant influence of sleep quality (*p* = 0.028) and chronotype (*p* = 0.002) on the general subjective feeling of MF and a significant difference between chronotype groups for the feeling of sleepiness (*p* < 0.001). Future research should incorporate multidimensional subjective and objective measures of sleep and include athlete specific populations to further clarify the distinction between MF and impaired sleep quality.

**Trial Registration:**

The study was registered on ClinicalTrials.gov (NCT05576935)

## Highlights

- Subjective and behavioural mental fatigue responses showed minimal differences between chronotype and sleep quality groups.
- Due to the exploratory nature of the study, further research with error‐controlled analyses and more appropriate methodology is necessary to confirm these limited differences in mental fatigue response between sleep quality and chronotype groups.
- Clear differentiation between mental fatigue and sleep behaviour is essential in both research and practice, given the unresolved complexities of each.

## 1 Introduction

Mental fatigue (MF) is defined as a psychobiological state caused by prolonged periods of demanding cognitive activity (Van Cutsem et al. 2017). It results in an acute feeling of tiredness (Smith et al. 2019), altered brain activity (Tran et al. 2020) and impairments in both cognitive and physical performance (Habay, Van Cutsem, et al. 2021). Our recent large‐scale randomized crossover investigation provided further context to this complex phenomenon (Habay et al. 2025). The data set of this study included over 115 participants, with findings confirming the detrimental influence of MF on subjective and behavioural outcomes (D. M. Y. Y. Brown et al. 2020; Habay et al. 2025; Van Cutsem et al. 2017). However, negative influences on physical performance and rating of perceived exertion (RPE) remained limited (Habay et al. 2025), with smaller effect sizes compared to previous literature (D. M. Y. Y. Brown et al. 2020; Marcora et al. 2009; Van Cutsem et al. 2017). These limited effects were associated with a considerable degree of interindividual variability (Habay et al. 2025), building upon previous findings of systematic analyses (Habay et al. 2023). To explore the origin of this variability, we suggested conducting secondary analyses on the data set while accounting for a variety of individual features (e.g., age, sex, training level) (Habay et al. 2025).

One particular set of features that could be of interest are sleep quality and chronotype. For one, the negative effects of acute sleep restriction on cognitive and physical performance (Craven et al. 2022; Goel et al. 2009) are similar to those observed following acute MF inducement (Oliver et al. 2025; Van Cutsem et al. 2017). Authors have also proposed that the physiological mechanisms of both phenomena share similar theoretical foundations (R. E. Brown et al. 2012; Martin et al. 2018). These similarities are further highlighted when research looks at both phenomena simultaneously. Pastier et al. (2022) showed an increase in self‐reported MF that was linked to poorer self‐reported sleep quality. Similarly, Descotes et al. (2025) discovered that subjective sleep quality was negatively correlated with the subjective level of MF in ice hockey players. Some studies have also combined sleep restriction with experimental MF protocols (Barbosa et al. 2025; Filipas et al. 2021). Results show little additional impairment on sport‐specific physical performance, further accentuating their overlapping mechanisms which lead to a ceiling effect (Barbosa et al. 2025; Filipas et al. 2021). Meanwhile, circadian rhythm disruption due to shift work in surgeons has been linked with an accumulation of MF in this professional group (Laulan et al. 2025). As this disruption is also possible in athletes (Janse Van Rensburg et al. 2021), their level of MF could also change in unknown ways. Additional research into the link between sleep and circadian‐related variables and MF could provide indications on overlapping mechanisms, while informing practitioners on the importance of quantifying both phenomena to guide individualized management and optimize human performance.

We chose to specifically investigate the difference in MF response between two sleep‐related variables: sleep quality, measured using the Pittsburgh Sleep Quality Index (PSQI) (Buysse et al. 1989) and chronotype, measured using the single item chronotype scale (SIC) (Putilov, Sveshnikov, et al. 2021). The PSQI was selected as a measure of sleep behaviour because of its widespread use in research and clinical settings, its ease of administration and interpretation and its ability to capture a variety of sleep‐related information (Mollayeva et al. 2016). Diurnal variation in physical performance has been a focus of sleep and exercise science research for the last few decades (Forster et al. 2025). Recent evidence suggests that chronotype (i.e., an individual's predisposition towards morning or evening (Vitale and Weydahl 2017)) influences mental readiness and physical performance in individuals depending on the time of day (Forster et al. 2025). Chronotype was therefore included as a potentially relevant explanatory factor. In addition, weekly caffeine consumption was also considered in this study, using the revised caffeine consumption questionnaire (CCQ‐R) (Irons et al. 2016). As caffeine acts as an adenosine receptor antagonist (Cappelletti et al. 2015) and has been shown to influence both MF responses as well as the effects of sleep restriction (Clark and Landolt 2017; Proost et al. 2022), it was considered a relevant covariate in the present analyses.

It is still difficult to predict how these collected sleep related variables will influence MF responses due to the limited research available. We therefore opted to perform a non‐error controlled exploratory analysis, conform the guidelines proposed by Ditroilo et al. (2025). As such, our hypotheses were not preregistered in any way. The aim of this exploratory study was to investigate differences in MF response based on the collected variables related to sleep, namely sleep quality and chronotype, while adding weekly caffeine consumption as a covariate.

## 2 Materials and Methods

This experiment was originally approved by the medical ethics commission of the Vrije Universiteit Brussel and the affiliated University Hospital (B.UN. 1432022000084), being part of a larger research project (FWO 11J6323N) registered on ClinicalTrials.gov (NCT05576935). A detailed overview of the protocol as well as the full collected dataset are published elsewhere (Habay et al. 2025). The present manuscript was designed based on the recent publication by Ditroilo et al. (2025) and in accordance with the Settings, Protocol establishments, Confounders, Individuals, Framework and Yield (SPeCIFY) reporting guidelines (Schampheleer et al. 2025).

### 2.1 Design

The present study employed a randomized, single‐blinded, controlled, counter‐balanced, cross‐over design, where participants were instructed to return on three separate occasions (familiarization, intervention and control trial). The intervention and control trials were always conducted in the morning, between 7 and 13h. Moreover, we aimed to keep the visiting time the same within the same participant, with a maximal difference in start time of 1 hour. The experimental trials (i.e., intervention and control) were separated by at least 3 days to ensure full recovery. All trials were performed at the Brussels Laboratorium voor Inspanning en TopSport (BLITS) in a secluded climate chamber with standardized conditions (20°C, 40% humidity). Treatment order was randomized by a web‐based computer program (www.randomization.org). Participants were blinded from treatment allocation as they were told that the purpose of the research was to investigate the differences in brain activation between different cognitive and physical tasks.

### 2.2 Participants

104 healthy participants (male = 56; female = 48; age = 33 ± 9 y; weight = 70.2 ± 11.7 kg; height = 175 ± 10 cm; BMI = 22.81 ± 2.61 kg/m^2^; fat percentage = 20.5 ± 7.8%; relative VO_2_Max = 48 ± 10 mL/kg/min) were included in the exploratory analysis of the present study. Participants were classified as healthy if they did not have any chronic health conditions, any neurological, cardiovascular or musculoskeletal disorders, any injuries in the past 6 months or a history of suffering from mental/psychiatric disorders. This last requirement was checked through an inclusion questionnaire featuring the multidimensional fatigue inventory (Smets et al. 1995), the Burn Out Assessment tool (Schaufeli et al. 2020) and the Beck Depression Inventory‐II (Wang and Gorenstein 2013). Explanation on questionnaire use and cut of scores used can be found in Digital Supporting Information S1: SF1 the original experiment (Habay et al. 2025). Applicants were excluded from participation if they used any kind of medication or smoked and were instructed not to perform any strenuous exercise or consume any caffeinated products the day before and morning of each trial. Participants were required to sleep at least 7 hours and to consume a similar meal the evening and morning before each trial. Prior to participation in the investigation all participants signed an informed consent document.

### 2.3 Study Procedures

Upon arrival at the laboratory for the familiarization trial, participants were given a standard explanation and overview of the investigation. Afterwards, the health of participants was checked during a routine medical examination and participants were required to perform a maximal incremental exercise test. Immediately after this, participants were subjected to a screening to determine a personalized profile, featuring subjective questionnaires and behavioural tests. Among these questionnaires were the PSQI (Buysse et al. 1989) to determine sleep quality and quantity, the SIC (Putilov, Sveshnikov, et al. 2021) to determine chronotype and the CCQ‐R (Irons et al. 2016) to determine self‐reported weekly caffeine consumption. Hereafter, participants were familiarized with the study procedures of the coming test days. Importantly, the intervention task was familiarized in the form of a ‘Stroop max test’ in order to individualize the cognitive load to be administered for each participant during the intervention trial (for a detailed explanation on the Stroop Max task, see Habay et al. (2025)). Briefly, in the Stroop task, four colour names (i.e., ‘rood’, ‘blauw’, ‘geel’ and ‘groen’) were presented on screen in coloured (i.e., red, blue, yellow and green) letters. Participants were required to react to the colour of the word, except if the colour was red. Then, participants were instructed to respond with the meaning of the word with the corresponding colour buttons on a keyboard. The Stroop task employed in the present series of experiments was based on previous references aimed at taxing participants' inhibitory control (Schampheleer et al. 2025). The Stroop max task features 72 stimuli with a varying interstimulus time between 1100 and 1900 ms. To individualize the cognitive load during the intervention condition, the stimulus presentation time is progressively decreased in 100 ms intervals (starting at 1100 ms) until participants are unable to keep up an accuracy of greater or equal to 85%. Participants were allowed three attempts at each level and only five additional attempts in total. The level chosen for the eventual intervention task was the level that was last successfully reached by the participants.

Before each experimental trial (i.e., either intervention with MF or control), participants filled in a pre‐test checklist to determine whether they adhered to the pre‐trial criteria. Hereafter, they performed the first cognitive performance task (i.e., the GoNoGo task) and some baseline questionnaires (i.e., the MF visual analogue scale (M‐VAS) and the Karolinska sleepiness scale (KSS)) to establish a baseline. Then participants started with either the intervention (Stroop task) or control (documentary) condition. The utilised Stroop task consisted of three blocks of 360 stimuli, with a varying interstimulus time between 1100 and 1900 ms and a stimulus presentation time based on the performance of the Stroop max task (which led to a task duration of about 45 minutes depending on the level of the Stroop max task) (Habay, Proost, et al. 2021). In the control condition, participants watched an emotionally neutral documentary featured on the streaming site Disney + which they chose from a standard list provided during the familiarisation trial (Habay et al. 2025). After the Stroop task, participants were again asked to provide their subjective level of MF and sleepiness, before conducting the post cognitive performance (i.e., GoNoGo) task. The trial ended with a physical performance task (i.e., a cycling time trial), where the RPE was collected every 3 minutes.

### 2.4 Outcomes

#### 2.4.1 Subjective Measures of Mental Fatigue Response

The subjective level of MF was quantified using a 100mm M‐VAS with the accompanying question: ‘*How mentally fatigued do you feel?*’; with the response ranging from ‘*not at all*’ to ‘*completely exhausted*’ (Lee et al. 1991). The M‐VAS was measured before and after the intervention/control task. Meanwhile, the RPE of participants was collected every three minutes during the completion of the 20 minute time trial using the CR−100 scale with the accompanying question: ‘how heavy do you perceive the task to be on a scale of 0–100?’ (Borg and Kaijser 2006). Finally, the KSS (Kaida et al. 2006) was conducted before and after the intervention/control task to assess acute sleepiness response related to MF. This 10‐point scale measures the subjective level of sleepiness and goes from 1 = ‘extremely alert’ to 10 = ‘extremely sleepy, falls asleep all the time’.

#### 2.4.2 Behavioural Measures of Mental Fatigue Response

To determine the cognitive response to MF, a modified Go/NoGo task was employed (Arenales Arauz et al. 2024). During the task, two objects were presented on the screen: a geometrical figure (i.e., square or triangle) and an arrow (i.e., pointing either left or right). When a square was presented (i.e., ‘Go’ stimulus), participants were asked to react as fast and accurately as possible to the arrow with the appropriate keys on the keyboard. When a triangle was presented (i.e., ‘NoGo’ stimulus) participants were instructed to withhold any response and to wait until a new stimulus appeared. The Go/NoGo ratio was set at 80/20, with 3 blocks of 60 stimuli (with 48 ‘Go’ and 12 ‘NoGo’ stimuli within each block). These stimuli were presented on screen 500 ms, with a varying interstimulus interval ranging between 1100 and 1700 ms. A mean *Z* score of the GoNoGo task was calculated based on the inverse *Z* score of the Go reaction time and the *Z* score of the Go accuracy as a way to provide a general measure of the cognitive response to MF.

At the end of each intervention/control trial participants performed a 20 minute cycling time‐trial on a cycling ergometer (Cyclus 2; version 4.2, Leipzig, Germany). The time‐trial consisted of a 3 minute warm up, a 15 minute self‐paced time‐trial and a 2 minute cool down. Resistance was identical during the warm up (80 W) and cool down (60 W) periods. Participants were free to choose their resistance during the 15 minute time trial itself and were instructed to perform as much distance as possible within the limited time frame. Continuous cooling was provided using a fan (Wahoo KICKR Headwind Ventilator). Physical performance was measured primarily as the covered distance in the 15 minute trial. Participants were blinded from all information regarding their performance except for the remaining time of the protocol and no motivational instructions were given at any point.

#### 2.4.3 Sleep Quality Measure

Sleep quality was questioned using the PSQI (Buysse et al. 1989). The PSQI assesses 19 self‐reported items divided in seven subcategories, each equally weighted on a 0−3 scale, for determining sleep quality: 1. Subjective sleep quality, 2. Sleep latency, 3. Sleep duration, 4. Habitual sleep efficiency, 5. Sleep disturbances, 6. Use of sleeping medication and 7. Daytime dysfunction. An overall score of sleep quality is calculated based on the sum of all subcategories, with a maximal score of 21 (with a higher score representing a worse sleep quality). Moreover, sleep quantity (determined by participants' reported ‘sleep duration’ of the PSQI) was also included as an isolated variable in the descriptive analyses.

#### 2.4.4 Chronotype Measure

**FIGURE 1:**
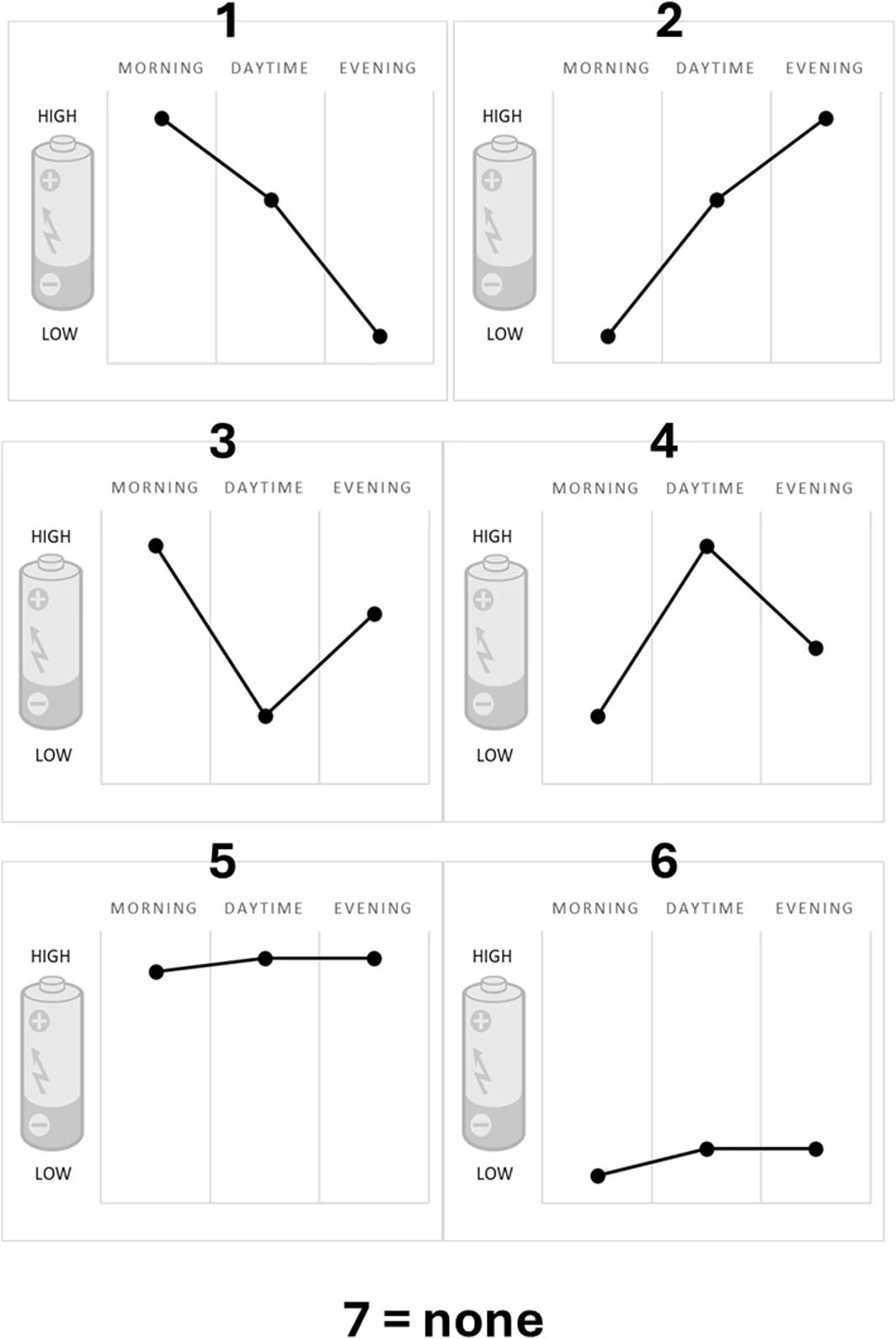
The single item chronotype scale as presented to the participants. Participants were instructed to indicate the chronotype that best represented the fluctuation in their overall activity levels. This scale was adapted from the original publication of Putilov, Budkevich, et al. (2021). 1. Morning type, 2. evening type, 3. daytime sleepy type, 4. daytime type, 5. highly active type, 6. moderately active type.

The SIC was used to determine the chronotype of participants. It assesses one's alertness level and changes in alertness level during the day by providing participants with different simple figures of these changes throughout the day (Putilov, Sveshnikov, et al. 2021). The SIC was developed using principal component analysis and provides an easy and understandable alternative compared to other chronotype scales (Putilov, Sveshnikov, et al. 2021). It has been subsequently validated against multiple other relevant concepts, such as chronotype, quantified through the visuo‐verbal judgement task and the sleep‐wake adaptability test; sleepiness throughout the day, quantified using the Epworth sleepiness scale; and self‐reported times for sleep onset and offset, quantified using the Munich ChronoType Questionnaire (Putilov, Budkevich, et al. 2021; Putilov, Sveshnikov, et al. 2021). Participants were required to choose one of the six options that represent activation levels over the morning, daytime and evening in view of identifying their chronotype: 1. Morning type, 2. Evening type, 3. Daytime sleepy type, 4. Daytime type, 5. Highly active type, 6. Moderately active type (see also Figure 1). Additionally, a seventh option identified as ‘*none*’ was provided if neither of the six chronotype options corresponded with their activation levels during the day.

#### 2.4.5 Measure of Caffeine Consumption

The CCQ‐R (Irons et al. 2016) was used to measure participants' average weekly caffeine consumption by questioning the consumed amount and serving sizes of foods, fluids and over‐the‐counter drugs containing caffeine. Subsequently, the total number of reported servings and amount of units were converted in item‐type subscores and were summed to determine the total amount in milligram.

### 2.5 Statistical Analysis

All statistical analyses were conducted in R (version 4.5.1) through R studio (version 2025.05.1). The performed analyses can be found in Supporting Information S2: SF2, while the utilised data file with additional data not included in the original article (10.1249/MSS.0000000000003852; (Habay et al. 2025)) can be found in Supporting Information S1: SF1. To visualize the difference in effect between the groups, delta's were constructed by subtracting control values from intervention values. Most of the figures that include these delta values, along with additional descriptive information on the PSQI and Caffeine consumption values can be found in Supporting Information S3: SF3. All data are presented using mean ± standard deviation. The significance value was set at 0.05 for all analyses. Results were defined as trends to significance when *p* values were situated between 0.05 and 0.1. Normality was checked using the Shapiro wilk test. If data was not normally distributed (i.e., half of the investigated variables were not normally distributed), data was first transformed using square root transformations. 95% CIs are mentioned whenever possible. Partial eta squared values were classified as follows: < 0.02 = trivial; 0.02–0.13 = small; 0.13–0.26 = moderate; > 0.26 = large. Odds ratio size was interpreted following Chen et al. (2010).

The differences in MF response between chronotype and sleep quality groups was assessed by adding the groups as a between factor in the models of the original study. Chronotype groups were established using the SIC (see Chronotype measure; seven distinct groups based on the answers to the questionnaire: Morning type, Evening type, Daytime sleepy type, Daytime type, Highly active type, Moderately active type and None). Sleep quality groups were made based on the distribution of sleep quality scores. This division was performed exploratory, where effort was made to divide participants in distinct groups with considerable differences in total sleep quality score with an equal number of participants in every group. In the end, M‐VAS, Go stimuli *Z* score and RPE values were investigated using linear mixed models (fixed effect: condition, time and sleep related variable; either chronotype or sleep quality; random effect: participant number). KSS values were analysed using ordinal regression (fixed effect: condition, time and sleep related variable; either chronotype or sleep quality). To address concerns regarding the use of post‐hoc cut‐offs, additional sensitivity analyses were conducted using PSQI as a continuous variable in the full sample. These models mirrored the original analyses by including condition, time and their interactions with PSQI.

Caffeine consumption was added in every model as a covariate. If the value did not influence the overall model, it was removed. Other features of the original population (i.e., sex, age, BMI, fat percentage and relative VO_2_max) were compared between the different chronotype and sleep quality groups using one way Anova's. In the event of a significant difference between groups for a specific feature, this feature was also added in the proposed models as a covariate.

Large language models (i.e., Chat‐GPT and Copilot) were used as guidance to correct grammar and writing style throughout the manuscript.

## 3 Results

### 3.1 Descriptives and Preliminary Analyses

**FIGURE 2:**
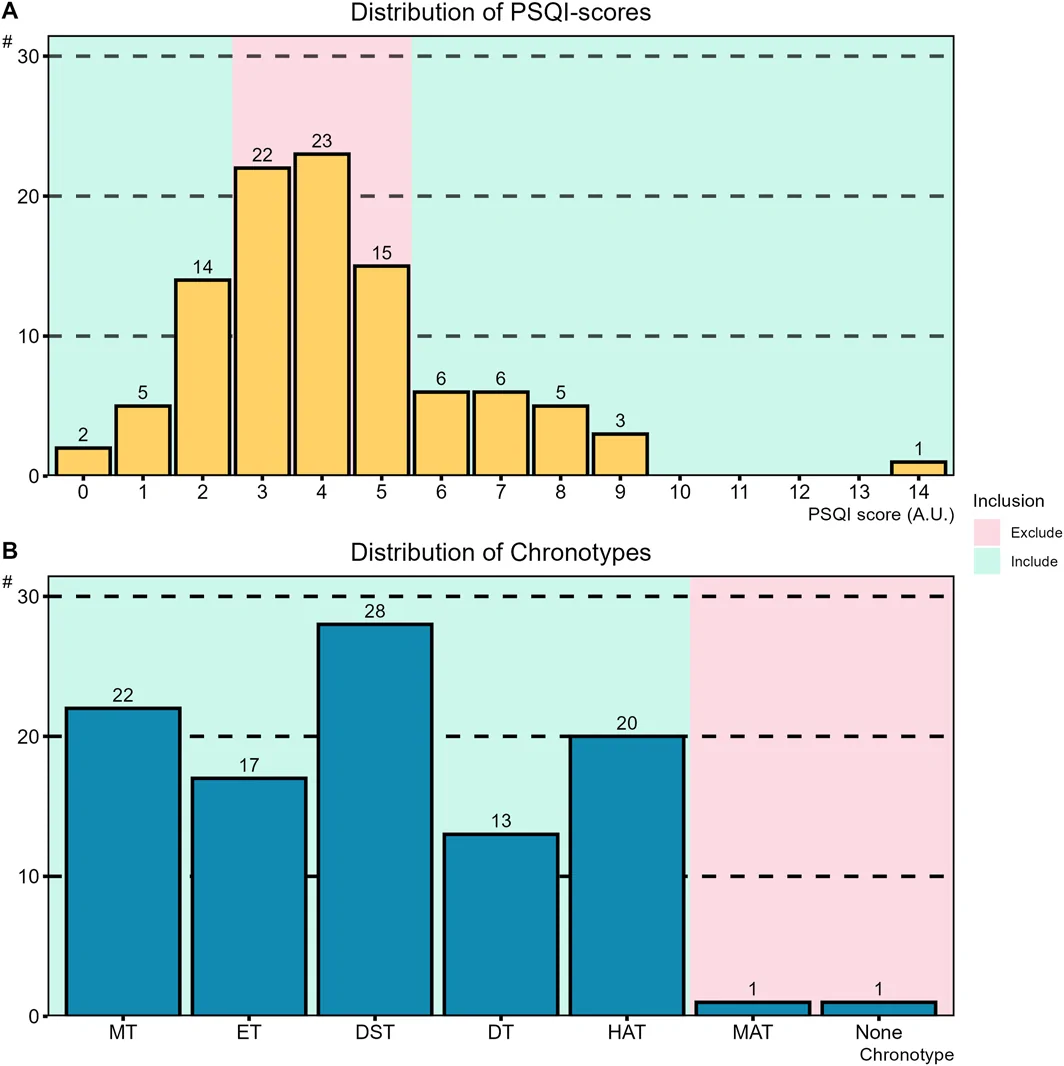
Overview of the number of participants for every chronotype. Yellow bars show the distribution of PSQI values, while the blue bars show the distribution of chronotype values. The green (include) and red (exclude) background shows which data was used in our final analyses. (# = number of participants; A.U. = arbitrary units; DST = daytime sleepy type; DT = daytime type; ET = evening type; HAT = highly active type; MAT = ‘moderately active type’; MT = morning type).

An overview of the participant distribution for every chronotype and sleep quality score is presented in Figure 2, with the green colour. Based on the distribution, the choice was made to exclude chronotype 6 and 7 from further analyses, as these groups were represented by 0 and 1 person respectively. To achieve an equal distribution between sleep quality groups, cut offs were made at a PSQI value of lower or equal to 2 (‘good’ sleep quality; 21 participants) and higher or equal to 6 (‘bad’ sleep quality; 21 participants).

Table 1 portrays the differences in individual features between chronotype and PSQI groups. As the age of participants was significantly different between the chronotype groups, this variable was included as a covariate in further analyses. The mean time between experimental sessions was 13 days, the median 7.

**TABLE 1:** Differences in individual features between chronotype and sleep quality groups.

Characteristic	Chronotype						PSQI groups		
	Type 1 (MT)	Type 2 (ET)	Type 3 (DST)	Type 4 (DT)	Type 5 (HAT)	*p*	Group 1 (‘good’ SQ)	Group 2 (‘bad’ SQ)	*p*
Sex (*n*)									
• Male	12	7	14	8	13	0.630	12	12	1.00
• Female	10	10	14	5	7		9	9	
Age (y)	37 ± 8	31 ± 7	32 ± 9	31 ± 10	31 ± 8	**0.045** ^a^	34 ± 9	30 ± 6	0.103
BMI (kg/m^2^)	22.60 ± 2.17	23.98 ± 2.54	22.86 ± 2.55	21.77 ± 3.33	22.86 ± 2.60	0.560	23.55 ± 2.67	23.16 ± 2.60	0.634
Fat percentage (%)	20.1 ± 6.4	24.7 ± 8.4	21.4 ± 8.3	18.4 ± 8.7	18.1 ± 6.4	0.122	21.8 ± 8.5	20.0 ± 7.7	0.554
rVO_2_Max (mL/min/kg)	47 ± 8	43 ± 9	50 ± 10	46 ± 11	51 ± 9	*0.06* ^b^	47 ± 11	49 ± 9	0.610
CCQ (mg/week)	1845 ± 1546	1610 ± 1668	1602 ± 1489	895 ± 853	1121 ± 1143	0.300	1831 ± 1736	1356 ± 1454	0.302
Sleep duration (hrs/night)	7.4 ± 0.6	7.2 ± 0.9	7.4 ± 0.8	7.6 ± 0.7	7.7 ± 0.9	0.134	7.9 ± 0.7	6.7 ± 0.9	**<** 0.001* ^a^

Abbreviations: DST = daytime sleepy type; DT = daytime type; ET = evening type; HAT = highly active type; MAT = ‘moderately active type’; MT = morning type; SQ = sleep quality.

^a^ Significant difference in bold.

^b^ Trend to significance in italics.

Preliminary analyses showed no difference in sleep quality (*F* = 1.27; *p* = 0.287; *η*_*p*_^2^ = 0.05), sleep duration (*F* = 228; *p* = 0.134; *η*_*p*_^2^ = 0.02) and caffeine consumption (*F* = 1.34; *p* = 0.259; *η*_*p*_^2^ = 0.05) between the different chronotypes. Similarly, there were no differences in caffeine consumption (*w* = 262; *p* = 0.302) and chronotype distribution (*χ* ^2^ = 7.36; *p* = 0.118) between sleep quality groups.

### 3.2 Influence of Chronotype on Mental Fatigue Responses

Table 2 provides an overview of the additional influence of chronotype on the original models of the selected outcome variables.

**TABLE 2:** Overview of the differences in mental fatigue variables between chronotype groups.

Outcome	Type	Additional effect of chronotype						Influence of covariate?
		Estimate	95% CI	Test statistic	*p*	ES	Int.	
Subjective feeling of mental fatigue	General	ET: < 0.01 DST: 0.61 DT: 0.25 HAT: −1.08	[−1.02; 1.03] [−0.30; 1.51] [−0.86; 1.36] [−2.06; −0.10]	4.57 (F)	0.002* ^a^	0.16 (*η*_*p*_^2^)	Moderate	No for caffeine (*p* = 0.597) and age (*p* = 0.688)
	Interaction with condition	ET: −0.26 DST: −0.56 DT: −0.12 HAT: 0.17	[−1.48; 0.96] [−1.64; 0.51] [−1.44; 1.02] [−1.00; 1.34]	2.72 (F)	**0.030** ^a^	0.04 (*η*_*p*_^2^)	Small	
	Interaction with time	ET: −0.68 DST: 0.90 DT: −0.07 HAT: 0.36	[−1.91; 0.54] [−0.17; 1.98] [−1.40; 1.25] [−0.81; 1.53]	1.49 (F)	0.206	0.02 (*η*_*p*_^2^)	Small	
	Interaction with condition*time	ET: 1.01 DST: −0.56 DT: −0.10 HAT: 0.30	[−0.72; 2.74] [−2.08; 0.97] [−1.97; 1.77] [−1.35; 1.95]	0.89 (F)	0.471	0.01 (*η*_*p*_^2^)	Trivial	
Rating of perceived exertion	General	/	/	1.02 (F)	0.401	0.04 (*η*_*p*_^2^)	Small	No for caffeine (*p* = 0.485) and age (*p* = 0.214)
	Interaction with condition	/	/	2.02 (F)	*0.089* ^b^	0.008 (*η*_*p*_^2^)	Trivial	
	Interaction with time	/	/	1.93 (F)	**0.009** ^a^	0.04 (*η*_*p*_^2^)	Small	
	Interaction with condition*time	/	/	0.36 (F)	0.996	0.007 (*η*_*p*_^2^)	Trivial	
Go stimuli *z* score	General	ET: 0.14 DST: 0.23 DT: 0.21 HAT: 0.24	[−0.27; 0.55] [−0.13; 0.59] [−0.23; 0.65] [−0.16; 0.63]	0.33 (F)	0.860	0.01 (*η*_*p*_^2^)	Trivial	No for caffeine (*p* = 0.342); yes for age (***p* = 0.005** ^a^; increase in age resulted in worse reaction time)
	Interaction with condition	ET: −0.22 DST: −0.06 DT: −0.34 HAT: 0.07	[−0.60; 0.16] [−0.40; 0.27] [−0.75; 0.07] [−0.29; 0.43]	1.67 (F)	0.157	0.02 (*η*_*p*_^2^)	Small	
	Interaction with time	ET: 0.06 DST: −0.05 DT: −0.06 HAT: −0.05	[−0.32; 0.44] [−0.38; 0.28] [−0.46; 0.35] [−0.41; 0.31]	1.42 (F)	0.228	0.02 (*η*_*p*_^2^)	Small	
	Interaction with condition*time	ET: −0.02 DST: −0.22 DT: −0.04 HAT: −0.36	[−0.55; 0.51] [−0.69; 0.25] [−0.62; 0.53] [−0.87; 0.15]	0.67 (F)	0.616	0.009 (*η*_*p*_^2^)	Trivial	
Time trial distance	General	ET: −0.53 DST: 0.90 DT: 0.12 HAT: 0.91	[−1.66; 0.61] [−0.06; 1.86] [−1.12; 1.37] [−0.12; 1.95]	2.39 (F)	*0.057* ^b^	0.10 (*η*_*p*_^2^)	Small	Yes (***p* = 0.030** ^a^; increase in caffeine use resulted in a higher overall time trial distance value); No for age (*p* = 0.289)
	Interaction with condition	ET: 0.04 DST: < 0.01 DT: 0.12 HAT: −0.09	[−0.58; 0.66] [−0.52; 0.52] [−0.55; 0.79] [−0.65; 0.47]	0.10 (F)	0.983	0.004 (*η*_*p*_^2^)	Trivial	
Karolinska sleepiness scale	General	ET: 1.13 DST: 0.63 DT: 0.43 HAT: −1.22	[−0.05; 2.32] [−0.39; 1.67] [−0.87; 1.73] [−2.36; −0.08]	30.99 (*χ* ^2^)	**<** **0.001** ^a^	0.30–3.10 (OR)	Trivial to medium	No for caffeine (*p* = 0.524) and age (*p = 0.058* ^b^)
	Interaction with condition	ET: −0.08 DST: −0.08 DT: 0.47 HAT: 0.77	[−1.71; 1.56] [−1.52; 1.36] [−1.30; 2.25] [−0.82; 2.35]	1.62 (*χ* ^2^)	0.805	0.93–2.16 (OR)	Trivial to small	
	Interaction with time	ET: 0.14 DST: 0.38 DT: 0.16 HAT: 0.90	[−1.52; 1.81] [−1.06; 1.83] [−1.59; 1.91] [−0.71; 2.36]	1.09 (*χ* ^2^)	0.896	1.16–2.45 (OR)	Trivial to medium	
	Interaction with conditionatime	ET: −0.39 DST: −0.53 DT: −0.79 HAT: −1.19	[−2.74; 1.96] [−2.55; 1.50] [−3.21; 1.63] [−3.41; 1.04]	1.20 (*χ* ^2^)	0.879	0.31–0.68 (OR)	Trivial	

*Note:* / too many data points to provide a good overview, estimates and 95% CIs can be found in the Supporting Information S1.

Abbreviations: CI = confidence interval; DST = daytime sleepy type; DT = daytime type; ES = effect size; ET = evening type; HAT = highly active type; Int. = interpretation; MT = morning type.

^a^ Significant effect in bold.

^b^ Trend to significance in italics.

**FIGURE 3:**
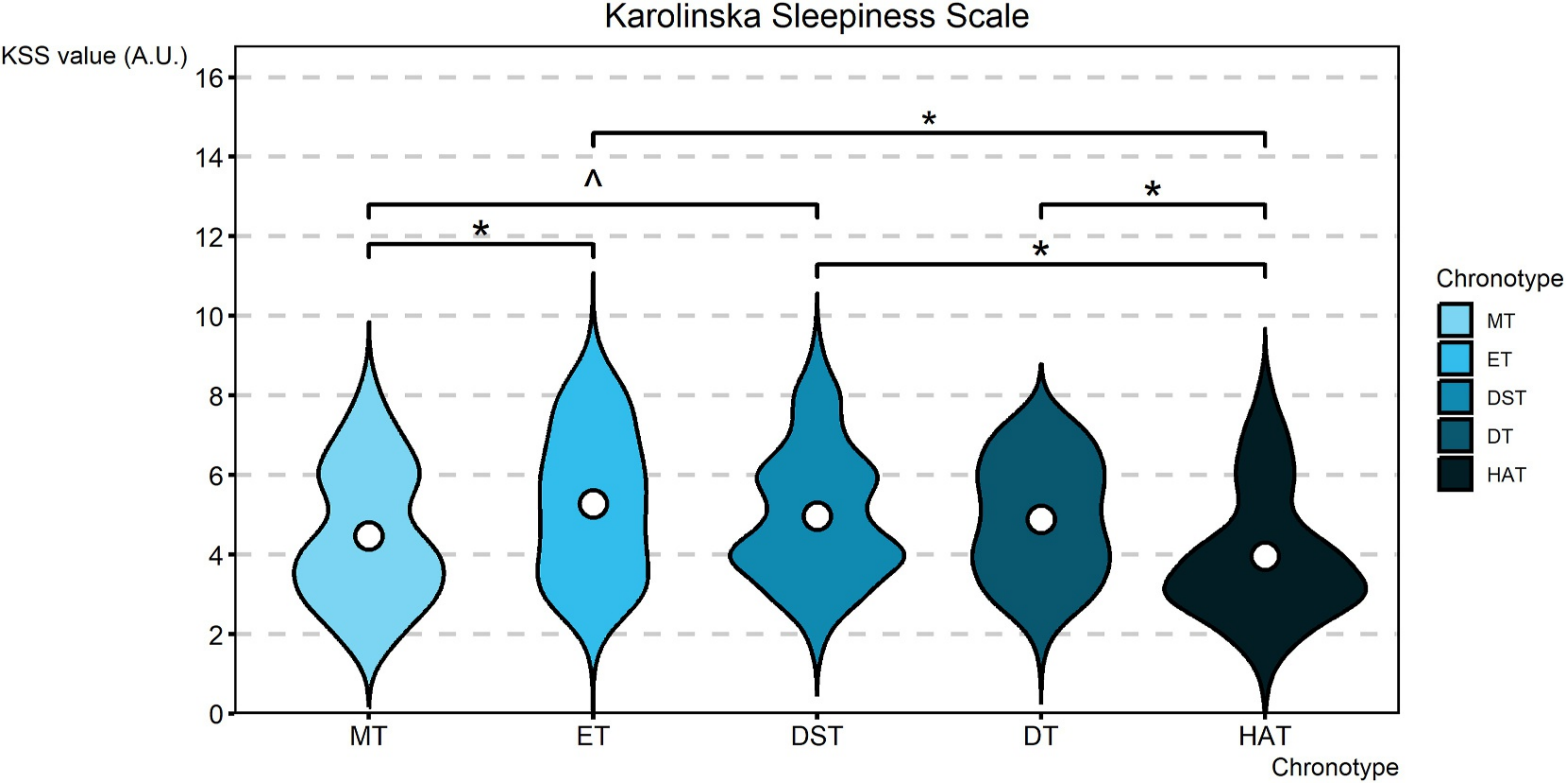
Violin plot to compare the mean differences in KSS values between the different chronotypes. The white points represent the mean value. * denotes a significant *p* value (< 0.05); ^ denotes a trend to significance (*p* < 0.1). (A.U. = arbitrary units; DST = daytime sleepy type; DT = daytime type; ET = evening type; HAT = highly active type; MT = morning type).

Results showed a significantly effect of chronotype on the general mean of the MVAS and KSS values. Post hoc pairwise comparisons only showed a significant lower MVAS mean value in type 5 (‘Highly active type’) compared to type 3 (‘Daytime Sleepy Type’; *t* = 4.16; *p* < 0.001). Overall, there seemed to be a substantial degree of uncertainty in this general effect of chronotype, based on the 95% confidence intervals. The significant differences between the chronotypes for the mean KSS value are represented in Figure 3.

There were also significant interaction effects between condition and chronotype for the MVAS values and between time and chronotype for the RPE values. However, post hoc pairwise comparisons showed no clear differences in condition or time effects between chronotypes in both measures. 95% confidence intervals showed a relatively high degree of uncertainty in the effect.

### 3.3 Influence of Sleep Quality on Mental Fatigue Responses

Finally, the difference in effects of condition, time and their interaction between sleep quality groups can be found in Table 3.

**TABLE 3:** overview of the differences in mental fatigue variables between sleep quality groups.

Outcome	Type	Additional effect of sleep quality						*Covariate?*
		Estimate	95% CI	Test statistic	*p*	ES	Int.	
Subjective feeling of mental fatigue	General	0.89	[−0.14; 1.92]	5.21 (F)	**0.028** ^a^	0.12 (*η*_*p*_^2^)	Small	No for caffeine (*p* = 0.908)
	Interaction with condition	0.38	[−0.88; 1.63]	0.32 (F)	0.575	< 0.01 (*η*_*p*_^2^)	Trivial	
	Interaction with time	−0.40	[−1.65; 0.86]	1.24 (F)	0.269	0.01 (*η*_*p*_^2^)	Trivial	
	Interaction with condition*time	−0.23	[−2.01; 1.55]	0.06 (F)	0.800	< 0.01 (*η*_*p*_^2^)	Trivial	
Rating of perceived exertion	General	0.57	[−8.07; 9.21]	0.0002 (F)	0.989	< 0.01 (*η*_*p*_^2^)	Trivial	No for caffeine (*p* = 0.743)
	Interaction with condition	−2.00	[−10.78; 6.78]	1.86 (F)	0.174	0.004 (*η*_*p*_^2^)	Trivial	
	Interaction with time	T2: −4.00 T3: 0.71 T4: −1.43 T5: −2.95 T6: −3.67	[−12.78; 4.78] [−8.06; 9.49] [−10.21; 7.35] [−11.73; 5.83] [−12.45; 5.11]	0.80 (F)	0.554	0.009 (*η*_*p*_^2^)	Trivial	
	Interaction with condition*time	T2: 1.67 T3: 0.86 T4: 7.86 T5: 9.95 T6: 6.95	[−10.75; 14.08] [−11.56; 13.27] [−4.56; 20.27] [−2.46; 22.37] [−5.46; 19.37]	0.84 (F)	0.519	0.01 (*η*_*p*_^2^)	Trivial	
Go stimuli *z* score	General	0.14	[−0.26; 0.54]	1.25 (F)	0.271	0.03 (*η*_*p*_^2^)	Small	No for caffeine (*p* = 0.567)
	Interaction with condition	0.22	[−0.15; 0.59]	3.03 (F)	*0.084* ^b^	0.02 (*η*_*p*_^2^)	Small	
	Interaction with time	−0.14	[−0.51; 0.23]	0.82 (F)	0.366	< 0.01 (*η*_*p*_^2^)	Trivial	
	Interaction with condition*time	0.04	[−0.49; 0.56]	0.02 (F)	0.883	< 0.01 (*η*_*p*_^2^)	Trivial	
Time trial distance	General	−0.70	[−1.93; 0.53]	1.89 (F)	0.177	0.05 (*η*_*p*_^2^)	Small	No for caffeine (*p* = *0.065* ^b^)
	Interaction with condition	−0.29	[−0.84; 0.27]	1.03 (F)	0.316	0.03 (*η*_*p*_^2^)	Small	
Karolinska sleepiness scale	General	0.93	[−0.18; 2.06]	/	0.101	2.55 (OR)	Medium	No for caffeine (*p* = 0.805)
	Interaction with condition	−0.04	[−1.57; 1.48]	/	0.958	0.96 (OR)	Trivial	
	Interaction with time	0.19	[−1.35; 1.73]	/	0.811	1.21 (OR)	Small	
	Interaction with condition*time	0.26	[−1.91; 2.42]	/	0.817	1.29 (OR)	Small	

Abbreviations: CI = confidence interval; ES = effect size; Int. = interpretation.

^a^ Significant effect in bold.

^b^ Trend to significance in italics.

**FIGURE 4:**
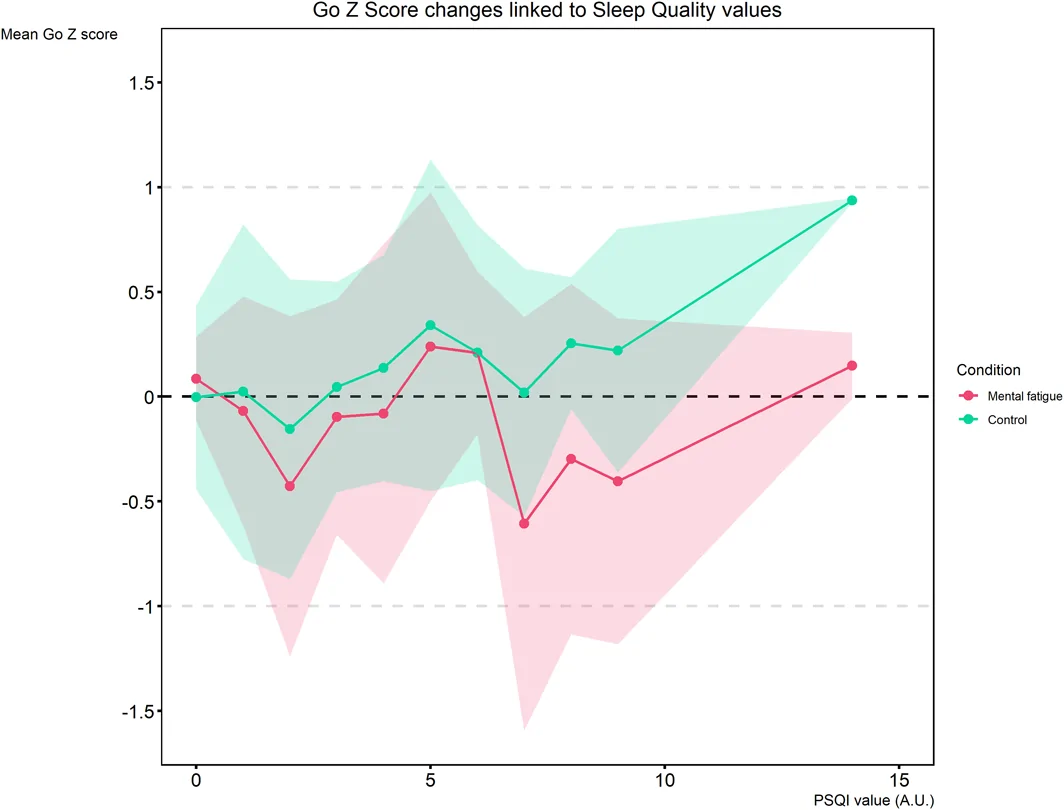
Changes in mean go z score (incorporating both the reaction time and accuracy) based on PSQI values. The points show the mean value, while the ribbon indicates the 95% confidence interval. (A.U. = arbitrary units; green = control condition; Red = intervention condition).

Results showed a significant increase in mean MVAS values in PSQI group 2 (‘bad sleep quality’) compared to group 1 (‘good sleep quality’). There were no other significant main or interaction effects related to the PSQI groups. Subsequent sensitivity analyses showed almost no differences in the conclusions when considering PSQI as a continuous variable. (all *p* > 0.05). Only the conclusion of the GoNoGo *z* score slightly changed, with a significant interaction effect between PSQI and condition (*F* = 7.26; *p* = 0.008; *ηp* ^2^ = 0.02). Figure 4 shows that the difference in *z* score between conditions increased with a rise in PSQI value.

## 4 Discussion

This is the first study to specifically investigate the differences in the subjective and behavioural response to MF between different aspects of sleep (i.e., sleep quality and chronotype), while controlling for self‐reported caffeine consumption. Overall, this exploratory study revealed little difference in MF response between the chronotype and sleep quality groups. When examining chronotype differences, only the MVAS measures showed a significant interaction with condition, with no notable effects present when examining post hoc comparisons. Similarly, there were almost no noteworthy differences in MF response between sleep quality groups. When considering PSQI to be a continuous value, the outcome did significantly influence the cognitive response to mental fatigue, with a decreasing mean *z* score in the intervention compared to the control condition with worse sleep quality. These findings actually seem to contradict the previously mentioned studies that propose that sleep/circadian variables and MF are inherently linked (Barbosa et al. 2025; Filipas et al. 2021; Laulan et al. 2025). This is further supported by the limited influence of caffeine consumption as a confounder variable. As this is an exploratory study however, it is difficult to make a definitive conclusion on the link between sleep/circadian variables and MF (Ditroilo et al. 2025). Therefore, this discussion will primarily focus on using the study's methodology and findings to allow future research to more profoundly establish the relationship between sleep behaviour/circadian rhythms and MF response.

The present study was conducted in healthy individuals, all of whom showed acceptable sleep quality scores. The mean PSQI score was 4.18, while the PSQI only labels individuals as poor sleepers when the score exceeds 5 (Buysse et al. 1989). Appropriate sleep quantity is proposed to be between seven and 8 hours (Hirshkowitz et al. 2015), with the mean of the study population being 7.43 hours. Consequently, these findings cannot be generalized to populations experiencing sleep disturbances, such as athletes during training or competition (Halson 2014). Although previous studies have experimentally examined the effects of sleep restriction on MF responses (Barbosa et al. 2025; Filipas et al. 2021), it remains important to investigate this relationship in applied settings. For example, Descotes et al. (2025) found a significant negative correlation between the sleep quality of elite ice hockey players and their subjective level of MF during the season. This possible link between sleep‐related variables and the level of MF was also brought up in focus group interviews in elite female football players (Thompson et al. 2021). As these findings differ from ours, future research should further explore the association between sleep‐related variables and MF response in specific populations with sleep disturbances, such as athletes, to further map the possible interactions at extreme ends of the spectrum.

The distinctions between groups were based on two subjective scales, one quantifying sleep quality and one quantifying chronotype. The PSQI was used as a measure because of its excellent reliability and validity in clinical and non‐clinical samples and because it is the most commonly used generic tool of sleep quality in practical and research settings (Mollayeva et al. 2016). Meanwhile, the SIC was chosen because of its ease of use in a study that already used a number of different questionnaires and other outcome measures (Putilov, Budkevich, et al. 2021; Putilov, Sveshnikov, et al. 2021). The original article also showed the construction of the SIC based on a number of other important chronotype measures, such as the visuo verbal judgement task (Putilov, Budkevich, et al. 2021; Putilov, Sveshnikov, et al. 2021). Based on the premise of the original study (Habay et al. 2025), we still believe that this was the best course of action to increase the feasibility and impact of this original aim. However, these questionnaires present with a number of limitations (e.g., response bias and misinterpretation (Hassan et al. 2023)). Overall, future hypothesis driven studies might benefit from using other, more extensive questionnaires to quantify these sleep‐related variables. For example, chronotype was now categorized into six specific profiles, which do not always accurately reflect an individuals' true chronotype and could have resulted in the observed null findings on the influence of chronotype on mental fatigue response. A possible alternative, albeit more time consuming, is the visuo‐verbal judgement task (Marcoen et al. 2015). This questionnaire assesses the level of tiredness at different increments over a 31‐h period of wakefulness, providing a detailed subjective measure of circadian flexibility (Marcoen et al. 2015). Another example, that is commonly used in athlete settings, is the Morningness‐Eveningness Questionnaire, which is highly correlated to the timing of melatonin release and core body changes throughout the day (Horne and Ostberg 1976; Kunorozva et al. 2017). However, it should also be mentioned that recent interpretations critique the use of these questionnaires to quantify something as complex as an individuals' chronotype, arguing that a multidimensional model with subjective and objective input should be used instead (Chauhan et al. 2023). In addition, physiological measures, such as polysomnography (Rundo and Downey 2019) or wearable devices (Asgari Mehrabadi et al. 2020; De Zambotti et al. 2019), provide more objective measures of sleep quality compared to the PSQI. If we truly want to understand the relationship between sleep quality and MF response in the future, studies should include a combination of objective and subjective sleep behaviour measures. Similarly, the caffeine consumption questionnaire was added as a covariate because caffeine affects both sleep behaviour (Clark and Landolt 2017) and MF response (Proost et al. 2022; Van Cutsem et al. 2018). However, a large degree of interindividual variability was observed, as the standard deviation (1406 mg/week) is almost equal to the mean value (1481 mg). This is to be expected, given the difficulty to accurately measure caffeine consumption due to, among other reasons, a variety in preparation methods and caffeine content of different beverages (Poole et al. 2019). A possible alternative could be to standardize caffeine intake in the days preceding and on the day of testing, to make sure that this confounder has a limited effect on sleep behaviour and MF response outcomes (while taking any type of withdrawal effect into account). It should also be acknowledged that some methodological choices that were made may have had a profound influence on the results. For example, by standardizing the timing of the sessions, participants whose chronotype was better aligned with the testing schedule may have been advantaged. Furthermore, by requiring participants to restrict their caffeine consumption before each session, regular caffeine users may have experienced greater difficulty concentrating during the various tasks. The same observation can be made regarding the requirements to get at least 7h of sleep. By requiring participants to get a good night's sleep, some of the variability observed in the PSQI values might have been reduced, leading to a limited effect of sleep quality on MF response. These considerations should be taken into account by future research that aims at further elucidating the link between sleep and circadian variables and MF.

The findings of this study provide further insights into the distinction between MF and acute sleepiness, which is crucial to inform practice on the use of different scales to quantify all aspects of fatigue and drowsiness within a population (Hu and Lodewijks 2020). This study further demonstrates that sleepiness variables should be added to future MF studies as potential confounding factors, as the group effects differed between KSS outcomes and the other collected variables. This recommendation aligns with recently published methodological guidelines targeting MF research in movement science (Schampheleer et al. 2025). These guidelines propose to quantify MF using at least two out of the three existing quantification categories (i.e., subjective, behavioural and physiological) and to combine it with several relevant confounders to make sure that any decrements in performance can be solely attributed to MF (Schampheleer et al. 2025). One of these confounders is sleepiness, which can be quantified in numerous ways, each with their own strengths and limitations (Johns 1998; Shahid et al. 2012). Of note, recent evidence implies that multidimensional scales are more comprehensive and reliable in distinguishing MF and sleepiness compared to unidimensional scales like the KSS (Hu and Lodewijks 2020). Together with the appropriate quantification of MF, future studies should include multiple multidimensional measures of sleepiness within trial protocols to completely map out its possible link with MF.

## 5 Conclusion

This exploratory study aimed to investigate the relationship between sleep and circadian‐related variables and the response to MF in healthy individuals. Secondary analyses of a previous large‐scale study were conducted, investigating possible differences in MF effects on subjective and behavioural variables between chronotype and sleep quality groups. In general, the differences in MF response between these groups were limited. However, one observation that can be made was that any found significant influences of sleep quality and chronotype on MF response were focused within the subjective measures, specifically the subjective feeling of mental fatigue and sleepiness. Overall, this study can be seen as one of many first steps that have been taken in better understanding the link between sleep behaviour/circadian rhythm variables and MF. Most importantly, our focus was to share our learnings to inform recommendations to further explore this potential interaction in future research, using adequate error‐controlled analyses and more fitting measurement methods and study populations.

## Funding

Jelle Habay is a recipient of a fundamental aspirant fellowship funded by the Research Foundation Flanders (FWO; 10.13039/501100003130) (project: 11J6323N).

## Ethics Statement

This study was approved by the commission of medical ethics at the UZ Brussel (B.UN. 1432022000084) in accordance with the declaration of Helsinki. Informed consent was obtained from the participants.

## Consent

All participants gave written informed consent to participate in the present study.

## Conflicts of Interest

The authors declare no conflicts of interest.

## Permission to Reproduce Material From Other Sources

The authors have nothing to report.

## Supporting information


Supporting Information S1

Supporting Information S2

Supporting Information S3

## Data Availability

The data that support the findings of this study are available in the supplementary material of this article and in previously published material.
