# Supplementary material for: No Influence of Sleep Quality and Chronotype on the Effects of Mental Fatigue: Evidence From an Exploratory Analysis
Source: Eur J Sport Sci. 2026 Sep 10;26(10):e70247. doi: 10.1002/ejsc.70247 (PMC13562610; doi:10.1002/ejsc.70247)
Supplement: Supplementary file 1 [file EJSC-26-e70247-s001.docx]

**SUPPLEMENTARY FILE 3**

Supplementary table 1: detailed overview of quantitative sleep behaviour-related variables (PSQI = Pittsburgh Sleep Quality Index)

| **Outcome variable** | *Mean ± SD* |
| --- | --- |
| **PSQI** |  |
| ● Overall sleep quality (A.U.) | 4.18 ± 2.23 |
| ● Overall sleep duration (hrs/night) | 7.43 ± 0.70 |
| ● Component 1 *(“Subjective Sleep Quality”;* A.U.) | 0.86 ± 0.58 |
| ● Component 2 *(“Sleep Latency”;* A.U.) | 0.92 ± 0.78 |
| ● Component 3 *(“Sleep Duration”;* A.U.) | 0.47 ± 0.63 |
| ● Component 4 *(“Habitual Sleep Deficiency”;* A.U.) | 0.19 ± 0.56 |
| ● Component 5 *(“Sleep Disturbances”;* A.U.) | 1.04 ± 0.34 |
| ● Component 6 *(“Sleep Medication Use”;* A.U.) | 0.11 ± 0.49 |
| ● Component 7 *(“Daytime dysfunction”;* A.U.) | 0.59 ± 0.57 |
| **Caffeine Consumption (mg)** | 1481 ± 1406 |

Supplementary figure 1: differences in condition effects over time between the different chronotypes. **A.** differences between chronotypes in condition Deltas over time in MVAS values. **B.** differences between chronotypes in condition Deltas over time in Go reaction time values. **C.** differences between chronotypes in condition Deltas over time in Go accuracy values. **D.** differences in mean time trial distance values between chronotypes; **E.** differences between chronotypes in condition Deltas over time in RPE values; **F.**. differences between chronotypes in condition Deltas over time in KSS values. Condition Deltas were always calculated as follows: mental fatigue – control. Panel A only shows four chronotypes as the results of the morning type overlap with the results of the highly active type. (A.U. = arbitrary units; MT = morning type; ET = evening type; DST = daytime sleepy type; DT = daytime type; HAT = highly active type)

Supplementary figure 2: differences in condition effects over time between the different sleep quality groups. **A.** differences between sleep quality groups in condition Deltas over time in MVAS values. **B.** differences between sleep quality groups in condition Deltas over time in Go reaction time values. **C.** differences between sleep quality groups in condition Deltas over time in Go accuracy values. **D.** differences in mean time trial distance values between sleep quality groups; **E.** differences between sleep quality groups in condition Deltas over time in RPE values; **F.**. differences between sleep quality groups in condition Deltas over time in KSS values. Condition Deltas were always calculated as follows: mental fatigue – control. “Good sleep quality” group = PSQI value lower or equal to 2; “Bad sleep quality” group = PSQI value higher or equal to 6.
