# Supplementary material for: No Influence of Sleep Quality and Chronotype on the Effects of Mental Fatigue: Evidence From an Exploratory Analysis
Source: Eur J Sport Sci. 2026 Sep 10;26(10):e70247. doi: 10.1002/ejsc.70247 (PMC13562610; doi:10.1002/ejsc.70247)
Supplement: Supplementary file 2 [file EJSC-26-e70247-s003.docx]

Supplementary File 2

### R code of EJSS study : No Influence of Sleep Quality and Chronotype on the Effects of Mental Fatigue: Evidence from an Exploratory Analysis

#### Jelle Habay

#### 2026-07-03

- Introduction
- Preparation
  - Set CRAN mirror
  - Installing packages
  - Loading packages
  - Data import
    - Original data
    - PSQI data
  - Completion of data files
    - General
    - Chronotype
    - PSQI
- Descriptives, baseline assessments and specific new data files
  - Individual features
  - Chronotype
    - Differences in descriptives between chronotypes
    - Subjective feeling of mental fatigue
    - Go Stimuli
    - Time trial
    - Rating of perceived exertion
    - Karolinska sleepiness scale
  - PSQI
    - Differences in descriptives between PSQI groups
    - Subjective feeling of mental fatigue
    - Go stimuli
    - Time trial
    - Rating of perceieved exertion
    - Karolinska sleepiness scale
  - Difference in mental fatigue variables between chronotype groups
    - Subjective feeling of mental fatigue
    - Go stimuli
    - Time trial
    - Rating of perceived exertion
    - Karolinska sleepiness scale
  - Difference in Mental Fatigue variables between PSQI groups
    - Subjective feeling of mental fatigue
    - Go stimuli
    - Time trial
    - Rating of perceived exertion
    - Karolinska sleepiness scale

# Introduction

This is an R Markdown document containing the code used during analysis of the following paper:

Habay et al. (2026): No Influence of Sleep Quality and Chronotype on the Effects of Mental Fatigue: Evidence from an Exploratory Analysis

The aim of this study was to exploratory assess how internal sleep variables (i.e. sleep quality and chronotype) influence the behavioural and subjective response to mental fatigue. Therefore, we conducted secondary analyses of a previously published randomized crossover trial (<https://doi.org/10.1249/mss.0000000000003852>). All previous material can be found within the supplementary material of this study.

Questions on the content of the present document are to be send to the corresponding author: [email protected]

# Preparation

## Set CRAN mirror

options(repos = c(CRAN = "https://cloud.r-project.org"))

## Installing packages

install.packages("readxl") #importing excel files

install.packages("tidyverse") #data wrangling

install.packages("hms") #connecting times of day in PSQI file

install.packages("skimr") #providing an overview of the data

install.packages("lme4") #performing linear mixed effect models

install.packages("lmerTest") #performing linear mixed effect models

install.packages("ordinal") #performing ordinal modelling

install.packages("emmeans") #estimated marginal means

install.packages("zoo") #predict missing values

install.packages("effectsize") #quantifying effect sizes

## Loading packages

library(readxl)

library(tidyverse)

library(hms)

library(skimr)

library(lme4)

library(lmerTest)

library(ordinal)

library(emmeans)

library(zoo)

library(effectsize)

## Data import

### Original data

This data is the original file that can be found in the original publication (see introduction). It contains the behavioural and subjective outcomes, as well as the overview of all collected individual features.

Results_MFINDDIFF <- read_excel("SF3_Results_MFINDDIFF.xlsx",

na = "9999")

### PSQI data

This data file provides a detailed overview of the answers to the PSQI questionnaire for every participant.

PSQI_overview <- read_excel("SF1_Results_PSQI.xlsx",

col_types = c("text", "date", "numeric",

"date", "date", "numeric", "numeric",

"numeric", "numeric", "numeric",

"numeric", "numeric", "numeric",

"numeric", "numeric", "text", "text",

"numeric", "numeric", "text", "numeric",

"numeric", "numeric", "numeric",

"numeric", "text", "date", "numeric",

"numeric", "numeric", "numeric",

"numeric", "numeric", "numeric",

"numeric", "numeric", "numeric",

"numeric", "numeric", "numeric",

"numeric", "numeric", "numeric",

"numeric", "numeric", "numeric",

"numeric", "numeric", "numeric",

"numeric", "numeric", "numeric",

"numeric"), na = "9999") %>%

mutate(

Bedtijd = as_hms(Bedtijd),

Wektijd = as_hms(Wektijd),

Uren_Slaap = as_hms(Uren_Slaap),

`Hrs_inBed(hr)` = as_hms(`Hrs_inBed(hr)`)

)

## Completion of data files

Separate data files were constructed to assess the difference in response between sleep variables.Additionally, the PSQI scores were evaluated to assess the appropriate division of a “good” and “bad” sleep quality group. We also chose to rescale the revised caffeine consumption questionnaire in all data files. Specific participants were also excluded in the time trial and rating of perceived exertion files based on inclusion remarks of the original study.

### General

Data_Sleep <- Results_MFINDDIFF[c(1, 14:70, 89:100, 101, 107, 177:180, 199:212)] %>% #one big file for all relevant outcomes of these exploratory analyses

dplyr::slice(-c(6, 13, 21, 30, 39, 42, 46, 50, 57, 68, 80, 95, 103)) %>% #na values overall or for one condition

dplyr::slice(-c(5, 17)) #dropout due to inclusion remarks

Data_PSQI <- PSQI_overview %>%

dplyr::slice(-c(5, 17))

Data_Sleep_IND <- Data_Sleep[c(1:32)] %>% #a separate file to assess the differences in descriptives between the groups

mutate(Sleep_duration = Data_PSQI$Hours_Sleeppernight) #with the addition of sleep duration

### Chronotype

The choice was made to exclude two participants from the chronotype analyses: one with a missing value, and the only participant that chose type 7.

Data_Sleep_IND_Chrono <- Data_Sleep_IND %>% #specific file to assess the differences in individual features between chronotypes (without NA and type 7)

dplyr::slice(-c(31, 71))

Data_Chronotype_MVASMC <- Data_Sleep[c(1, 3, 31, 32, 34, 37, 47, 50)] %>% #specific file to assess the differences in MVAS value between chronotypes (without NA and type 7)

dplyr::slice(-c(31, 71)) %>% #chronotype dropout

mutate(across(where(is.numeric) & !PP_number, ~ na.approx(., na.rm = FALSE))) %>% #imputing missing values of the MVAS

mutate(CCQ_z = scale(CCQ)) #rescaling the CCQ

Data_Chronotype_Go_zscore <- Data_Sleep[c(1, 3, 31, 32, 59, 60, 62, 63, 65, 66, 68, 69)] %>%

dplyr::slice(-c(31, 71)) %>% #chronotype dropout

mutate(CCQ_z = scale(CCQ)) #rescaling the CCQ

Data_Chronotype_TT <- Data_Sleep[c(1, 3, 31, 32, 71, 72)] %>% #specific file to assess the differences in time trial value between chronotypes (without NA and type 7)

dplyr::slice(-c(5)) %>% #extra NA

dplyr::slice(-c(24, 52, 58, 62, 79, 82)) %>% #inclusion remarks

dplyr::slice(-c(29, 66)) %>% #chronotype dropout

mutate(CCQ_z = scale(CCQ))

Data_Chronotype_RPE <- Data_Sleep[c(1, 3, 31, 32, 77:82, 84:89)] %>% #specific file to assess the differences in RPE value between chronotypes (without NA and type 7)

dplyr::slice(-c(31, 71)) %>% #chronotype dropout

mutate(CCQ_z = scale(CCQ)) #rescaling the CCQ

Data_Chronotype_KSS <- Data_Sleep[c(1, 3, 31, 32, 73:76)] %>% #specific file to assess the differences in KSS value between chronotypes (without NA and type 7)

dplyr::slice(-c(31, 71)) %>% #chronotype dropout

mutate(CCQ_z = scale(CCQ)) #rescaling the CCQ

### PSQI

A table was made to provide an overview of the number of participants with specific PSQI values

```
table(Data_Sleep_IND$PSQI)
##
##  0  1  2  3  4  5  6  7  8  9 14
##  2  5 14 22 23 15  6  6  5  3  1
```

Based on this table, the choice was made to only include participants with a score of lower or equal to 2 (“good” sleep quality group) and with a score of higher or equal to 6 (“bad” sleep quality group). This provided us with two groups with an appropriate difference in values, and an equal amount of participants for every group. This division is further visualized in the figure below.

```
## Warning: The dot-dot notation (`..count..`) was deprecated in ggplot2 3.4.0.
## ℹ Please use `after_stat(count)` instead.
```

```
## This warning is displayed once per session.
## Call `lifecycle::last_lifecycle_warnings()` to see where this warning was
## generated.
```

This division was also applied to the PSQI data files.

Data_Sleep_IND_PSQI <- Data_Sleep_IND %>%

filter(PSQI >= 6 | PSQI <= 2) %>%

mutate(PSQI_group = case_when(PSQI <= 2 ~ 1, PSQI >= 6 ~ 2))

Data_PSQI_MVASMC <- Data_Sleep[c(1, 30, 32, 34, 37, 47, 50)] %>%

mutate(CCQ_z = scale(CCQ)) %>%

filter(PSQI >= 6 | PSQI <= 2) %>%

mutate(PSQI_group = case_when(PSQI <= 2 ~ 1, PSQI >= 6 ~ 2))

Data_PSQI_Go_zscore <- Data_Sleep[c(1, 30, 32, 59, 60, 62, 63, 65, 66, 68, 69)]%>%

mutate(CCQ_z = scale(CCQ)) %>%

filter(PSQI >= 6 | PSQI <= 2) %>%

mutate(PSQI_group = case_when(PSQI <= 2 ~ 1, PSQI >= 6 ~ 2))

Data_PSQI_TT <- Data_Sleep[c(1, 30, 32, 71, 72)] %>%

dplyr::slice(-c(5, 25, 53, 59, 63, 80, 83)) %>%

mutate(CCQ_z = scale(CCQ)) %>%

filter(PSQI >= 6 | PSQI <= 2) %>%

mutate(PSQI_group = case_when(PSQI <= 2 ~ 1, PSQI >= 6 ~ 2))

Data_PSQI_RPE <- Data_Sleep[c(1, 30, 32, 77:82, 84:89)] %>%

mutate(CCQ_z = scale(CCQ)) %>%

filter(PSQI >= 6 | PSQI <= 2) %>%

mutate(PSQI_group = case_when(PSQI <= 2 ~ 1, PSQI >= 6 ~ 2))

Data_PSQI_KSS <- Data_Sleep[c(1, 30, 32, 73:76)] %>%

mutate(CCQ_z = scale(CCQ)) %>%

filter(PSQI >= 6 | PSQI <= 2) %>%

mutate(PSQI_group = case_when(PSQI <= 2 ~ 1, PSQI >= 6 ~ 2))

To assess PSQI also as a continuous variable, the following data files were also constructed

Data_PSQI_MVASMC_cont <- Data_Sleep[c(1, 30, 32, 34, 37, 47, 50)] %>%

mutate(CCQ_z = scale(CCQ))

Data_PSQI_Go_zscore_cont <- Data_Sleep[c(1, 30, 32, 59, 60, 62, 63, 65, 66, 68, 69)]%>%

mutate(CCQ_z = scale(CCQ))

Data_PSQI_TT_cont <- Data_Sleep[c(1, 30, 32, 71, 72)] %>%

dplyr::slice(-c(5, 25, 53, 59, 63, 80, 83)) %>%

mutate(CCQ_z = scale(CCQ))

Data_PSQI_RPE_cont <- Data_Sleep[c(1, 30, 32, 77:82, 84:89)] %>%

mutate(CCQ_z = scale(CCQ))

Data_PSQI_KSS_cont <- Data_Sleep[c(1, 30, 32, 73:76)] %>%

mutate(CCQ_z = scale(CCQ))

# Descriptives, baseline assessments and specific new data files

## Individual features

Overview in descriptives of individual features and the overall PSQI results

skim(Data_Sleep_IND)

| Data summary | |
| --- | --- |
| Name | Data_Sleep_IND |
| Number of rows | 102 |
| Number of columns | 33 |
| _______________________ |  |
| Column type frequency: |  |
| character | 4 |
| numeric | 29 |
| ________________________ |  |
| Group variables | None |

****Variable type: character****

| **skim_variable** | **n_missing** | **complete_rate** | **min** | **max** | **empty** | **n_unique** | **whitespace** |
| --- | --- | --- | --- | --- | --- | --- | --- |
| PP_number | 0 | 1.00 | 16 | 16 | 0 | 102 | 0 |
| Geslacht | 0 | 1.00 | 1 | 1 | 0 | 2 | 0 |
| Practised Sport | 4 | 0.96 | 4 | 22 | 0 | 33 | 0 |
| Open_Closed | 6 | 0.94 | 4 | 6 | 0 | 3 | 0 |

****Variable type: numeric****

| **skim_variable** | **n_missing** | **complete_rate** | **mean** | **sd** | **p0** | **p25** | **p50** | **p75** | **p100** | **hist** |
| --- | --- | --- | --- | --- | --- | --- | --- | --- | --- | --- |
| Leeftijd | 0 | 1.00 | 32.60 | 8.54 | 20.00 | 26.00 | 31.00 | 39.00 | 50.00 | ▇▇▆▅▃ |
| Lengte | 0 | 1.00 | 175.09 | 9.85 | 156.00 | 168.00 | 175.00 | 181.75 | 200.00 | ▃▇▇▅▂ |
| Gewicht | 0 | 1.00 | 70.17 | 11.70 | 43.60 | 61.87 | 69.50 | 77.97 | 99.20 | ▂▇▇▆▂ |
| BMI | 0 | 1.00 | 22.81 | 2.61 | 17.03 | 20.86 | 22.98 | 24.20 | 29.16 | ▂▆▇▃▁ |
| Vet%(Calc) | 2 | 0.98 | 20.87 | 7.45 | 6.68 | 14.89 | 20.23 | 25.86 | 40.35 | ▃▇▆▅▁ |
| Vet%(Dexa) | 0 | 1.00 | 20.53 | 7.75 | 7.40 | 14.60 | 18.90 | 26.50 | 38.50 | ▃▇▃▃▂ |
| VO2Max | 0 | 1.00 | 3.38 | 0.87 | 1.61 | 2.62 | 3.52 | 4.08 | 5.40 | ▅▇▆▇▂ |
| rVO2Max | 0 | 1.00 | 48.05 | 9.47 | 29.00 | 41.00 | 47.00 | 54.00 | 68.00 | ▃▇▇▆▃ |
| PPO | 0 | 1.00 | 255.59 | 63.94 | 140.00 | 200.00 | 260.00 | 320.00 | 380.00 | ▃▇▃▇▃ |
| rPPO | 0 | 1.00 | 3.64 | 0.70 | 2.23 | 3.12 | 3.62 | 4.22 | 5.37 | ▃▇▇▆▂ |
| PerformanceLevel | 0 | 1.00 | 2.16 | 0.93 | 1.00 | 2.00 | 2.00 | 3.00 | 5.00 | ▃▇▃▁▁ |
| IPAQ (MET-min/week) | 0 | 1.00 | 3973.85 | 2752.10 | 504.00 | 2238.75 | 3486.00 | 4681.50 | 15504.00 | ▇▆▁▁▁ |
| IPAQ (kcal/week) | 0 | 1.00 | 4736.00 | 3411.60 | 479.73 | 2416.61 | 4104.01 | 5884.42 | 17002.72 | ▇▆▂▁▁ |
| YearsOfExperience | 5 | 0.95 | 7.61 | 7.18 | 0.00 | 2.50 | 5.00 | 12.00 | 30.00 | ▇▂▂▁▁ |
| PSF | 6 | 0.94 | 1.96 | 0.81 | 1.00 | 1.00 | 2.00 | 3.00 | 3.00 | ▇▁▇▁▇ |
| SPQ | 0 | 1.00 | 1.30 | 0.94 | 0.00 | 1.00 | 1.00 | 2.00 | 4.00 | ▅▇▆▂▁ |
| SART(ACC) | 0 | 1.00 | 0.57 | 0.19 | 0.04 | 0.42 | 0.58 | 0.75 | 0.88 | ▁▃▇▇▇ |
| NBACK(ACC) | 0 | 1.00 | 0.84 | 0.10 | 0.44 | 0.79 | 0.84 | 0.91 | 0.97 | ▁▁▂▇▇ |
| NBACK(RT(ms)) | 0 | 1.00 | 840.88 | 249.79 | 394.89 | 639.46 | 810.73 | 1030.41 | 1461.69 | ▅▇▆▅▂ |
| PVT(FA) | 0 | 1.00 | 0.30 | 0.87 | 0.00 | 0.00 | 0.00 | 0.00 | 6.00 | ▇▁▁▁▁ |
| PVT(Lap) | 0 | 1.00 | 0.03 | 0.17 | 0.00 | 0.00 | 0.00 | 0.00 | 1.00 | ▇▁▁▁▁ |
| PVT(RT(ms)) | 0 | 1.00 | 288.79 | 27.10 | 227.29 | 271.01 | 287.28 | 305.02 | 380.65 | ▂▇▆▂▁ |
| SAI | 1 | 0.99 | 32.01 | 6.86 | 22.00 | 27.00 | 32.00 | 36.00 | 54.00 | ▇▇▅▁▁ |
| BSS | 2 | 0.98 | 3.57 | 0.58 | 2.23 | 3.21 | 3.62 | 4.00 | 4.69 | ▂▅▇▇▃ |
| MT | 0 | 1.00 | 5.13 | 0.65 | 3.38 | 4.75 | 5.12 | 5.59 | 6.50 | ▂▃▇▆▂ |
| PSQI | 0 | 1.00 | 4.17 | 2.23 | 0.00 | 3.00 | 4.00 | 5.00 | 14.00 | ▃▇▂▁▁ |
| Chronotype | 1 | 0.99 | 2.96 | 1.46 | 1.00 | 2.00 | 3.00 | 4.00 | 7.00 | ▇▆▂▅▁ |
| CCQ | 0 | 1.00 | 1481.15 | 1405.53 | 0.00 | 390.39 | 1265.43 | 2088.36 | 6309.93 | ▇▅▂▁▁ |
| Sleep_duration | 0 | 1.00 | 7.43 | 0.77 | 5.00 | 7.00 | 7.50 | 8.00 | 9.50 | ▁▂▇▆▁ |

```
table(Data_Sleep_IND$Chronotype)
##
##  1  2  3  4  5  7
## 22 17 28 13 20  1
summary(Data_PSQI)
##  Proefpersoonnummer    Bedtijd                Minuten_Inslaapvallen
##  Length:102         Min.   :00:00:00.000000   Min.   : 0.00
##  Class :character   1st Qu.:22:00:00.000000   1st Qu.: 5.00
##  Mode  :character   Median :22:30:00.000000   Median :12.50
##                     Mean   :17:56:28.235294   Mean   :15.51
##                     3rd Qu.:23:00:00.000000   3rd Qu.:20.00
##                     Max.   :23:55:00.000000   Max.   :90.00
##
##     Wektijd                  Uren_Slaap
##  Min.   :05:00:00.000000   Min.   :05:00:00.000000
##  1st Qu.:06:30:00.000000   1st Qu.:07:00:00.000000
##  Median :07:00:00.000000   Median :07:30:00.000000
##  Mean   :07:11:58.823529   Mean   :07:25:52.941176
##  3rd Qu.:07:45:00.000000   3rd Qu.:08:00:00.000000
##  Max.   :10:00:00.000000   Max.   :09:30:00.000000
##
##  Hoe vaak had u tijdens de voorbije maanden moeilijkheden met slapen omdat u: 30 min
##  Min.   :1.000
##  1st Qu.:1.000
##  Median :2.000
##  Mean   :1.961
##  3rd Qu.:2.000
##  Max.   :4.000
##
##  Hoe vaak had u tijdens de voorbije maanden moeilijkheden met slapen omdat u: Midden in de nacht
##  Min.   :1.000
##  1st Qu.:2.000
##  Median :3.000
##  Mean   :2.569
##  3rd Qu.:3.000
##  Max.   :4.000
##
##  Hoe vaak had u tijdens de voorbije maanden moeilijkheden met slapen omdat u: Toilet
##  Min.   :1.000
##  1st Qu.:1.000
##  Median :2.000
##  Mean   :2.098
##  3rd Qu.:3.000
##  Max.   :4.000
##
##  Hoe vaak had u tijdens de voorbije maanden moeilijkheden met slapen omdat u: Ademhalen
##  Min.   :1.00
##  1st Qu.:1.00
##  Median :1.00
##  Mean   :1.02
##  3rd Qu.:1.00
##  Max.   :2.00
##
##  Hoe vaak had u tijdens de voorbije maanden moeilijkheden met slapen omdat u: Snurken
##  Min.   :1.000
##  1st Qu.:1.000
##  Median :1.000
##  Mean   :1.275
##  3rd Qu.:1.000
##  Max.   :3.000
##
##  Hoe vaak had u tijdens de voorbije maanden moeilijkheden met slapen omdat u: Koud
##  Min.   :1.000
##  1st Qu.:1.000
##  Median :1.000
##  Mean   :1.284
##  3rd Qu.:2.000
##  Max.   :3.000
##
##  Hoe vaak had u tijdens de voorbije maanden moeilijkheden met slapen omdat u: Warm
##  Min.   :1.000
##  1st Qu.:1.000
##  Median :2.000
##  Mean   :1.755
##  3rd Qu.:2.000
##  Max.   :4.000
##
##  Hoe vaak had u tijdens de voorbije maanden moeilijkheden met slapen omdat u: Nachtmerries
##  Min.   :1.000
##  1st Qu.:1.000
##  Median :1.000
##  Mean   :1.333
##  3rd Qu.:2.000
##  Max.   :4.000
##
##  Hoe vaak had u tijdens de voorbije maanden moeilijkheden met slapen omdat u: Pijn
##  Min.   :1.000
##  1st Qu.:1.000
##  Median :1.000
##  Mean   :1.127
##  3rd Qu.:1.000
##  Max.   :3.000
##
##  Hoe vaak had u tijdens de voorbije maanden moeilijkheden met slapen omdat u: Andere
##  Min.   :1.000
##  1st Qu.:1.000
##  Median :1.000
##  Mean   :1.294
##  3rd Qu.:1.000
##  Max.   :4.000
##
##   Omschrijf         Slaapkwaliteit_VoorbijeMaand Geneesmiddelen
##  Length:102         Length:102                   Min.   :1.000
##  Class :character   Class :character             1st Qu.:1.000
##  Mode  :character   Mode  :character             Median :1.000
##                                                  Mean   :1.108
##                                                  3rd Qu.:1.000
##                                                  Max.   :4.000
##
##  Wakker_Blijven  Enthousiasme
##  Min.   :1.000   Length:102
##  1st Qu.:1.000   Class :character
##  Median :1.000   Mode  :character
##  Mean   :1.294
##  3rd Qu.:2.000
##  Max.   :3.000
##
##  Indien u een kamergenoot of bedpartner heeft, vraag hem/haar hoe vaak u tijdens de voorbije maand: Snurkte
##  Min.   :1.000
##  1st Qu.:1.000
##  Median :1.000
##  Mean   :1.414
##  3rd Qu.:2.000
##  Max.   :4.000
##  NA's   :15
##  Indien u een kamergenoot of bedpartner heeft, vraag hem/haar hoe vaak u tijdens de voorbije maand: Ademhalingspauzes
##  Min.   :1.000
##  1st Qu.:1.000
##  Median :1.000
##  Mean   :1.092
##  3rd Qu.:1.000
##  Max.   :3.000
##  NA's   :15
##  Indien u een kamergenoot of bedpartner heeft, vraag hem/haar hoe vaak u tijdens de voorbije maand: Trekkende/Schokkende benen
##  Min.   :1.000
##  1st Qu.:1.000
##  Median :1.000
##  Mean   :1.471
##  3rd Qu.:2.000
##  Max.   :4.000
##  NA's   :15
##  Indien u een kamergenoot of bedpartner heeft, vraag hem/haar hoe vaak u tijdens de voorbije maand: Verwardheid
##  Min.   :1.000
##  1st Qu.:1.000
##  Median :1.000
##  Mean   :1.149
##  3rd Qu.:1.000
##  Max.   :4.000
##  NA's   :15
##  Indien u een kamergenoot of bedpartner heeft, vraag hem/haar hoe vaak u tijdens de voorbije maand: Andere Rusteloosheid
##  Min.   :1.000
##  1st Qu.:1.000
##  Median :1.000
##  Mean   :1.368
##  3rd Qu.:1.000
##  Max.   :4.000
##  NA's   :15
##  Indien u een kamergenoot of bedpartner heeft, vraag hem/haar hoe vaak u tijdens de voorbije maand: Omschrijf
##  Length:102
##  Class :character
##  Mode  :character
##
##
##
##
##  Hrs_inBed(hr)             Hrs_inBed(nr)     Mins_ToSleep
##  Min.   :06:00:00.000000   Min.   : 6.000   Min.   :0.0000
##  1st Qu.:07:30:00.000000   1st Qu.: 7.500   1st Qu.:0.0000
##  Median :08:00:00.000000   Median : 8.000   Median :0.0000
##  Mean   :08:04:55.294118   Mean   : 8.082   Mean   :0.3627
##  3rd Qu.:08:30:00.000000   3rd Qu.: 8.500   3rd Qu.:1.0000
##  Max.   :10:30:00.000000   Max.   :10.500   Max.   :3.0000
##
##  Hours_Sleeppernight Hours_Sleeppernight_Score  Sleep30mins         WakeUp
##  Min.   :5.000       Min.   :0.0000            Min.   :0.0000   Min.   :0.000
##  1st Qu.:7.000       1st Qu.:0.0000            1st Qu.:0.0000   1st Qu.:1.000
##  Median :7.500       Median :0.0000            Median :1.0000   Median :2.000
##  Mean   :7.431       Mean   :0.4706            Mean   :0.9608   Mean   :1.569
##  3rd Qu.:8.000       3rd Qu.:1.0000            3rd Qu.:1.0000   3rd Qu.:2.000
##  Max.   :9.500       Max.   :2.0000            Max.   :3.0000   Max.   :3.000
##
##  GetUpBathroom   BreatheComfort      CoughScore        FeelCold
##  Min.   :0.000   Min.   :0.00000   Min.   :0.0000   Min.   :0.0000
##  1st Qu.:0.000   1st Qu.:0.00000   1st Qu.:0.0000   1st Qu.:0.0000
##  Median :1.000   Median :0.00000   Median :0.0000   Median :0.0000
##  Mean   :1.098   Mean   :0.01961   Mean   :0.2745   Mean   :0.2843
##  3rd Qu.:2.000   3rd Qu.:0.00000   3rd Qu.:0.0000   3rd Qu.:1.0000
##  Max.   :3.000   Max.   :1.00000   Max.   :2.0000   Max.   :2.0000
##
##     FeelHot         BadDreams         HavePain      OtherReasonScale
##  Min.   :0.0000   Min.   :0.0000   Min.   :0.0000   Min.   :0.0000
##  1st Qu.:0.0000   1st Qu.:0.0000   1st Qu.:0.0000   1st Qu.:0.0000
##  Median :1.0000   Median :0.0000   Median :0.0000   Median :0.0000
##  Mean   :0.7549   Mean   :0.3333   Mean   :0.1275   Mean   :0.2941
##  3rd Qu.:1.0000   3rd Qu.:1.0000   3rd Qu.:0.0000   3rd Qu.:0.0000
##  Max.   :3.0000   Max.   :3.0000   Max.   :2.0000   Max.   :3.0000
##
##  SleepQualityRate MedicineTaken    TroubleDriveEatSocial ProblemEnthusiasm
##  Min.   :0.0000   Min.   :0.0000   Min.   :0.0000        Min.   :0.0
##  1st Qu.:1.0000   1st Qu.:0.0000   1st Qu.:0.0000        1st Qu.:0.0
##  Median :1.0000   Median :0.0000   Median :0.0000        Median :0.0
##  Mean   :0.8627   Mean   :0.1078   Mean   :0.2941        Mean   :0.5
##  3rd Qu.:1.0000   3rd Qu.:0.0000   3rd Qu.:1.0000        3rd Qu.:1.0
##  Max.   :3.0000   Max.   :3.0000   Max.   :2.0000        Max.   :2.0
##
##  C1_SubjSleepQ     C2_SleepLat      C3_Sleepdur      C4_Sleepeff
##  Min.   :0.0000   Min.   :0.0000   Min.   :0.0000   Min.   :0.0000
##  1st Qu.:1.0000   1st Qu.:0.0000   1st Qu.:0.0000   1st Qu.:0.0000
##  Median :1.0000   Median :1.0000   Median :0.0000   Median :0.0000
##  Mean   :0.8627   Mean   :0.9216   Mean   :0.4706   Mean   :0.1863
##  3rd Qu.:1.0000   3rd Qu.:1.0000   3rd Qu.:1.0000   3rd Qu.:0.0000
##  Max.   :3.0000   Max.   :3.0000   Max.   :2.0000   Max.   :3.0000
##
##   C5_Sleepdist    C6_Sleepmed     C7_Daytimedys     Global_Score
##  Min.   :0.000   Min.   :0.0000   Min.   :0.0000   Min.   : 0.000
##  1st Qu.:1.000   1st Qu.:0.0000   1st Qu.:0.0000   1st Qu.: 3.000
##  Median :1.000   Median :0.0000   Median :1.0000   Median : 4.000
##  Mean   :1.039   Mean   :0.1078   Mean   :0.5882   Mean   : 4.176
##  3rd Qu.:1.000   3rd Qu.:0.0000   3rd Qu.:1.0000   3rd Qu.: 5.000
##  Max.   :2.000   Max.   :3.0000   Max.   :2.0000   Max.   :14.000
##
skim(Data_PSQI)
```

| Data summary | |
| --- | --- |
| Name | Data_PSQI |
| Number of rows | 102 |
| Number of columns | 53 |
| _______________________ |  |
| Column type frequency: |  |
| character | 5 |
| difftime | 4 |
| numeric | 44 |
| ________________________ |  |
| Group variables | None |

****Variable type: character****

| **skim_variable** | **n_missing** | **complete_rate** | **min** | **max** | **empty** | **n_unique** | **whitespace** |
| --- | --- | --- | --- | --- | --- | --- | --- |
| Proefpersoonnummer | 0 | 1.00 | 15 | 15 | 0 | 102 | 0 |
| Omschrijf | 86 | 0.16 | 4 | 86 | 0 | 14 | 0 |
| Slaapkwaliteit_VoorbijeMaand | 0 | 1.00 | 9 | 15 | 0 | 4 | 0 |
| Enthousiasme | 0 | 1.00 | 19 | 25 | 0 | 3 | 0 |
| Indien u een kamergenoot of bedpartner heeft, vraag hem/haar hoe vaak u tijdens de voorbije maand: Omschrijf | 84 | 0.18 | 6 | 42 | 0 | 17 | 0 |

****Variable type: difftime****

| **skim_variable** | **n_missing** | **complete_rate** | **min** | **max** | **median** | **n_unique** |
| --- | --- | --- | --- | --- | --- | --- |
| Bedtijd | 0 | 1 | 0 secs | 86100 secs | 81000 secs | 17 |
| Wektijd | 0 | 1 | 18000 secs | 36000 secs | 25200 secs | 26 |
| Uren_Slaap | 0 | 1 | 18000 secs | 34200 secs | 27000 secs | 15 |
| Hrs_inBed(hr) | 0 | 1 | 21600 secs | 37800 secs | 28800 secs | 28 |

****Variable type: numeric****

| **skim_variable** | **n_missing** | **complete_rate** | **mean** | **sd** | **p0** | **p25** | **p50** | **p75** | **p100** | **hist** |
| --- | --- | --- | --- | --- | --- | --- | --- | --- | --- | --- |
| Minuten_Inslaapvallen | 0 | 1.00 | 15.51 | 14.03 | 0 | 5.0 | 12.5 | 20.0 | 90.0 | ▇▂▁▁▁ |
| Hoe vaak had u tijdens de voorbije maanden moeilijkheden met slapen omdat u: 30 min | 0 | 1.00 | 1.96 | 0.88 | 1 | 1.0 | 2.0 | 2.0 | 4.0 | ▅▇▁▂▂ |
| Hoe vaak had u tijdens de voorbije maanden moeilijkheden met slapen omdat u: Midden in de nacht | 0 | 1.00 | 2.57 | 1.01 | 1 | 2.0 | 3.0 | 3.0 | 4.0 | ▅▇▁▇▆ |
| Hoe vaak had u tijdens de voorbije maanden moeilijkheden met slapen omdat u: Toilet | 0 | 1.00 | 2.10 | 1.15 | 1 | 1.0 | 2.0 | 3.0 | 4.0 | ▇▅▁▂▃ |
| Hoe vaak had u tijdens de voorbije maanden moeilijkheden met slapen omdat u: Ademhalen | 0 | 1.00 | 1.02 | 0.14 | 1 | 1.0 | 1.0 | 1.0 | 2.0 | ▇▁▁▁▁ |
| Hoe vaak had u tijdens de voorbije maanden moeilijkheden met slapen omdat u: Snurken | 0 | 1.00 | 1.27 | 0.55 | 1 | 1.0 | 1.0 | 1.0 | 3.0 | ▇▁▂▁▁ |
| Hoe vaak had u tijdens de voorbije maanden moeilijkheden met slapen omdat u: Koud | 0 | 1.00 | 1.28 | 0.50 | 1 | 1.0 | 1.0 | 2.0 | 3.0 | ▇▁▂▁▁ |
| Hoe vaak had u tijdens de voorbije maanden moeilijkheden met slapen omdat u: Warm | 0 | 1.00 | 1.75 | 0.85 | 1 | 1.0 | 2.0 | 2.0 | 4.0 | ▇▅▁▃▁ |
| Hoe vaak had u tijdens de voorbije maanden moeilijkheden met slapen omdat u: Nachtmerries | 0 | 1.00 | 1.33 | 0.60 | 1 | 1.0 | 1.0 | 2.0 | 4.0 | ▇▂▁▁▁ |
| Hoe vaak had u tijdens de voorbije maanden moeilijkheden met slapen omdat u: Pijn | 0 | 1.00 | 1.13 | 0.36 | 1 | 1.0 | 1.0 | 1.0 | 3.0 | ▇▁▁▁▁ |
| Hoe vaak had u tijdens de voorbije maanden moeilijkheden met slapen omdat u: Andere | 0 | 1.00 | 1.29 | 0.77 | 1 | 1.0 | 1.0 | 1.0 | 4.0 | ▇▁▁▁▁ |
| Geneesmiddelen | 0 | 1.00 | 1.11 | 0.49 | 1 | 1.0 | 1.0 | 1.0 | 4.0 | ▇▁▁▁▁ |
| Wakker_Blijven | 0 | 1.00 | 1.29 | 0.50 | 1 | 1.0 | 1.0 | 2.0 | 3.0 | ▇▁▃▁▁ |
| Indien u een kamergenoot of bedpartner heeft, vraag hem/haar hoe vaak u tijdens de voorbije maand: Snurkte | 15 | 0.85 | 1.41 | 0.64 | 1 | 1.0 | 1.0 | 2.0 | 4.0 | ▇▃▁▁▁ |
| Indien u een kamergenoot of bedpartner heeft, vraag hem/haar hoe vaak u tijdens de voorbije maand: Ademhalingspauzes | 15 | 0.85 | 1.09 | 0.33 | 1 | 1.0 | 1.0 | 1.0 | 3.0 | ▇▁▁▁▁ |
| Indien u een kamergenoot of bedpartner heeft, vraag hem/haar hoe vaak u tijdens de voorbije maand: Trekkende/Schokkende benen | 15 | 0.85 | 1.47 | 0.87 | 1 | 1.0 | 1.0 | 2.0 | 4.0 | ▇▂▁▁▁ |
| Indien u een kamergenoot of bedpartner heeft, vraag hem/haar hoe vaak u tijdens de voorbije maand: Verwardheid | 15 | 0.85 | 1.15 | 0.52 | 1 | 1.0 | 1.0 | 1.0 | 4.0 | ▇▁▁▁▁ |
| Indien u een kamergenoot of bedpartner heeft, vraag hem/haar hoe vaak u tijdens de voorbije maand: Andere Rusteloosheid | 15 | 0.85 | 1.37 | 0.78 | 1 | 1.0 | 1.0 | 1.0 | 4.0 | ▇▁▁▁▁ |
| Hrs_inBed(nr) | 0 | 1.00 | 8.08 | 0.77 | 6 | 7.5 | 8.0 | 8.5 | 10.5 | ▁▅▇▃▁ |
| Mins_ToSleep | 0 | 1.00 | 0.36 | 0.64 | 0 | 0.0 | 0.0 | 1.0 | 3.0 | ▇▂▁▁▁ |
| Hours_Sleeppernight | 0 | 1.00 | 7.43 | 0.77 | 5 | 7.0 | 7.5 | 8.0 | 9.5 | ▁▂▇▆▁ |
| Hours_Sleeppernight_Score | 0 | 1.00 | 0.47 | 0.62 | 0 | 0.0 | 0.0 | 1.0 | 2.0 | ▇▁▅▁▁ |
| Sleep30mins | 0 | 1.00 | 0.96 | 0.88 | 0 | 0.0 | 1.0 | 1.0 | 3.0 | ▅▇▁▂▂ |
| WakeUp | 0 | 1.00 | 1.57 | 1.01 | 0 | 1.0 | 2.0 | 2.0 | 3.0 | ▅▇▁▇▆ |
| GetUpBathroom | 0 | 1.00 | 1.10 | 1.15 | 0 | 0.0 | 1.0 | 2.0 | 3.0 | ▇▅▁▂▃ |
| BreatheComfort | 0 | 1.00 | 0.02 | 0.14 | 0 | 0.0 | 0.0 | 0.0 | 1.0 | ▇▁▁▁▁ |
| CoughScore | 0 | 1.00 | 0.27 | 0.55 | 0 | 0.0 | 0.0 | 0.0 | 2.0 | ▇▁▂▁▁ |
| FeelCold | 0 | 1.00 | 0.28 | 0.50 | 0 | 0.0 | 0.0 | 1.0 | 2.0 | ▇▁▂▁▁ |
| FeelHot | 0 | 1.00 | 0.75 | 0.85 | 0 | 0.0 | 1.0 | 1.0 | 3.0 | ▇▅▁▃▁ |
| BadDreams | 0 | 1.00 | 0.33 | 0.60 | 0 | 0.0 | 0.0 | 1.0 | 3.0 | ▇▂▁▁▁ |
| HavePain | 0 | 1.00 | 0.13 | 0.36 | 0 | 0.0 | 0.0 | 0.0 | 2.0 | ▇▁▁▁▁ |
| OtherReasonScale | 0 | 1.00 | 0.29 | 0.77 | 0 | 0.0 | 0.0 | 0.0 | 3.0 | ▇▁▁▁▁ |
| SleepQualityRate | 0 | 1.00 | 0.86 | 0.58 | 0 | 1.0 | 1.0 | 1.0 | 3.0 | ▃▇▁▁▁ |
| MedicineTaken | 0 | 1.00 | 0.11 | 0.49 | 0 | 0.0 | 0.0 | 0.0 | 3.0 | ▇▁▁▁▁ |
| TroubleDriveEatSocial | 0 | 1.00 | 0.29 | 0.50 | 0 | 0.0 | 0.0 | 1.0 | 2.0 | ▇▁▃▁▁ |
| ProblemEnthusiasm | 0 | 1.00 | 0.50 | 0.61 | 0 | 0.0 | 0.0 | 1.0 | 2.0 | ▇▁▆▁▁ |
| C1_SubjSleepQ | 0 | 1.00 | 0.86 | 0.58 | 0 | 1.0 | 1.0 | 1.0 | 3.0 | ▃▇▁▁▁ |
| C2_SleepLat | 0 | 1.00 | 0.92 | 0.78 | 0 | 0.0 | 1.0 | 1.0 | 3.0 | ▃▇▁▁▁ |
| C3_Sleepdur | 0 | 1.00 | 0.47 | 0.62 | 0 | 0.0 | 0.0 | 1.0 | 2.0 | ▇▁▅▁▁ |
| C4_Sleepeff | 0 | 1.00 | 0.19 | 0.56 | 0 | 0.0 | 0.0 | 0.0 | 3.0 | ▇▁▁▁▁ |
| C5_Sleepdist | 0 | 1.00 | 1.04 | 0.34 | 0 | 1.0 | 1.0 | 1.0 | 2.0 | ▁▁▇▁▁ |
| C6_Sleepmed | 0 | 1.00 | 0.11 | 0.49 | 0 | 0.0 | 0.0 | 0.0 | 3.0 | ▇▁▁▁▁ |
| C7_Daytimedys | 0 | 1.00 | 0.59 | 0.57 | 0 | 0.0 | 1.0 | 1.0 | 2.0 | ▇▁▇▁▁ |
| Global_Score | 0 | 1.00 | 4.18 | 2.23 | 0 | 3.0 | 4.0 | 5.0 | 14.0 | ▃▇▂▁▁ |

```
table(Data_PSQI$Hours_Sleeppernight)
##
##                5              5.5                6              6.5
##                2                1                4                5
##             6.75 6.83333333333333                7             7.25
##                1                1               27                2
##              7.5 7.66666666666667             7.75                8
##               18                1                3               26
##              8.5                9              9.5
##                9                1                1
```

## Chronotype

The mean and standard deviation differences in descriptives between the chronotypes was assessed. Moreover, anova’s were performed to see if there were any significant differences in these descriptives between chronotypes. The Chronotype files for every outcome were also pivoted to perform linear mixed models.

### Differences in descriptives between chronotypes

Data_Sleep_IND_Chrono$Chronotype <- as.factor(Data_Sleep_IND_Chrono$Chronotype)

ChronoSexTable <- table(Data_Sleep_IND_Chrono$Chronotype, Data_Sleep_IND_Chrono$Geslacht) #sex

view(ChronoSexTable)

chisq.test(ChronoSexTable)

```
##
##  Pearson's Chi-squared test
##
## data:  ChronoSexTable
## X-squared = 2.58, df = 4, p-value = 0.6304
aggregate(Leeftijd ~ Chronotype, data = Data_Sleep_IND_Chrono, FUN = mean) #age
##   Chronotype Leeftijd
## 1          1 37.27273
## 2          2 30.64706
## 3          3 31.60714
## 4          4 31.30769
## 5          5 31.30000
aggregate(Leeftijd ~ Chronotype, data = Data_Sleep_IND_Chrono, FUN = sd)
##   Chronotype Leeftijd
## 1          1 8.361942
## 2          2 6.981593
## 3          3 8.473660
## 4          4 9.792724
## 5          5 8.442374
anova_Chronotype_Age <- aov(Leeftijd ~ Chronotype, data = Data_Sleep_IND_Chrono)
```

summary(anova_Chronotype_Age)

```
##             Df Sum Sq Mean Sq F value Pr(>F)
## Chronotype   4    628  157.07    2.23 0.0715 .
## Residuals   95   6692   70.44
## ---
## Signif. codes:  0 '***' 0.001 '**' 0.01 '*' 0.05 '.' 0.1 ' ' 1
eta_squared(anova_Chronotype_Age, partial = TRUE)
## For one-way between subjects designs, partial eta squared is equivalent
##   to eta squared. Returning eta squared.
## # Effect Size for ANOVA
##
## Parameter  | Eta2 |       95% CI
## --------------------------------
## Chronotype | 0.09 | [0.00, 1.00]
##
## - One-sided CIs: upper bound fixed at [1.00].
aggregate(BMI ~ Chronotype, data = Data_Sleep_IND_Chrono, FUN = mean) #BMI
##   Chronotype      BMI
## 1          1 22.59909
## 2          2 23.97706
## 3          3 22.86321
## 4          4 21.77385
## 5          5 22.86300
aggregate(BMI ~ Chronotype, data = Data_Sleep_IND_Chrono, FUN = sd)
##   Chronotype      BMI
## 1          1 2.173198
## 2          2 2.540612
## 3          3 2.549333
## 4          4 3.331182
## 5          5 2.597045
anova_Chronotype_BMI <- aov(BMI ~ Chronotype, data = Data_Sleep_IND_Chrono)
```

summary(anova_Chronotype_BMI)

```
##             Df Sum Sq Mean Sq F value Pr(>F)
## Chronotype   4   38.0   9.511   1.413  0.235
## Residuals   95  639.2   6.729
aggregate(`Vet%(Dexa)` ~ Chronotype, data = Data_Sleep_IND_Chrono, FUN = mean) #fat%
##   Chronotype Vet%(Dexa)
## 1          1   20.04545
## 2          2   24.69412
## 3          3   21.40000
## 4          4   18.36154
## 5          5   18.14000
aggregate(`Vet%(Dexa)` ~ Chronotype, data = Data_Sleep_IND_Chrono, FUN = sd)
##   Chronotype Vet%(Dexa)
## 1          1   6.416179
## 2          2   8.400035
## 3          3   8.277278
## 4          4   8.645667
## 5          5   6.428342
kruskal.test(`Vet%(Dexa)` ~ Chronotype, data = Data_Sleep_IND_Chrono)
##
##  Kruskal-Wallis rank sum test
##
## data:  Vet%(Dexa) by Chronotype
## Kruskal-Wallis chi-squared = 7.2764, df = 4, p-value = 0.122
aggregate(rVO2Max ~ Chronotype, data = Data_Sleep_IND_Chrono, FUN = mean) #VO2Max
##   Chronotype  rVO2Max
## 1          1 46.77273
## 2          2 43.29412
## 3          3 50.03571
## 4          4 46.38462
## 5          5 51.25000
aggregate(rVO2Max ~ Chronotype, data = Data_Sleep_IND_Chrono, FUN = sd)
##   Chronotype   rVO2Max
## 1          1  8.076608
## 2          2  9.190788
## 3          3  9.942360
## 4          4 10.516166
## 5          5  8.484506
anova_Chronotype_rVO2Max <- aov(rVO2Max ~ Chronotype, data = Data_Sleep_IND_Chrono)
```

summary(anova_Chronotype_rVO2Max)

```
##             Df Sum Sq Mean Sq F value Pr(>F)
## Chronotype   4    770  192.61   2.263  0.068 .
## Residuals   95   8085   85.11
## ---
## Signif. codes:  0 '***' 0.001 '**' 0.01 '*' 0.05 '.' 0.1 ' ' 1
aggregate(CCQ ~ Chronotype, data = Data_Sleep_IND_Chrono, FUN = mean) #CCQ
##   Chronotype       CCQ
## 1          1 1844.7247
## 2          2 1609.8320
## 3          3 1601.9633
## 4          4  895.2009
## 5          5 1120.9660
aggregate(CCQ ~ Chronotype, data = Data_Sleep_IND_Chrono, FUN = sd)
##   Chronotype       CCQ
## 1          1 1545.7236
## 2          2 1667.6469
## 3          3 1488.5208
## 4          4  852.6668
## 5          5 1142.7320
kruskal.test(CCQ ~ Chronotype, data = Data_Sleep_IND_Chrono)
##
##  Kruskal-Wallis rank sum test
##
## data:  CCQ by Chronotype
## Kruskal-Wallis chi-squared = 4.881, df = 4, p-value = 0.2997
aggregate(Sleep_duration ~ Chronotype, data = Data_Sleep_IND_Chrono, FUN = mean) #Sleep duration
##   Chronotype Sleep_duration
## 1          1       7.409091
## 2          2       7.186275
## 3          3       7.386905
## 4          4       7.615385
## 5          5       7.650000
aggregate(Sleep_duration ~ Chronotype, data = Data_Sleep_IND_Chrono, FUN = sd)
##   Chronotype Sleep_duration
## 1          1      0.5750024
## 2          2      0.8875731
## 3          3      0.7748670
## 4          4      0.7116251
## 5          5      0.8936913
anova_Chronotype_SD <- aov(Sleep_duration ~ Chronotype, data = Data_Sleep_IND_Chrono)
```

summary(anova_Chronotype_SD)

```
##             Df Sum Sq Mean Sq F value Pr(>F)
## Chronotype   4   2.48  0.6191   1.032  0.395
## Residuals   95  57.01  0.6001
eta_squared(anova_Chronotype_SD, partial = TRUE)
## For one-way between subjects designs, partial eta squared is equivalent
##   to eta squared. Returning eta squared.
## # Effect Size for ANOVA
##
## Parameter  | Eta2 |       95% CI
## --------------------------------
## Chronotype | 0.04 | [0.00, 1.00]
##
## - One-sided CIs: upper bound fixed at [1.00].
aggregate(PSQI ~ Chronotype, data = Data_Sleep_IND_Chrono, FUN = mean) #PSQI
##   Chronotype     PSQI
## 1          1 3.909091
## 2          2 5.058824
## 3          3 4.250000
## 4          4 3.923077
## 5          5 3.500000
anova_Chronotype_PSQI <- aov(PSQI ~ Chronotype, data = Data_Sleep_IND_Chrono)
```

summary(anova_Chronotype_PSQI)

```
##             Df Sum Sq Mean Sq F value Pr(>F)
## Chronotype   4   24.6   6.157   1.272  0.287
## Residuals   95  459.9   4.841
eta_squared(anova_Chronotype_PSQI, partial = TRUE)
## For one-way between subjects designs, partial eta squared is equivalent
##   to eta squared. Returning eta squared.
## # Effect Size for ANOVA
##
## Parameter  | Eta2 |       95% CI
## --------------------------------
## Chronotype | 0.05 | [0.00, 1.00]
##
## - One-sided CIs: upper bound fixed at [1.00].
```

The following figure shows the distribution of number of participants for every chronotype.

```
## Warning: Removed 1 row containing non-finite outside the scale range (`stat_count()`).
## Removed 1 row containing non-finite outside the scale range (`stat_count()`).
```

### Subjective feeling of mental fatigue

Data_Chronotype_MVAS_Pivot <- Data_Chronotype_MVASMC %>%

pivot_longer(

cols = starts_with("MVAS"),

names_to = c("condition", "time"),

names_pattern = "MVAS_(.*)_(.*)",

values_to = "value"

) %>%

mutate(time = case_when(

time == "2" ~ "1",

time == "5" ~ "2",

TRUE ~ time

))

### Go Stimuli

Data_Chronotype_zscore_transformed <- Data_Chronotype_Go_zscore %>%

pivot_longer(

cols = starts_with("Go_"),

names_to = c("measure", "conditie", "tijd"),

names_pattern = "Go_(RT|ACC)_([^_]+)_([^_]+)",

values_to = "value"

) %>%

pivot_wider(

names_from = measure,

values_from = value

) %>%

mutate(

RT_z = (scale(-RT)),

ACC_z = (scale(ACC))

) %>%

mutate(

z_score = (RT_z + ACC_z) / 2

)

### Time trial

Data_Chronotype_TT_Pivot <- Data_Chronotype_TT %>%

pivot_longer(

cols = c(TT_MF_Distance, TT_CON_Distance),

names_to = "condition",

values_to = "distance"

) %>%

mutate(

condition = ifelse(condition == "TT_MF_Distance", "MF", "CON")

)

### Rating of perceived exertion

Data_Chronotype_RPE_Pivot <- Data_Chronotype_RPE %>%

pivot_longer(

cols = starts_with("RPE"),

names_to = c("time", "condition"),

names_pattern = "RPE_(.*)_(.*)",

values_to = "value"

)

### Karolinska sleepiness scale

Data_Chronotype_KSS_Pivot <- Data_Chronotype_KSS %>%

pivot_longer(

cols = starts_with("KSS"),

names_to = c("time", "condition"),

names_pattern = "KSS_(.*)_(.*)",

values_to = "value"

)

## PSQI

The same comparisons and updating of data files was performed for the specific PSQI groups.

### Differences in descriptives between PSQI groups

Data_Sleep_IND_PSQI$PSQI_group <- as.factor(Data_Sleep_IND_PSQI$PSQI_group)

PSQISexTable <- table(Data_Sleep_IND_PSQI$PSQI_group, Data_Sleep_IND_PSQI$Geslacht) #sex

PSQISexTable

```
##
##      M  V
##   1 12  9
##   2 12  9
chisq.test(PSQISexTable)
##
##  Pearson's Chi-squared test
##
## data:  PSQISexTable
## X-squared = 0, df = 1, p-value = 1
aggregate(Leeftijd ~ PSQI_group, data = Data_Sleep_IND_PSQI, FUN = mean) #age
##   PSQI_group Leeftijd
## 1          1 34.28571
## 2          2 30.42857
aggregate(Leeftijd ~ PSQI_group, data = Data_Sleep_IND_PSQI, FUN = sd)
##   PSQI_group Leeftijd
## 1          1 8.775778
## 2          2 5.852960
t.test(Leeftijd ~ PSQI_group, data = Data_Sleep_IND_PSQI, var.equal = FALSE)
##
##  Welch Two Sample t-test
##
## data:  Leeftijd by PSQI_group
## t = 1.6757, df = 34.854, p-value = 0.1028
## alternative hypothesis: true difference in means between group 1 and group 2 is not equal to 0
## 95 percent confidence interval:
##  -0.8166181  8.5309039
## sample estimates:
## mean in group 1 mean in group 2
##        34.28571        30.42857
aggregate(BMI ~ PSQI_group, data = Data_Sleep_IND_PSQI, FUN = mean) #BMI
##   PSQI_group      BMI
## 1          1 23.54857
## 2          2 23.15810
aggregate(BMI ~ PSQI_group, data = Data_Sleep_IND_PSQI, FUN = sd)
##   PSQI_group      BMI
## 1          1 2.672804
## 2          2 2.601605
t.test(BMI ~ PSQI_group, data = Data_Sleep_IND_PSQI, var.equal = FALSE)
##
##  Welch Two Sample t-test
##
## data:  BMI by PSQI_group
## t = 0.47974, df = 39.971, p-value = 0.634
## alternative hypothesis: true difference in means between group 1 and group 2 is not equal to 0
## 95 percent confidence interval:
##  -1.254581  2.035534
## sample estimates:
## mean in group 1 mean in group 2
##        23.54857        23.15810
aggregate(`Vet%(Dexa)` ~ PSQI_group, data = Data_Sleep_IND_PSQI, FUN = mean) #fat%
##   PSQI_group Vet%(Dexa)
## 1          1   21.82857
## 2          2   19.98571
aggregate(`Vet%(Dexa)` ~ PSQI_group, data = Data_Sleep_IND_PSQI, FUN = sd)
##   PSQI_group Vet%(Dexa)
## 1          1   8.462987
## 2          2   7.712476
wilcox.test(`Vet%(Dexa)` ~ PSQI_group, data = Data_Sleep_IND_PSQI)
## Warning in wilcox.test.default(x = DATA[[1L]], y = DATA[[2L]], ...): cannot
## compute exact p-value with ties
##
##  Wilcoxon rank sum test with continuity correction
##
## data:  Vet%(Dexa) by PSQI_group
## W = 244.5, p-value = 0.5544
## alternative hypothesis: true location shift is not equal to 0
aggregate(rVO2Max ~ PSQI_group, data = Data_Sleep_IND_PSQI, FUN = mean) #VO2max
##   PSQI_group  rVO2Max
## 1          1 47.28571
## 2          2 48.85714
aggregate(rVO2Max ~ PSQI_group, data = Data_Sleep_IND_PSQI, FUN = sd)
##   PSQI_group   rVO2Max
## 1          1 10.469684
## 2          2  9.285934
t.test(rVO2Max ~ PSQI_group, data = Data_Sleep_IND_PSQI, var.equal = FALSE)
##
##  Welch Two Sample t-test
##
## data:  rVO2Max by PSQI_group
## t = -0.51458, df = 39.438, p-value = 0.6097
## alternative hypothesis: true difference in means between group 1 and group 2 is not equal to 0
## 95 percent confidence interval:
##  -7.746183  4.603326
## sample estimates:
## mean in group 1 mean in group 2
##        47.28571        48.85714
aggregate(CCQ ~ PSQI_group, data = Data_Sleep_IND_PSQI, FUN = mean) #CCQ
##   PSQI_group      CCQ
## 1          1 1831.023
## 2          2 1355.938
aggregate(CCQ ~ PSQI_group, data = Data_Sleep_IND_PSQI, FUN = sd)
##   PSQI_group      CCQ
## 1          1 1735.847
## 2          2 1454.359
wilcox.test(CCQ ~ PSQI_group, data = Data_Sleep_IND_PSQI)
## Warning in wilcox.test.default(x = DATA[[1L]], y = DATA[[2L]], ...): cannot
## compute exact p-value with ties
##
##  Wilcoxon rank sum test with continuity correction
##
## data:  CCQ by PSQI_group
## W = 262, p-value = 0.3023
## alternative hypothesis: true location shift is not equal to 0
aggregate(Sleep_duration ~ PSQI_group, data = Data_Sleep_IND_PSQI, FUN = mean) #sleep duration
##   PSQI_group Sleep_duration
## 1          1       7.920635
## 2          2       6.654762
aggregate(Sleep_duration ~ PSQI_group, data = Data_Sleep_IND_PSQI, FUN = sd)
##   PSQI_group Sleep_duration
## 1          1      0.6746790
## 2          2      0.8532152
t.test(Sleep_duration ~ PSQI_group, data = Data_Sleep_IND_PSQI, var.equal = FALSE)
##
##  Welch Two Sample t-test
##
## data:  Sleep_duration by PSQI_group
## t = 5.3331, df = 37.981, p-value = 4.678e-06
## alternative hypothesis: true difference in means between group 1 and group 2 is not equal to 0
## 95 percent confidence interval:
##  0.7853483 1.7463977
## sample estimates:
## mean in group 1 mean in group 2
##        7.920635        6.654762
PSQIChronoTable <- table(Data_Sleep_IND_PSQI$PSQI_group, Data_Sleep_IND_PSQI$Chronotype) #chronotype
```

PSQIChronoTable

```
##
##     1 2 3 4 5
##   1 4 1 7 1 8
##   2 3 6 8 1 2
chisq.test(PSQIChronoTable)
## Warning in chisq.test(PSQIChronoTable): Chi-squared approximation may be
## incorrect
##
##  Pearson's Chi-squared test
##
## data:  PSQIChronoTable
## X-squared = 7.3609, df = 4, p-value = 0.118
```

### Subjective feeling of mental fatigue

Data_PSQIgroups_MVAS_Pivot <- Data_PSQI_MVASMC %>%

pivot_longer(

cols = starts_with("MVAS"),

names_to = c("condition", "time"),

names_pattern = "MVAS_(.*)_(.*)",

values_to = "value"

) %>%

mutate(time = case_when(

time == "2" ~ "1",

time == "5" ~ "2",

TRUE ~ time

))

Data_PSQIcont_MVASMC <- Data_PSQI_MVASMC_cont %>%

pivot_longer(

cols = starts_with("MVAS"),

names_to = c("condition", "time"),

names_pattern = "MVAS_(.*)_(.*)",

values_to = "value"

) %>%

mutate(time = case_when(

time == "2" ~ "1",

time == "5" ~ "2",

TRUE ~ time

))

### Go stimuli

Data_PSQIgroups_zscore_transformed <- Data_PSQI_Go_zscore %>%

pivot_longer(

cols = starts_with("Go_"),

names_to = c("measure", "conditie", "tijd"),

names_pattern = "Go_(.*)_(.*)_(.*)",

values_to = "value"

) %>%

pivot_wider(

names_from = measure,

values_from = value

) %>%

mutate(

RT_z = scale(-RT),

ACC_z = scale(ACC)

) %>%

mutate(

z_score = (RT_z + ACC_z) / 2

)

Data_PSQIcont_zscore_transformed <- Data_PSQI_Go_zscore_cont %>%

pivot_longer(

cols = starts_with("Go_"),

names_to = c("measure", "conditie", "tijd"),

names_pattern = "Go_(.*)_(.*)_(.*)",

values_to = "value"

) %>%

pivot_wider(

names_from = measure,

values_from = value

) %>%

mutate(

RT_z = scale(-RT),

ACC_z = scale(ACC)

) %>%

mutate(

z_score = (RT_z + ACC_z) / 2

)

### Time trial

Data_PSQIgroups_TT_Pivot <- Data_PSQI_TT %>%

pivot_longer(

cols = c(TT_MF_Distance, TT_CON_Distance),

names_to = "condition",

values_to = "distance"

) %>%

mutate(

condition = ifelse(condition == "TT_MF_Distance", "MF", "CON")

)

Data_PSQIcont_TT_Pivot <- Data_PSQI_TT_cont %>%

pivot_longer(

cols = c(TT_MF_Distance, TT_CON_Distance),

names_to = "condition",

values_to = "distance"

) %>%

mutate(

condition = ifelse(condition == "TT_MF_Distance", "MF", "CON")

)

### Rating of perceieved exertion

Data_PSQIgroups_RPE_Pivot <- Data_PSQI_RPE %>%

pivot_longer(

cols = starts_with("RPE"),

names_to = c("time", "condition"),

names_pattern = "RPE_(.*)_(.*)",

values_to = "value"

)

Data_PSQIcont_RPE_Pivot <- Data_PSQI_RPE_cont %>%

pivot_longer(

cols = starts_with("RPE"),

names_to = c("time", "condition"),

names_pattern = "RPE_(.*)_(.*)",

values_to = "value"

)

### Karolinska sleepiness scale

Data_PSQIgroups_KSS_Pivot <- Data_PSQI_KSS %>% pivot_longer(

cols = starts_with("KSS"),

names_to = c("time", "condition"),

names_pattern = "KSS_(.*)_(.*)",

values_to = "value"

)

Data_PSQIcont_KSS_Pivot <- Data_PSQI_KSS_cont %>% pivot_longer(

cols = starts_with("KSS"),

names_to = c("time", "condition"),

names_pattern = "KSS_(.*)_(.*)",

values_to = "value"

)

#Analyses

shapiro_test <- function(column) {

if (is.numeric(column) && length(column) > 3) {

test_result <- shapiro.test(column)

return(test_result$p.value)

} else {

return(NA)

}

}

detect_outliers_z <- function(column, proefpersonen) {

if(!is.numeric(column)) {

return(NULL)

}

valid_indices <- !is.na(column) & !is.na(proefpersonen)

column_clean <- column[valid_indices]

proefpersonen_clean <- proefpersonen[valid_indices]

if (length(unique(column_clean)) == 1) {

return(NULL)

}

z_scores <- scale(column_clean)

outlier_indices <- which(abs(z_scores) >3)

if (length(outlier_indices) > 0) {

outlier_proefpersonen <- proefpersonen_clean[outlier_indices]

return(outlier_proefpersonen)

} else {

return(NULL)

}

}

## Difference in mental fatigue variables between chronotype groups

### Subjective feeling of mental fatigue

#### Outliers

outliersMVASChronotype<- lapply(Data_Chronotype_MVASMC[, -1], function(col) {

detect_outliers_z(col, Data_Chronotype_MVASMC[, 1])

})

outliersMVASChronotype

```
## $Leeftijd
## NULL
##
## $Chronotype
## NULL
##
## $CCQ
## [1] "MF_INDIFF_V1_020" "MF_INDIFF_V1_238"
##
## $MVAS_MF_2
## [1] "MF_INDIFF_V1_016" "MF_INDIFF_V1_143"
##
## $MVAS_MF_5
## NULL
##
## $MVAS_CON_2
## NULL
##
## $MVAS_CON_5
## [1] "MF_INDIFF_V1_278"
##
## $CCQ_z
## [1] "MF_INDIFF_V1_020" "MF_INDIFF_V1_238"
```

#### Normality

shapiro_results_Chr_MVASMC <- lapply(Data_Chronotype_MVASMC, shapiro_test)

shapiro_results_Chr_MVASMC

```
## $PP_number
## [1] NA
##
## $Leeftijd
## [1] 0.0008358021
##
## $Chronotype
## [1] 2.976966e-07
##
## $CCQ
## [1] 1.820455e-08
##
## $MVAS_MF_2
## [1] 2.558679e-05
##
## $MVAS_MF_5
## [1] 0.001304851
##
## $MVAS_CON_2
## [1] 5.476029e-07
##
## $MVAS_CON_5
## [1] 0.0002767667
##
## $CCQ_z
## [1] 1.820455e-08
```

Results of the MVAS were not normally distributed. We therefore chose to perform a square root transformation, as with the original data analysis. This lead to a normalized distribution.

sqrtMVAS_Chronotype <- Data_Chronotype_MVASMC

sqrtMVAS_Chronotype[, -c(1:4, 9)] <- sqrt(Data_Chronotype_MVASMC[, -c(1:4, 9)])

sqrtMVAS_Chronotype_Pivot <- sqrtMVAS_Chronotype %>%

pivot_longer(

cols = starts_with("MVAS"),

names_to = c("condition", "time"),

names_pattern = "MVAS_(.*)_(.*)",

values_to = "value"

) %>%

mutate(time = case_when(

time == "2" ~ "1",

time == "5" ~ "2",

TRUE ~ time

))

shapiro_results_Chr_sqrtMVASMC <- lapply(sqrtMVAS_Chronotype, shapiro_test)

shapiro_results_Chr_sqrtMVASMC

```
## $PP_number
## [1] NA
##
## $Leeftijd
## [1] 0.0008358021
##
## $Chronotype
## [1] 2.976966e-07
##
## $CCQ
## [1] 1.820455e-08
##
## $MVAS_MF_2
## [1] 0.8724278
##
## $MVAS_MF_5
## [1] 2.443744e-05
##
## $MVAS_CON_2
## [1] 0.04052898
##
## $MVAS_CON_5
## [1] 0.08988771
##
## $CCQ_z
## [1] 1.820455e-08
```

#### Models

sqrtMVAS_Chronotype_Pivot$Chronotype <- as.factor(sqrtMVAS_Chronotype_Pivot$Chronotype)

sqrtMVAS_Chronotype_Pivot$condition <- as.factor(sqrtMVAS_Chronotype_Pivot$condition)

sqrtMVAS_Chronotype_Pivot$time <- as.factor(sqrtMVAS_Chronotype_Pivot$time)

model_Chronotype_MVASMC <- lmer(value ~ condition*time*Chronotype + CCQ_z[, 1] + Leeftijd + (1 | PP_number), data = sqrtMVAS_Chronotype_Pivot)

anovaMVASMC <- anova(model_Chronotype_MVASMC)

summary(model_Chronotype_MVASMC)

```
## Linear mixed model fit by REML. t-tests use Satterthwaite's method [
## lmerModLmerTest]
## Formula: value ~ condition * time * Chronotype + CCQ_z[, 1] + Leeftijd +
##     (1 | PP_number)
##    Data: sqrtMVAS_Chronotype_Pivot
##
## REML criterion at convergence: 1488
##
## Scaled residuals:
##     Min      1Q  Median      3Q     Max
## -2.9758 -0.5372  0.0305  0.5252  3.0904
##
## Random effects:
##  Groups    Name        Variance Std.Dev.
##  PP_number (Intercept) 0.7699   0.8774
##  Residual              1.9469   1.3953
## Number of obs: 400, groups:  PP_number, 100
##
## Fixed effects:
##                                Estimate Std. Error        df t value Pr(>|t|)
## (Intercept)                     3.74925    0.63393 131.75234   5.914 2.71e-08
## conditionMF                     0.06445    0.42070 285.00000   0.153   0.8783
## time2                           0.08144    0.42070 285.00000   0.194   0.8466
## Chronotype2                     0.15829    0.54002 293.07434   0.293   0.7696
## Chronotype3                     0.73778    0.47598 293.64492   1.550   0.1222
## Chronotype4                     0.40027    0.58497 293.11071   0.684   0.4944
## Chronotype5                    -0.93535    0.51701 292.67767  -1.809   0.0715
## CCQ_z[, 1]                      0.01760    0.12167  93.00000   0.145   0.8853
## Leeftijd                        0.02290    0.01440  93.00000   1.590   0.1151
## conditionMF:time2               3.40608    0.59496 285.00000   5.725 2.62e-08
## conditionMF:Chronotype2        -0.25750    0.63720 285.00000  -0.404   0.6864
## conditionMF:Chronotype3        -0.56362    0.56218 285.00000  -1.003   0.3169
## conditionMF:Chronotype4        -0.11857    0.69029 285.00000  -0.172   0.8637
## conditionMF:Chronotype5         0.17432    0.60965 285.00000   0.286   0.7751
## time2:Chronotype2              -0.68378    0.63720 285.00000  -1.073   0.2841
## time2:Chronotype3               0.90483    0.56218 285.00000   1.609   0.1086
## time2:Chronotype4              -0.07371    0.69029 285.00000  -0.107   0.9150
## time2:Chronotype5               0.36163    0.60965 285.00000   0.593   0.5535
## conditionMF:time2:Chronotype2   1.01136    0.90114 285.00000   1.122   0.2627
## conditionMF:time2:Chronotype3  -0.55859    0.79504 285.00000  -0.703   0.4829
## conditionMF:time2:Chronotype4  -0.09717    0.97622 285.00000  -0.100   0.9208
## conditionMF:time2:Chronotype5   0.30223    0.86217 285.00000   0.351   0.7262
##
## (Intercept)                   ***
## conditionMF
## time2
## Chronotype2
## Chronotype3
## Chronotype4
## Chronotype5                   .
## CCQ_z[, 1]
## Leeftijd
## conditionMF:time2             ***
## conditionMF:Chronotype2
## conditionMF:Chronotype3
## conditionMF:Chronotype4
## conditionMF:Chronotype5
## time2:Chronotype2
## time2:Chronotype3
## time2:Chronotype4
## time2:Chronotype5
## conditionMF:time2:Chronotype2
## conditionMF:time2:Chronotype3
## conditionMF:time2:Chronotype4
## conditionMF:time2:Chronotype5
## ---
## Signif. codes:  0 '***' 0.001 '**' 0.01 '*' 0.05 '.' 0.1 ' ' 1
##
## Correlation matrix not shown by default, as p = 22 > 12.
## Use print(x, correlation=TRUE)  or
##     vcov(x)        if you need it
print(anovaMVASMC)
## Type III Analysis of Variance Table with Satterthwaite's method
##                           Sum Sq Mean Sq NumDF DenDF  F value    Pr(>F)
## condition                 264.42  264.42     1   285 135.8174 < 2.2e-16 ***
## time                      356.90  356.90     1   285 183.3236 < 2.2e-16 ***
## Chronotype                 35.56    8.89     4    93   4.5660  0.002066 **
## CCQ_z[, 1]                  0.04    0.04     1    93   0.0209  0.885301
## Leeftijd                    4.92    4.92     1    93   2.5295  0.115128
## condition:time            293.05  293.05     1   285 150.5225 < 2.2e-16 ***
## condition:Chronotype       21.16    5.29     4   285   2.7170  0.030110 *
## time:Chronotype            11.59    2.90     4   285   1.4880  0.205873
## condition:time:Chronotype   6.92    1.73     4   285   0.8884  0.471222
## ---
## Signif. codes:  0 '***' 0.001 '**' 0.01 '*' 0.05 '.' 0.1 ' ' 1
```

The model showed no influence of age or caffeine consumption. An additional model was therefore constructed.

model_Chronotype_MVASMC_2 <- lmer(value ~ condition*time*Chronotype + (1 | PP_number), data = sqrtMVAS_Chronotype_Pivot)

anovaMVASMC_2 <- anova(model_Chronotype_MVASMC_2)

effectsize_Chronotype_MVAS <- eta_squared(model_Chronotype_MVASMC_2, partial = TRUE)

summary(model_Chronotype_MVASMC_2)

```
## Linear mixed model fit by REML. t-tests use Satterthwaite's method [
## lmerModLmerTest]
## Formula: value ~ condition * time * Chronotype + (1 | PP_number)
##    Data: sqrtMVAS_Chronotype_Pivot
##
## REML criterion at convergence: 1481.9
##
## Scaled residuals:
##     Min      1Q  Median      3Q     Max
## -3.0476 -0.5414  0.0497  0.5233  3.1671
##
## Random effects:
##  Groups    Name        Variance Std.Dev.
##  PP_number (Intercept) 0.7827   0.8847
##  Residual              1.9469   1.3953
## Number of obs: 400, groups:  PP_number, 100
##
## Fixed effects:
##                                Estimate Std. Error        df t value Pr(>|t|)
## (Intercept)                     4.60741    0.35224 304.80943  13.080  < 2e-16
## conditionMF                     0.06445    0.42070 285.00000   0.153    0.878
## time2                           0.08144    0.42070 285.00000   0.194    0.847
## Chronotype2                     0.00365    0.53351 304.80943   0.007    0.995
## Chronotype3                     0.60503    0.47070 304.80943   1.285    0.200
## Chronotype4                     0.25189    0.57796 304.80943   0.436    0.663
## Chronotype5                    -1.08110    0.51044 304.80943  -2.118    0.035
## conditionMF:time2               3.40608    0.59496 285.00000   5.725 2.62e-08
## conditionMF:Chronotype2        -0.25750    0.63720 285.00000  -0.404    0.686
## conditionMF:Chronotype3        -0.56362    0.56218 285.00000  -1.003    0.317
## conditionMF:Chronotype4        -0.11857    0.69029 285.00000  -0.172    0.864
## conditionMF:Chronotype5         0.17432    0.60965 285.00000   0.286    0.775
## time2:Chronotype2              -0.68378    0.63720 285.00000  -1.073    0.284
## time2:Chronotype3               0.90483    0.56218 285.00000   1.609    0.109
## time2:Chronotype4              -0.07371    0.69029 285.00000  -0.107    0.915
## time2:Chronotype5               0.36163    0.60965 285.00000   0.593    0.554
## conditionMF:time2:Chronotype2   1.01136    0.90114 285.00000   1.122    0.263
## conditionMF:time2:Chronotype3  -0.55858    0.79505 285.00000  -0.703    0.483
## conditionMF:time2:Chronotype4  -0.09717    0.97622 285.00000  -0.100    0.921
## conditionMF:time2:Chronotype5   0.30223    0.86218 285.00000   0.351    0.726
##
## (Intercept)                   ***
## conditionMF
## time2
## Chronotype2
## Chronotype3
## Chronotype4
## Chronotype5                   *
## conditionMF:time2             ***
## conditionMF:Chronotype2
## conditionMF:Chronotype3
## conditionMF:Chronotype4
## conditionMF:Chronotype5
## time2:Chronotype2
## time2:Chronotype3
## time2:Chronotype4
## time2:Chronotype5
## conditionMF:time2:Chronotype2
## conditionMF:time2:Chronotype3
## conditionMF:time2:Chronotype4
## conditionMF:time2:Chronotype5
## ---
## Signif. codes:  0 '***' 0.001 '**' 0.01 '*' 0.05 '.' 0.1 ' ' 1
##
## Correlation matrix not shown by default, as p = 20 > 12.
## Use print(x, correlation=TRUE)  or
##     vcov(x)        if you need it
print(anovaMVASMC_2)
## Type III Analysis of Variance Table with Satterthwaite's method
##                           Sum Sq Mean Sq NumDF DenDF  F value    Pr(>F)
## condition                 264.42  264.42     1   285 135.8174 < 2.2e-16 ***
## time                      356.90  356.90     1   285 183.3236 < 2.2e-16 ***
## Chronotype                 35.58    8.89     4    95   4.5685  0.002034 **
## condition:time            293.05  293.05     1   285 150.5225 < 2.2e-16 ***
## condition:Chronotype       21.16    5.29     4   285   2.7170  0.030110 *
## time:Chronotype            11.59    2.90     4   285   1.4880  0.205873
## condition:time:Chronotype   6.92    1.73     4   285   0.8884  0.471222
## ---
## Signif. codes:  0 '***' 0.001 '**' 0.01 '*' 0.05 '.' 0.1 ' ' 1
print(effectsize_Chronotype_MVAS)
## # Effect Size for ANOVA (Type III)
##
## Parameter                 | Eta2 (partial) |       95% CI
## ---------------------------------------------------------
## condition                 |           0.32 | [0.25, 1.00]
## time                      |           0.39 | [0.32, 1.00]
## Chronotype                |           0.16 | [0.04, 1.00]
## condition:time            |           0.35 | [0.28, 1.00]
## condition:Chronotype      |           0.04 | [0.00, 1.00]
## time:Chronotype           |           0.02 | [0.00, 1.00]
## condition:time:Chronotype |           0.01 | [0.00, 1.00]
##
## - One-sided CIs: upper bound fixed at [1.00].
confint(model_Chronotype_MVASMC_2)
## Computing profile confidence intervals ...
##                                    2.5 %     97.5 %
## .sig01                         0.6714226  1.0750068
## .sigma                         1.2580018  1.4764920
## (Intercept)                    3.9324913  5.2823275
## conditionMF                   -0.7418028  0.8707070
## time2                         -0.7248140  0.8876958
## Chronotype2                   -1.0186046  1.0259038
## Chronotype3                   -0.2968655  1.5069290
## Chronotype4                   -0.8555356  1.3593098
## Chronotype5                   -2.0591544 -0.1030559
## conditionMF:time2              2.2658631  4.5462964
## conditionMF:Chronotype2       -1.4786773  0.9636857
## conditionMF:Chronotype3       -1.6410256  0.5137813
## conditionMF:Chronotype4       -1.4414892  1.2043578
## conditionMF:Chronotype5       -0.9940574  1.3426913
## time2:Chronotype2             -1.9049582  0.5374049
## time2:Chronotype3             -0.1725751  1.9822318
## time2:Chronotype4             -1.3966292  1.2492178
## time2:Chronotype5             -0.8067450  1.5300037
## conditionMF:time2:Chronotype2 -0.7156497  2.7383732
## conditionMF:time2:Chronotype3 -2.0822636  0.9650935
## conditionMF:time2:Chronotype4 -1.9680652  1.7737274
## conditionMF:time2:Chronotype5 -1.3500993  1.9545625
```

#### Post Hoc

```
emmeans(model_Chronotype_MVASMC_2, ~ condition | Chronotype)
## NOTE: Results may be misleading due to involvement in interactions
## Chronotype = 1:
##  condition emmean    SE  df lower.CL upper.CL
##  CON         4.65 0.283 173     4.09     5.21
##  MF          6.42 0.283 173     5.86     6.97
##
## Chronotype = 2:
##  condition emmean    SE  df lower.CL upper.CL
##  CON         4.31 0.321 173     3.68     4.94
##  MF          6.33 0.321 173     5.69     6.96
##
## Chronotype = 3:
##  condition emmean    SE  df lower.CL upper.CL
##  CON         5.71 0.250 173     5.21     6.20
##  MF          6.63 0.250 173     6.14     7.12
##
## Chronotype = 4:
##  condition emmean    SE  df lower.CL upper.CL
##  CON         4.86 0.368 173     4.14     5.59
##  MF          6.46 0.368 173     5.74     7.19
##
## Chronotype = 5:
##  condition emmean    SE  df lower.CL upper.CL
##  CON         3.75 0.296 173     3.16     4.33
##  MF          5.84 0.296 173     5.26     6.43
##
## Results are averaged over the levels of: time
## Degrees-of-freedom method: kenward-roger
## Confidence level used: 0.95
pairs(emmeans(model_Chronotype_MVASMC_2, ~ condition | Chronotype))
## NOTE: Results may be misleading due to involvement in interactions
## Chronotype = 1:
##  contrast estimate    SE  df t.ratio p.value
##  CON - MF   -1.767 0.297 285  -5.942 <0.0001
##
## Chronotype = 2:
##  contrast estimate    SE  df t.ratio p.value
##  CON - MF   -2.016 0.338 285  -5.956 <0.0001
##
## Chronotype = 3:
##  contrast estimate    SE  df t.ratio p.value
##  CON - MF   -0.925 0.264 285  -3.506  0.0005
##
## Chronotype = 4:
##  contrast estimate    SE  df t.ratio p.value
##  CON - MF   -1.600 0.387 285  -4.135 <0.0001
##
## Chronotype = 5:
##  contrast estimate    SE  df t.ratio p.value
##  CON - MF   -2.093 0.312 285  -6.708 <0.0001
##
## Results are averaged over the levels of: time
## Degrees-of-freedom method: kenward-roger
```

### Go stimuli

#### Model

Data_Chronotype_zscore_transformed$Chronotype <- as.factor(Data_Chronotype_zscore_transformed$Chronotype)

Data_Chronotype_zscore_transformed$conditie <- as.factor(Data_Chronotype_zscore_transformed$conditie)

Data_Chronotype_zscore_transformed$tijd <- as.factor(Data_Chronotype_zscore_transformed$tijd)

model_Chronotype_zscore <- lmer(z_score[, 1] ~ conditie*tijd*Chronotype + CCQ_z[, 1] + Leeftijd + (1 | PP_number), data = Data_Chronotype_zscore_transformed)

summary(model_Chronotype_zscore)

```
## Linear mixed model fit by REML. t-tests use Satterthwaite's method [
## lmerModLmerTest]
## Formula: z_score[, 1] ~ conditie * tijd * Chronotype + CCQ_z[, 1] + Leeftijd +
##     (1 | PP_number)
##    Data: Data_Chronotype_zscore_transformed
##
## REML criterion at convergence: 678.8
##
## Scaled residuals:
##     Min      1Q  Median      3Q     Max
## -3.4004 -0.5014  0.0743  0.5906  2.6536
##
## Random effects:
##  Groups    Name        Variance Std.Dev.
##  PP_number (Intercept) 0.2402   0.4901
##  Residual              0.1844   0.4295
## Number of obs: 400, groups:  PP_number, 100
##
## Fixed effects:
##                                  Estimate Std. Error         df t value
## (Intercept)                      0.464561   0.287621 108.689277   1.615
## conditieMF                      -0.345260   0.129488 285.000000  -2.666
## tijdPre                          0.103253   0.129488 285.000000   0.797
## Chronotype2                      0.147176   0.214884 184.284593   0.685
## Chronotype3                      0.230568   0.189322 184.655281   1.218
## Chronotype4                      0.186014   0.232766 184.308175   0.799
## Chronotype5                      0.220792   0.205790 184.027808   1.073
## CCQ_z[, 1]                      -0.055466   0.058077  93.000000  -0.955
## Leeftijd                        -0.016744   0.006872  93.000000  -2.437
## conditieMF:tijdPre               0.530821   0.183123 285.000000   2.899
## conditieMF:Chronotype2          -0.219503   0.196126 285.000000  -1.119
## conditieMF:Chronotype3          -0.060863   0.173035 285.000000  -0.352
## conditieMF:Chronotype4          -0.342682   0.212466 285.000000  -1.613
## conditieMF:Chronotype5           0.069926   0.187645 285.000000   0.373
## tijdPre:Chronotype2              0.059451   0.196126 285.000000   0.303
## tijdPre:Chronotype3             -0.050912   0.173035 285.000000  -0.294
## tijdPre:Chronotype4             -0.054581   0.212466 285.000000  -0.257
## tijdPre:Chronotype5             -0.048683   0.187645 285.000000  -0.259
## conditieMF:tijdPre:Chronotype2  -0.017173   0.277364 285.000000  -0.062
## conditieMF:tijdPre:Chronotype3  -0.219855   0.244708 285.000000  -0.898
## conditieMF:tijdPre:Chronotype4  -0.042125   0.300473 285.000000  -0.140
## conditieMF:tijdPre:Chronotype5  -0.360902   0.265370 285.000000  -1.360
##                                Pr(>|t|)
## (Intercept)                     0.10917
## conditieMF                      0.00811 **
## tijdPre                         0.42589
## Chronotype2                     0.49426
## Chronotype3                     0.22483
## Chronotype4                     0.42524
## Chronotype5                     0.28472
## CCQ_z[, 1]                      0.34204
## Leeftijd                        0.01673 *
## conditieMF:tijdPre              0.00404 **
## conditieMF:Chronotype2          0.26400
## conditieMF:Chronotype3          0.72529
## conditieMF:Chronotype4          0.10788
## conditieMF:Chronotype5          0.70968
## tijdPre:Chronotype2             0.76202
## tijdPre:Chronotype3             0.76880
## tijdPre:Chronotype4             0.79745
## tijdPre:Chronotype5             0.79548
## conditieMF:tijdPre:Chronotype2  0.95068
## conditieMF:tijdPre:Chronotype3  0.36971
## conditieMF:tijdPre:Chronotype4  0.88860
## conditieMF:tijdPre:Chronotype5  0.17491
## ---
## Signif. codes:  0 '***' 0.001 '**' 0.01 '*' 0.05 '.' 0.1 ' ' 1
##
## Correlation matrix not shown by default, as p = 22 > 12.
## Use print(x, correlation=TRUE)  or
##     vcov(x)        if you need it
```

The model showed no influence of caffeine consumption. An additional model was therefore constructed.

model_Chronotype_zscore_2 <- lmer(z_score[, 1] ~ conditie*tijd*Chronotype + Leeftijd + (1 | PP_number), data = Data_Chronotype_zscore_transformed)

anova_zscore_chronotype <- anova(model_Chronotype_zscore_2 )

effectsize_Chronotype_zscore <- eta_squared(model_Chronotype_zscore_2, partial = TRUE)

summary(model_Chronotype_zscore_2)

```
## Linear mixed model fit by REML. t-tests use Satterthwaite's method [
## lmerModLmerTest]
## Formula: z_score[, 1] ~ conditie * tijd * Chronotype + Leeftijd + (1 |
##     PP_number)
##    Data: Data_Chronotype_zscore_transformed
##
## REML criterion at convergence: 675.9
##
## Scaled residuals:
##     Min      1Q  Median      3Q     Max
## -3.4027 -0.4981  0.0824  0.5778  2.6253
##
## Random effects:
##  Groups    Name        Variance Std.Dev.
##  PP_number (Intercept) 0.2399   0.4898
##  Residual              0.1844   0.4295
## Number of obs: 400, groups:  PP_number, 100
##
## Fixed effects:
##                                  Estimate Std. Error         df t value
## (Intercept)                      0.524890   0.280477 110.765219   1.871
## conditieMF                      -0.345260   0.129488 285.000000  -2.666
## tijdPre                          0.103253   0.129488 285.000000   0.797
## Chronotype2                      0.143032   0.214771 186.281493   0.666
## Chronotype3                      0.228665   0.189251 186.615280   1.208
## Chronotype4                      0.211180   0.231196 188.008330   0.913
## Chronotype5                      0.237103   0.205014 186.901277   1.157
## Leeftijd                        -0.018758   0.006538  94.000000  -2.869
## conditieMF:tijdPre               0.530821   0.183123 285.000000   2.899
## conditieMF:Chronotype2          -0.219503   0.196126 285.000000  -1.119
## conditieMF:Chronotype3          -0.060863   0.173035 285.000000  -0.352
## conditieMF:Chronotype4          -0.342682   0.212466 285.000000  -1.613
## conditieMF:Chronotype5           0.069926   0.187645 285.000000   0.373
## tijdPre:Chronotype2              0.059451   0.196126 285.000000   0.303
## tijdPre:Chronotype3             -0.050912   0.173035 285.000000  -0.294
## tijdPre:Chronotype4             -0.054581   0.212466 285.000000  -0.257
## tijdPre:Chronotype5             -0.048683   0.187645 285.000000  -0.259
## conditieMF:tijdPre:Chronotype2  -0.017173   0.277364 285.000000  -0.062
## conditieMF:tijdPre:Chronotype3  -0.219855   0.244708 285.000000  -0.898
## conditieMF:tijdPre:Chronotype4  -0.042125   0.300473 285.000000  -0.140
## conditieMF:tijdPre:Chronotype5  -0.360902   0.265370 285.000000  -1.360
##                                Pr(>|t|)
## (Intercept)                     0.06393 .
## conditieMF                      0.00811 **
## tijdPre                         0.42589
## Chronotype2                     0.50625
## Chronotype3                     0.22847
## Chronotype4                     0.36219
## Chronotype5                     0.24894
## Leeftijd                        0.00508 **
## conditieMF:tijdPre              0.00404 **
## conditieMF:Chronotype2          0.26400
## conditieMF:Chronotype3          0.72529
## conditieMF:Chronotype4          0.10788
## conditieMF:Chronotype5          0.70968
## tijdPre:Chronotype2             0.76202
## tijdPre:Chronotype3             0.76880
## tijdPre:Chronotype4             0.79745
## tijdPre:Chronotype5             0.79548
## conditieMF:tijdPre:Chronotype2  0.95068
## conditieMF:tijdPre:Chronotype3  0.36971
## conditieMF:tijdPre:Chronotype4  0.88860
## conditieMF:tijdPre:Chronotype5  0.17491
## ---
## Signif. codes:  0 '***' 0.001 '**' 0.01 '*' 0.05 '.' 0.1 ' ' 1
##
## Correlation matrix not shown by default, as p = 21 > 12.
## Use print(x, correlation=TRUE)  or
##     vcov(x)        if you need it
print(anova_zscore_chronotype)
## Type III Analysis of Variance Table with Satterthwaite's method
##                          Sum Sq Mean Sq NumDF DenDF F value    Pr(>F)
## conditie                 6.0656  6.0656     1   285 32.8870 2.489e-08 ***
## tijd                     7.6458  7.6458     1   285 41.4549 5.105e-10 ***
## Chronotype               0.2406  0.0602     4    94  0.3262  0.859768
## Leeftijd                 1.5183  1.5183     1    94  8.2323  0.005082 **
## conditie:tijd            3.7993  3.7993     1   285 20.5995 8.357e-06 ***
## conditie:Chronotype      1.2323  0.3081     4   285  1.6703  0.156948
## tijd:Chronotype          1.0455  0.2614     4   285  1.4171  0.228334
## conditie:tijd:Chronotype 0.4916  0.1229     4   285  0.6663  0.615862
## ---
## Signif. codes:  0 '***' 0.001 '**' 0.01 '*' 0.05 '.' 0.1 ' ' 1
print(effectsize_Chronotype_zscore)
## # Effect Size for ANOVA (Type III)
##
## Parameter                | Eta2 (partial) |       95% CI
## --------------------------------------------------------
## conditie                 |           0.10 | [0.05, 1.00]
## tijd                     |           0.13 | [0.07, 1.00]
## Chronotype               |           0.01 | [0.00, 1.00]
## Leeftijd                 |           0.08 | [0.01, 1.00]
## conditie:tijd            |           0.07 | [0.03, 1.00]
## conditie:Chronotype      |           0.02 | [0.00, 1.00]
## tijd:Chronotype          |           0.02 | [0.00, 1.00]
## conditie:tijd:Chronotype |       9.26e-03 | [0.00, 1.00]
##
## - One-sided CIs: upper bound fixed at [1.00].
confint(model_Chronotype_zscore_2)
## Computing profile confidence intervals ...
##                                      2.5 %       97.5 %
## .sig01                          0.40248876  0.562089606
## .sigma                          0.38720284  0.454452351
## (Intercept)                    -0.01269844  1.062478613
## conditieMF                     -0.59341890 -0.097101349
## tijdPre                        -0.14490599  0.351411565
## Chronotype2                    -0.26776167  0.553824785
## Chronotype3                    -0.13331378  0.590643815
## Chronotype4                    -0.23101902  0.653378651
## Chronotype5                    -0.15502424  0.629230955
## Leeftijd                       -0.03130154 -0.006214419
## conditieMF:tijdPre              0.17987100  0.881770017
## conditieMF:Chronotype2         -0.59537291  0.156366770
## conditieMF:Chronotype3         -0.39247946  0.270752765
## conditieMF:Chronotype4         -0.74986684  0.064503564
## conditieMF:Chronotype5         -0.28968985  0.429542615
## tijdPre:Chronotype2            -0.31641921  0.435320469
## tijdPre:Chronotype3            -0.38252824  0.280703985
## tijdPre:Chronotype4            -0.46176584  0.352604561
## tijdPre:Chronotype5            -0.40829967  0.310932790
## conditieMF:tijdPre:Chronotype2 -0.54873287  0.514387586
## conditieMF:tijdPre:Chronotype3 -0.68883114  0.249120870
## conditieMF:tijdPre:Chronotype4 -0.61797163  0.533722038
## conditieMF:tijdPre:Chronotype5 -0.86947584  0.147672464
```

### Time trial

#### Outliers

outliersTTchronotype <- lapply(Data_Chronotype_TT[, -1], function(col) {

detect_outliers_z(col, Data_Chronotype_TT[, 1])

})

outliersTTchronotype

```
## $Leeftijd
## NULL
##
## $Chronotype
## NULL
##
## $CCQ
## [1] "MF_INDIFF_V1_020" "MF_INDIFF_V1_238"
##
## $TT_MF_Distance
## NULL
##
## $TT_CON_Distance
## NULL
##
## $CCQ_z
## [1] "MF_INDIFF_V1_020" "MF_INDIFF_V1_238"
```

#### Normality

shapiro_results_Chr_TT <- lapply(Data_Chronotype_TT, shapiro_test)

shapiro_results_Chr_TT

```
## $PP_number
## [1] NA
##
## $Leeftijd
## [1] 0.001222053
##
## $Chronotype
## [1] 3.729551e-07
##
## $CCQ
## [1] 3.2523e-08
##
## $TT_MF_Distance
## [1] 0.9511161
##
## $TT_CON_Distance
## [1] 0.7619543
##
## $CCQ_z
## [1] 3.2523e-08
```

#### Model

Data_Chronotype_TT_Pivot$Chronotype <- as.factor(Data_Chronotype_TT_Pivot$Chronotype)

Data_Chronotype_TT_Pivot$condition <- as.factor(Data_Chronotype_TT_Pivot$condition)

model_Chronotype_TT <- lmer(distance ~ condition*Chronotype + CCQ_z[, 1] + Leeftijd + (1 | PP_number), data = Data_Chronotype_TT_Pivot)

anova_Chronotype_TT <- anova(model_Chronotype_TT)

summary(model_Chronotype_TT)

```
## Linear mixed model fit by REML. t-tests use Satterthwaite's method [
## lmerModLmerTest]
## Formula: distance ~ condition * Chronotype + CCQ_z[, 1] + Leeftijd + (1 |
##     PP_number)
##    Data: Data_Chronotype_TT_Pivot
##
## REML criterion at convergence: 609.2
##
## Scaled residuals:
##      Min       1Q   Median       3Q      Max
## -2.43408 -0.38299 -0.00115  0.38379  2.22427
##
## Random effects:
##  Groups    Name        Variance Std.Dev.
##  PP_number (Intercept) 2.5137   1.5855
##  Residual              0.4321   0.6574
## Number of obs: 186, groups:  PP_number, 93
##
## Fixed effects:
##                          Estimate Std. Error        df t value Pr(>|t|)
## (Intercept)             11.496478   0.891544 88.255549  12.895   <2e-16 ***
## conditionMF             -0.195714   0.202866 88.000000  -0.965   0.3373
## Chronotype2             -0.389478   0.606182 98.885848  -0.643   0.5220
## Chronotype3              1.026985   0.515100 98.677954   1.994   0.0489 *
## Chronotype4              0.259185   0.661937 98.555293   0.392   0.6962
## Chronotype5              1.027581   0.554631 98.605216   1.853   0.0669 .
## CCQ_z[, 1]               0.410130   0.186182 85.999999   2.203   0.0303 *
## Leeftijd                 0.023335   0.021889 85.999997   1.066   0.2894
## conditionMF:Chronotype2  0.042143   0.320760 88.000000   0.131   0.8958
## conditionMF:Chronotype3 -0.002063   0.270488 88.000000  -0.008   0.9939
## conditionMF:Chronotype4  0.121169   0.346010 88.000000   0.350   0.7270
## conditionMF:Chronotype5 -0.087286   0.290460 88.000000  -0.301   0.7645
## ---
## Signif. codes:  0 '***' 0.001 '**' 0.01 '*' 0.05 '.' 0.1 ' ' 1
##
## Correlation of Fixed Effects:
##             (Intr) cndtMF Chrnt2 Chrnt3 Chrnt4 Chrnt5 CCQ_[1 Leftjd cMF:C2
## conditionMF -0.114
## Chronotype2 -0.453  0.167
## Chronotype3 -0.519  0.197  0.500
## Chronotype4 -0.420  0.153  0.395  0.470
## Chronotype5 -0.470  0.183  0.463  0.550  0.449
## CCQ_z[, 1]   0.228  0.000 -0.038  0.008  0.112  0.095
## Leeftijd    -0.906  0.000  0.212  0.230  0.190  0.196 -0.309
## cndtnMF:Ch2  0.072 -0.632 -0.265 -0.125 -0.097 -0.116  0.000  0.000
## cndtnMF:Ch3  0.085 -0.750 -0.125 -0.263 -0.115 -0.137  0.000  0.000  0.474
## cndtnMF:Ch4  0.067 -0.586 -0.098 -0.115 -0.261 -0.107  0.000  0.000  0.371
## cndtnMF:Ch5  0.079 -0.698 -0.117 -0.138 -0.107 -0.262  0.000  0.000  0.442
##             cMF:C3 cMF:C4
## conditionMF
## Chronotype2
## Chronotype3
## Chronotype4
## Chronotype5
## CCQ_z[, 1]
## Leeftijd
## cndtnMF:Ch2
## cndtnMF:Ch3
## cndtnMF:Ch4  0.440
## cndtnMF:Ch5  0.524  0.409
print(anova_Chronotype_TT)
## Type III Analysis of Variance Table with Satterthwaite's method
##                      Sum Sq Mean Sq NumDF DenDF F value  Pr(>F)
## condition            1.3777 1.37767     1    88  3.1881 0.07762 .
## Chronotype           4.3688 1.09220     4    86  2.5275 0.04641 *
## CCQ_z[, 1]           2.0969 2.09689     1    86  4.8525 0.03028 *
## Leeftijd             0.4911 0.49112     1    86  1.1365 0.28937
## condition:Chronotype 0.1693 0.04234     4    88  0.0980 0.98285
## ---
## Signif. codes:  0 '***' 0.001 '**' 0.01 '*' 0.05 '.' 0.1 ' ' 1
```

The model showed no influence of age. An additional model was therefore constructed.

model_Chronotype_TT_2 <- lmer(distance ~ condition*Chronotype + CCQ_z[, 1] + (1 | PP_number), data = Data_Chronotype_TT_Pivot)

anova_Chronotype_TT_2 <- anova(model_Chronotype_TT_2)

effectsize_Chronotype_TT <- eta_squared(model_Chronotype_TT_2, partial = TRUE)

summary(model_Chronotype_TT_2)

```
## Linear mixed model fit by REML. t-tests use Satterthwaite's method [
## lmerModLmerTest]
## Formula: distance ~ condition * Chronotype + CCQ_z[, 1] + (1 | PP_number)
##    Data: Data_Chronotype_TT_Pivot
##
## REML criterion at convergence: 604.5
##
## Scaled residuals:
##      Min       1Q   Median       3Q      Max
## -2.44106 -0.37476  0.01194  0.36891  2.21729
##
## Random effects:
##  Groups    Name        Variance Std.Dev.
##  PP_number (Intercept) 2.5179   1.5868
##  Residual              0.4321   0.6574
## Number of obs: 186, groups:  PP_number, 93
##
## Fixed effects:
##                           Estimate Std. Error         df t value Pr(>|t|)
## (Intercept)              12.357437   0.377962 100.430363  32.695  < 2e-16 ***
## conditionMF              -0.195714   0.202866  88.000002  -0.965  0.33732
## Chronotype2              -0.526297   0.592871 100.660255  -0.888  0.37681
## Chronotype3               0.900469   0.501607 100.564370   1.795  0.07563 .
## Chronotype4               0.124848   0.650306 100.182996   0.192  0.84814
## Chronotype5               0.911527   0.544239 100.268982   1.675  0.09708 .
## CCQ_z[, 1]                0.471526   0.177190  86.999995   2.661  0.00927 **
## conditionMF:Chronotype2   0.042143   0.320760  88.000002   0.131  0.89577
## conditionMF:Chronotype3  -0.002063   0.270488  88.000002  -0.008  0.99393
## conditionMF:Chronotype4   0.121169   0.346010  88.000002   0.350  0.72703
## conditionMF:Chronotype5  -0.087286   0.290460  88.000002  -0.301  0.76450
## ---
## Signif. codes:  0 '***' 0.001 '**' 0.01 '*' 0.05 '.' 0.1 ' ' 1
##
## Correlation of Fixed Effects:
##             (Intr) cndtMF Chrnt2 Chrnt3 Chrnt4 Chrnt5 CCQ_[1 cMF:C2 cMF:C3
## conditionMF -0.268
## Chronotype2 -0.631  0.171
## Chronotype3 -0.752  0.202  0.475
## Chronotype4 -0.595  0.156  0.370  0.446
## Chronotype5 -0.704  0.186  0.440  0.529  0.427
## CCQ_z[, 1]  -0.129  0.000  0.029  0.086  0.183  0.167
## cndtnMF:Ch2  0.170 -0.632 -0.271 -0.128 -0.099 -0.118  0.000
## cndtnMF:Ch3  0.201 -0.750 -0.128 -0.270 -0.117 -0.140  0.000  0.474
## cndtnMF:Ch4  0.157 -0.586 -0.100 -0.119 -0.266 -0.109  0.000  0.371  0.440
## cndtnMF:Ch5  0.187 -0.698 -0.119 -0.141 -0.109 -0.267  0.000  0.442  0.524
##             cMF:C4
## conditionMF
## Chronotype2
## Chronotype3
## Chronotype4
## Chronotype5
## CCQ_z[, 1]
## cndtnMF:Ch2
## cndtnMF:Ch3
## cndtnMF:Ch4
## cndtnMF:Ch5  0.409
print(anova_Chronotype_TT_2)
## Type III Analysis of Variance Table with Satterthwaite's method
##                      Sum Sq Mean Sq NumDF DenDF F value   Pr(>F)
## condition            1.3777 1.37767     1    88  3.1881 0.077620 .
## Chronotype           4.1298 1.03245     4    87  2.3892 0.056980 .
## CCQ_z[, 1]           3.0601 3.06014     1    87  7.0816 0.009274 **
## condition:Chronotype 0.1693 0.04234     4    88  0.0980 0.982848
## ---
## Signif. codes:  0 '***' 0.001 '**' 0.01 '*' 0.05 '.' 0.1 ' ' 1
print(effectsize_Chronotype_TT)
## # Effect Size for ANOVA (Type III)
##
## Parameter            | Eta2 (partial) |       95% CI
## ----------------------------------------------------
## condition            |           0.03 | [0.00, 1.00]
## Chronotype           |           0.10 | [0.00, 1.00]
## CCQ_z[, 1]           |           0.08 | [0.01, 1.00]
## condition:Chronotype |       4.43e-03 | [0.00, 1.00]
##
## - One-sided CIs: upper bound fixed at [1.00].
confint(model_Chronotype_TT_2)
## Computing profile confidence intervals ...
##                               2.5 %     97.5 %
## .sig01                   1.31761089  1.8044422
## .sigma                   0.55749788  0.7436290
## (Intercept)             11.63417217 13.0807016
## conditionMF             -0.58651753  0.1950890
## Chronotype2             -1.66079383  0.6081994
## Chronotype3             -0.05939417  1.8603320
## Chronotype4             -1.11959047  1.3692864
## Chronotype5             -0.12993447  1.9529887
## CCQ_z[, 1]               0.13213034  0.8109210
## conditionMF:Chronotype2 -0.57577133  0.6600570
## conditionMF:Chronotype3 -0.52313449  0.5190075
## conditionMF:Chronotype4 -0.54538743  0.7877251
## conditionMF:Chronotype5 -0.64683081  0.4722594
```

### Rating of perceived exertion

#### Outliers

outliersRPEChronotype<- lapply(Data_Chronotype_RPE[, -1], function(col) {

detect_outliers_z(col, Data_Chronotype_RPE[, 1])

})

outliersRPEChronotype

```
## $Leeftijd
## NULL
##
## $Chronotype
## NULL
##
## $CCQ
## [1] "MF_INDIFF_V1_020" "MF_INDIFF_V1_238"
##
## $RPE_1_MF
## [1] "MF_INDIFF_V1_177" "MF_INDIFF_V1_257"
##
## $RPE_2_MF
## NULL
##
## $RPE_3_MF
## NULL
##
## $RPE_4_MF
## NULL
##
## $RPE_5_MF
## NULL
##
## $RPE_6_MF
## [1] "MF_INDIFF_V1_011"
##
## $RPE_1_CON
## [1] "MF_INDIFF_V1_038" "MF_INDIFF_V1_177"
##
## $RPE_2_CON
## NULL
##
## $RPE_3_CON
## NULL
##
## $RPE_4_CON
## NULL
##
## $RPE_5_CON
## NULL
##
## $RPE_6_CON
## [1] "MF_INDIFF_V1_011"
##
## $CCQ_z
## [1] "MF_INDIFF_V1_020" "MF_INDIFF_V1_238"
```

#### Normality

shapiro_results_Chr_RPE <- lapply(Data_Chronotype_RPE, shapiro_test)

shapiro_results_Chr_RPE

```
## $PP_number
## [1] NA
##
## $Leeftijd
## [1] 0.0008358021
##
## $Chronotype
## [1] 2.976966e-07
##
## $CCQ
## [1] 1.820455e-08
##
## $RPE_1_MF
## [1] 3.876412e-07
##
## $RPE_2_MF
## [1] 0.2421655
##
## $RPE_3_MF
## [1] 0.1863813
##
## $RPE_4_MF
## [1] 0.002064525
##
## $RPE_5_MF
## [1] 0.001908974
##
## $RPE_6_MF
## [1] 0.0004291766
##
## $RPE_1_CON
## [1] 7.433106e-08
##
## $RPE_2_CON
## [1] 0.05579948
##
## $RPE_3_CON
## [1] 0.1538425
##
## $RPE_4_CON
## [1] 0.03547011
##
## $RPE_5_CON
## [1] 0.05145485
##
## $RPE_6_CON
## [1] 0.0001363645
##
## $CCQ_z
## [1] 1.820455e-08
```

#### Model

Data_Chronotype_RPE_Pivot$Chronotype <- as.factor(Data_Chronotype_RPE_Pivot$Chronotype)

Data_Chronotype_RPE_Pivot$condition <- as.factor(Data_Chronotype_RPE_Pivot$condition)

Data_Chronotype_RPE_Pivot$time <- as.factor(Data_Chronotype_RPE_Pivot$time)

model_Chronotype_RPE <- lmer(value ~ condition*time*Chronotype + CCQ_z[, 1] + Leeftijd + (1 | PP_number), data = Data_Chronotype_RPE_Pivot)

summary(model_Chronotype_RPE)

```
## Linear mixed model fit by REML. t-tests use Satterthwaite's method [
## lmerModLmerTest]
## Formula: value ~ condition * time * Chronotype + CCQ_z[, 1] + Leeftijd +
##     (1 | PP_number)
##    Data: Data_Chronotype_RPE_Pivot
##
## REML criterion at convergence: 9035.3
##
## Scaled residuals:
##     Min      1Q  Median      3Q     Max
## -3.1074 -0.6132  0.0509  0.6180  3.2230
##
## Random effects:
##  Groups    Name        Variance Std.Dev.
##  PP_number (Intercept) 103.0    10.15
##  Residual              112.9    10.63
## Number of obs: 1200, groups:  PP_number, 100
##
## Fixed effects:
##                                Estimate Std. Error        df t value Pr(>|t|)
## (Intercept)                      7.6104     5.8917  124.1833   1.292   0.1989
## conditionMF                      1.2727     3.2042 1045.0000   0.397   0.6913
## time2                           26.7273     3.2042 1045.0000   8.341  2.3e-16
## time3                           35.3636     3.2042 1045.0000  11.037  < 2e-16
## time4                           46.0455     3.2042 1045.0000  14.370  < 2e-16
## time5                           55.3636     3.2042 1045.0000  17.279  < 2e-16
## time6                           64.0000     3.2042 1045.0000  19.974  < 2e-16
## Chronotype2                      1.0820     4.8228  303.5889   0.224   0.8226
## Chronotype3                      0.3870     4.2504  304.5730   0.091   0.9275
## Chronotype4                      9.2994     5.2243  303.6515   1.780   0.0761
## Chronotype5                     -2.7510     4.6177  302.9078  -0.596   0.5518
## CCQ_z[, 1]                       0.8067     1.1507   93.0000   0.701   0.4850
## Leeftijd                         0.1706     0.1362   93.0000   1.253   0.2135
## conditionMF:time2               -1.5000     4.5314 1045.0000  -0.331   0.7407
## conditionMF:time3                3.7273     4.5314 1045.0000   0.823   0.4110
## conditionMF:time4                1.4545     4.5314 1045.0000   0.321   0.7483
## conditionMF:time5                2.8182     4.5314 1045.0000   0.622   0.5341
## conditionMF:time6                3.2727     4.5314 1045.0000   0.722   0.4703
## conditionMF:Chronotype2         -0.3316     4.8532 1045.0000  -0.068   0.9455
## conditionMF:Chronotype3          1.3701     4.2818 1045.0000   0.320   0.7490
## conditionMF:Chronotype4         -2.1958     5.2575 1045.0000  -0.418   0.6763
## conditionMF:Chronotype5          1.2773     4.6433 1045.0000   0.275   0.7833
## time2:Chronotype2               -5.4332     4.8532 1045.0000  -1.120   0.2632
## time3:Chronotype2               -3.1872     4.8532 1045.0000  -0.657   0.5115
## time4:Chronotype2               -5.8102     4.8532 1045.0000  -1.197   0.2315
## time5:Chronotype2               -3.4813     4.8532 1045.0000  -0.717   0.4733
## time6:Chronotype2               -3.0000     4.8532 1045.0000  -0.618   0.5366
## time2:Chronotype3               -3.0487     4.2818 1045.0000  -0.712   0.4766
## time3:Chronotype3                1.2792     4.2818 1045.0000   0.299   0.7652
## time4:Chronotype3               -2.3312     4.2818 1045.0000  -0.544   0.5863
## time5:Chronotype3                0.3506     4.2818 1045.0000   0.082   0.9347
## time6:Chronotype3                0.3571     4.2818 1045.0000   0.083   0.9335
## time2:Chronotype4               -6.7273     5.2575 1045.0000  -1.280   0.2010
## time3:Chronotype4               -6.9021     5.2575 1045.0000  -1.313   0.1895
## time4:Chronotype4              -10.6608     5.2575 1045.0000  -2.028   0.0428
## time5:Chronotype4              -12.6713     5.2575 1045.0000  -2.410   0.0161
## time6:Chronotype4              -10.9231     5.2575 1045.0000  -2.078   0.0380
## time2:Chronotype5                6.6227     4.6433 1045.0000   1.426   0.1541
## time3:Chronotype5                7.1364     4.6433 1045.0000   1.537   0.1246
## time4:Chronotype5                3.3545     4.6433 1045.0000   0.722   0.4702
## time5:Chronotype5                6.6364     4.6433 1045.0000   1.429   0.1532
## time6:Chronotype5                7.3500     4.6433 1045.0000   1.583   0.1137
## conditionMF:time2:Chronotype2   -1.2059     6.8634 1045.0000  -0.176   0.8606
## conditionMF:time3:Chronotype2   -4.6684     6.8634 1045.0000  -0.680   0.4965
## conditionMF:time4:Chronotype2   -3.3957     6.8634 1045.0000  -0.495   0.6209
## conditionMF:time5:Chronotype2   -8.4652     6.8634 1045.0000  -1.233   0.2177
## conditionMF:time6:Chronotype2   -6.8610     6.8634 1045.0000  -1.000   0.3177
## conditionMF:time2:Chronotype3    3.1071     6.0553 1045.0000   0.513   0.6080
## conditionMF:time3:Chronotype3   -3.9058     6.0553 1045.0000  -0.645   0.5191
## conditionMF:time4:Chronotype3    1.2240     6.0553 1045.0000   0.202   0.8398
## conditionMF:time5:Chronotype3   -2.4610     6.0553 1045.0000  -0.406   0.6845
## conditionMF:time6:Chronotype3   -1.8799     6.0553 1045.0000  -0.310   0.7563
## conditionMF:time2:Chronotype4    3.9615     7.4352 1045.0000   0.533   0.5943
## conditionMF:time3:Chronotype4   -3.9580     7.4352 1045.0000  -0.532   0.5946
## conditionMF:time4:Chronotype4    2.9301     7.4352 1045.0000   0.394   0.6936
## conditionMF:time5:Chronotype4    3.8741     7.4352 1045.0000   0.521   0.6024
## conditionMF:time6:Chronotype4   -0.8112     7.4352 1045.0000  -0.109   0.9131
## conditionMF:time2:Chronotype5    2.2000     6.5666 1045.0000   0.335   0.7377
## conditionMF:time3:Chronotype5   -3.7773     6.5666 1045.0000  -0.575   0.5653
## conditionMF:time4:Chronotype5   -0.6545     6.5666 1045.0000  -0.100   0.9206
## conditionMF:time5:Chronotype5   -7.0182     6.5666 1045.0000  -1.069   0.2854
## conditionMF:time6:Chronotype5   -4.9227     6.5666 1045.0000  -0.750   0.4536
##
## (Intercept)
## conditionMF
## time2                         ***
## time3                         ***
## time4                         ***
## time5                         ***
## time6                         ***
## Chronotype2
## Chronotype3
## Chronotype4                   .
## Chronotype5
## CCQ_z[, 1]
## Leeftijd
## conditionMF:time2
## conditionMF:time3
## conditionMF:time4
## conditionMF:time5
## conditionMF:time6
## conditionMF:Chronotype2
## conditionMF:Chronotype3
## conditionMF:Chronotype4
## conditionMF:Chronotype5
## time2:Chronotype2
## time3:Chronotype2
## time4:Chronotype2
## time5:Chronotype2
## time6:Chronotype2
## time2:Chronotype3
## time3:Chronotype3
## time4:Chronotype3
## time5:Chronotype3
## time6:Chronotype3
## time2:Chronotype4
## time3:Chronotype4
## time4:Chronotype4             *
## time5:Chronotype4             *
## time6:Chronotype4             *
## time2:Chronotype5
## time3:Chronotype5
## time4:Chronotype5
## time5:Chronotype5
## time6:Chronotype5
## conditionMF:time2:Chronotype2
## conditionMF:time3:Chronotype2
## conditionMF:time4:Chronotype2
## conditionMF:time5:Chronotype2
## conditionMF:time6:Chronotype2
## conditionMF:time2:Chronotype3
## conditionMF:time3:Chronotype3
## conditionMF:time4:Chronotype3
## conditionMF:time5:Chronotype3
## conditionMF:time6:Chronotype3
## conditionMF:time2:Chronotype4
## conditionMF:time3:Chronotype4
## conditionMF:time4:Chronotype4
## conditionMF:time5:Chronotype4
## conditionMF:time6:Chronotype4
## conditionMF:time2:Chronotype5
## conditionMF:time3:Chronotype5
## conditionMF:time4:Chronotype5
## conditionMF:time5:Chronotype5
## conditionMF:time6:Chronotype5
## ---
## Signif. codes:  0 '***' 0.001 '**' 0.01 '*' 0.05 '.' 0.1 ' ' 1
##
## Correlation matrix not shown by default, as p = 62 > 12.
## Use print(x, correlation=TRUE)  or
##     vcov(x)        if you need it
```

The model showed no influence of age or caffeine consumption. An additional model was therefore constructed.

model_Chronotype_RPE_2 <- lmer(value ~ condition*time*Chronotype + (1 | PP_number), data = Data_Chronotype_RPE_Pivot)

anova_Chronotype_RPE <- anova(model_Chronotype_RPE_2)

effectsize_Chronotype_RPE <- eta_squared(model_Chronotype_RPE_2, partial = TRUE)

summary(model_Chronotype_RPE_2)

```
## Linear mixed model fit by REML. t-tests use Satterthwaite's method [
## lmerModLmerTest]
## Formula: value ~ condition * time * Chronotype + (1 | PP_number)
##    Data: Data_Chronotype_RPE_Pivot
##
## REML criterion at convergence: 9038
##
## Scaled residuals:
##     Min      1Q  Median      3Q     Max
## -3.0908 -0.6114  0.0503  0.6156  3.2396
##
## Random effects:
##  Groups    Name        Variance Std.Dev.
##  PP_number (Intercept) 104.0    10.20
##  Residual              112.9    10.63
## Number of obs: 1200, groups:  PP_number, 100
##
## Fixed effects:
##                                Estimate Std. Error        df t value Pr(>|t|)
## (Intercept)                     14.1818     3.1402  323.1048   4.516 8.84e-06
## conditionMF                      1.2727     3.2042 1045.0000   0.397   0.6913
## time2                           26.7273     3.2042 1045.0000   8.341 2.30e-16
## time3                           35.3636     3.2042 1045.0000  11.037  < 2e-16
## time4                           46.0455     3.2042 1045.0000  14.370  < 2e-16
## time5                           55.3636     3.2042 1045.0000  17.279  < 2e-16
## time6                           64.0000     3.2042 1045.0000  19.974  < 2e-16
## Chronotype2                     -0.1818     4.7563  323.1048  -0.038   0.9695
## Chronotype3                     -0.7175     4.1963  323.1048  -0.171   0.8643
## Chronotype4                      7.7413     5.1525  323.1048   1.502   0.1340
## Chronotype5                     -4.1818     4.5506  323.1048  -0.919   0.3588
## conditionMF:time2               -1.5000     4.5314 1045.0000  -0.331   0.7407
## conditionMF:time3                3.7273     4.5314 1045.0000   0.823   0.4110
## conditionMF:time4                1.4545     4.5314 1045.0000   0.321   0.7483
## conditionMF:time5                2.8182     4.5314 1045.0000   0.622   0.5341
## conditionMF:time6                3.2727     4.5314 1045.0000   0.722   0.4703
## conditionMF:Chronotype2         -0.3316     4.8532 1045.0000  -0.068   0.9455
## conditionMF:Chronotype3          1.3701     4.2818 1045.0000   0.320   0.7490
## conditionMF:Chronotype4         -2.1958     5.2575 1045.0000  -0.418   0.6763
## conditionMF:Chronotype5          1.2773     4.6433 1045.0000   0.275   0.7833
## time2:Chronotype2               -5.4332     4.8532 1045.0000  -1.120   0.2632
## time3:Chronotype2               -3.1872     4.8532 1045.0000  -0.657   0.5115
## time4:Chronotype2               -5.8102     4.8532 1045.0000  -1.197   0.2315
## time5:Chronotype2               -3.4813     4.8532 1045.0000  -0.717   0.4733
## time6:Chronotype2               -3.0000     4.8532 1045.0000  -0.618   0.5366
## time2:Chronotype3               -3.0487     4.2818 1045.0000  -0.712   0.4766
## time3:Chronotype3                1.2792     4.2818 1045.0000   0.299   0.7652
## time4:Chronotype3               -2.3312     4.2818 1045.0000  -0.544   0.5863
## time5:Chronotype3                0.3506     4.2818 1045.0000   0.082   0.9347
## time6:Chronotype3                0.3571     4.2818 1045.0000   0.083   0.9335
## time2:Chronotype4               -6.7273     5.2575 1045.0000  -1.280   0.2010
## time3:Chronotype4               -6.9021     5.2575 1045.0000  -1.313   0.1895
## time4:Chronotype4              -10.6608     5.2575 1045.0000  -2.028   0.0428
## time5:Chronotype4              -12.6713     5.2575 1045.0000  -2.410   0.0161
## time6:Chronotype4              -10.9231     5.2575 1045.0000  -2.078   0.0380
## time2:Chronotype5                6.6227     4.6433 1045.0000   1.426   0.1541
## time3:Chronotype5                7.1364     4.6433 1045.0000   1.537   0.1246
## time4:Chronotype5                3.3545     4.6433 1045.0000   0.722   0.4702
## time5:Chronotype5                6.6364     4.6433 1045.0000   1.429   0.1532
## time6:Chronotype5                7.3500     4.6433 1045.0000   1.583   0.1137
## conditionMF:time2:Chronotype2   -1.2059     6.8634 1045.0000  -0.176   0.8606
## conditionMF:time3:Chronotype2   -4.6684     6.8634 1045.0000  -0.680   0.4965
## conditionMF:time4:Chronotype2   -3.3957     6.8634 1045.0000  -0.495   0.6209
## conditionMF:time5:Chronotype2   -8.4652     6.8634 1045.0000  -1.233   0.2177
## conditionMF:time6:Chronotype2   -6.8610     6.8634 1045.0000  -1.000   0.3177
## conditionMF:time2:Chronotype3    3.1071     6.0553 1045.0000   0.513   0.6080
## conditionMF:time3:Chronotype3   -3.9058     6.0553 1045.0000  -0.645   0.5191
## conditionMF:time4:Chronotype3    1.2240     6.0553 1045.0000   0.202   0.8398
## conditionMF:time5:Chronotype3   -2.4610     6.0553 1045.0000  -0.406   0.6845
## conditionMF:time6:Chronotype3   -1.8799     6.0553 1045.0000  -0.310   0.7563
## conditionMF:time2:Chronotype4    3.9615     7.4352 1045.0000   0.533   0.5943
## conditionMF:time3:Chronotype4   -3.9580     7.4352 1045.0000  -0.532   0.5946
## conditionMF:time4:Chronotype4    2.9301     7.4352 1045.0000   0.394   0.6936
## conditionMF:time5:Chronotype4    3.8741     7.4352 1045.0000   0.521   0.6024
## conditionMF:time6:Chronotype4   -0.8112     7.4352 1045.0000  -0.109   0.9131
## conditionMF:time2:Chronotype5    2.2000     6.5666 1045.0000   0.335   0.7377
## conditionMF:time3:Chronotype5   -3.7773     6.5666 1045.0000  -0.575   0.5653
## conditionMF:time4:Chronotype5   -0.6545     6.5666 1045.0000  -0.100   0.9206
## conditionMF:time5:Chronotype5   -7.0182     6.5666 1045.0000  -1.069   0.2854
## conditionMF:time6:Chronotype5   -4.9227     6.5666 1045.0000  -0.750   0.4536
##
## (Intercept)                   ***
## conditionMF
## time2                         ***
## time3                         ***
## time4                         ***
## time5                         ***
## time6                         ***
## Chronotype2
## Chronotype3
## Chronotype4
## Chronotype5
## conditionMF:time2
## conditionMF:time3
## conditionMF:time4
## conditionMF:time5
## conditionMF:time6
## conditionMF:Chronotype2
## conditionMF:Chronotype3
## conditionMF:Chronotype4
## conditionMF:Chronotype5
## time2:Chronotype2
## time3:Chronotype2
## time4:Chronotype2
## time5:Chronotype2
## time6:Chronotype2
## time2:Chronotype3
## time3:Chronotype3
## time4:Chronotype3
## time5:Chronotype3
## time6:Chronotype3
## time2:Chronotype4
## time3:Chronotype4
## time4:Chronotype4             *
## time5:Chronotype4             *
## time6:Chronotype4             *
## time2:Chronotype5
## time3:Chronotype5
## time4:Chronotype5
## time5:Chronotype5
## time6:Chronotype5
## conditionMF:time2:Chronotype2
## conditionMF:time3:Chronotype2
## conditionMF:time4:Chronotype2
## conditionMF:time5:Chronotype2
## conditionMF:time6:Chronotype2
## conditionMF:time2:Chronotype3
## conditionMF:time3:Chronotype3
## conditionMF:time4:Chronotype3
## conditionMF:time5:Chronotype3
## conditionMF:time6:Chronotype3
## conditionMF:time2:Chronotype4
## conditionMF:time3:Chronotype4
## conditionMF:time4:Chronotype4
## conditionMF:time5:Chronotype4
## conditionMF:time6:Chronotype4
## conditionMF:time2:Chronotype5
## conditionMF:time3:Chronotype5
## conditionMF:time4:Chronotype5
## conditionMF:time5:Chronotype5
## conditionMF:time6:Chronotype5
## ---
## Signif. codes:  0 '***' 0.001 '**' 0.01 '*' 0.05 '.' 0.1 ' ' 1
##
## Correlation matrix not shown by default, as p = 60 > 12.
## Use print(x, correlation=TRUE)  or
##     vcov(x)        if you need it
print(anova_Chronotype_RPE)
## Type III Analysis of Variance Table with Satterthwaite's method
##                           Sum Sq Mean Sq NumDF DenDF  F value    Pr(>F)
## condition                    815     815     1  1045   7.2124  0.007355 **
## time                      469319   93864     5  1045 831.1348 < 2.2e-16 ***
## Chronotype                   461     115     4    95   1.0200  0.401083
## condition:time                73      15     5  1045   0.1294  0.985715
## condition:Chronotype         914     229     4  1045   2.0241  0.088942 .
## time:Chronotype             4348     217    20  1045   1.9249  0.008537 **
## condition:time:Chronotype    807      40    20  1045   0.3572  0.996050
## ---
## Signif. codes:  0 '***' 0.001 '**' 0.01 '*' 0.05 '.' 0.1 ' ' 1
print(effectsize_Chronotype_RPE)
## # Effect Size for ANOVA (Type III)
##
## Parameter                 | Eta2 (partial) |       95% CI
## ---------------------------------------------------------
## condition                 |       6.85e-03 | [0.00, 1.00]
## time                      |           0.80 | [0.78, 1.00]
## Chronotype                |           0.04 | [0.00, 1.00]
## condition:time            |       6.19e-04 | [0.00, 1.00]
## condition:Chronotype      |       7.69e-03 | [0.00, 1.00]
## time:Chronotype           |           0.04 | [0.00, 1.00]
## condition:time:Chronotype |       6.79e-03 | [0.00, 1.00]
##
## - One-sided CIs: upper bound fixed at [1.00].
confint(model_Chronotype_RPE_2)
## Computing profile confidence intervals ...
##                                    2.5 %     97.5 %
## .sig01                          8.585627 11.6252123
## .sigma                          9.939781 10.8063547
## (Intercept)                     8.165829 20.1978075
## conditionMF                    -4.853685  7.3991392
## time2                          20.600861 32.8536846
## time3                          29.237224 41.4900483
## time4                          39.919043 52.1718664
## time5                          49.237224 61.4900483
## time6                          57.873588 70.1264119
## Chronotype2                    -9.293843  8.9302067
## Chronotype3                    -8.756736  7.3216714
## Chronotype4                    -2.129929 17.6124462
## Chronotype5                   -12.899815  4.5361785
## conditionMF:time2             -10.164055  7.1640548
## conditionMF:time3              -4.936782 12.3913275
## conditionMF:time4              -7.209509 10.1186003
## conditionMF:time5              -5.845873 11.4822366
## conditionMF:time6              -5.391328 11.9367821
## conditionMF:Chronotype2        -9.610826  8.9477240
## conditionMF:Chronotype3        -6.816632  9.5568921
## conditionMF:Chronotype4       -12.248176  7.8565675
## conditionMF:Chronotype5        -7.600742 10.1552870
## time2:Chronotype2             -14.712430  3.8461197
## time3:Chronotype2             -12.466441  6.0921090
## time4:Chronotype2             -15.089435  3.4691144
## time5:Chronotype2             -12.760558  5.7979914
## time6:Chronotype2             -12.279275  6.2792748
## time2:Chronotype3             -11.235464  5.1380610
## time3:Chronotype3              -6.907541  9.4659830
## time4:Chronotype3             -10.517931  5.8555934
## time5:Chronotype3              -7.836113  8.5374116
## time6:Chronotype3              -7.829619  8.5439051
## time2:Chronotype4             -16.779644  3.3250990
## time3:Chronotype4             -16.954470  3.1502738
## time4:Chronotype4             -20.713211 -0.6084675
## time5:Chronotype4             -22.723700 -2.6189570
## time6:Chronotype4             -20.975449 -0.8707052
## time2:Chronotype5              -2.255287 15.5007416
## time3:Chronotype5              -1.741651 16.0143779
## time4:Chronotype5              -5.523469 12.2325597
## time5:Chronotype5              -2.241651 15.5143779
## time6:Chronotype5              -1.528014 16.2280143
## conditionMF:time2:Chronotype2 -14.328759 11.9169939
## conditionMF:time3:Chronotype2 -17.791325  8.4544270
## conditionMF:time4:Chronotype2 -16.518598  9.7271543
## conditionMF:time5:Chronotype2 -21.588117  4.6576356
## conditionMF:time6:Chronotype2 -19.983839  6.2619137
## conditionMF:time2:Chronotype3  -8.470687 14.6849731
## conditionMF:time3:Chronotype3 -15.483674  7.6719861
## conditionMF:time4:Chronotype3 -10.353804 12.8018562
## conditionMF:time5:Chronotype3 -14.038869  9.1167913
## conditionMF:time6:Chronotype3 -13.457700  9.6979601
## conditionMF:time2:Chronotype4 -10.254662 18.1777388
## conditionMF:time3:Chronotype4 -18.174242 10.2581584
## conditionMF:time4:Chronotype4 -11.286130 17.1462703
## conditionMF:time5:Chronotype4 -10.342075 18.0903263
## conditionMF:time6:Chronotype4 -15.027389 13.4050116
## conditionMF:time2:Chronotype5 -10.355408 14.7554082
## conditionMF:time3:Chronotype5 -16.332681  8.7781355
## conditionMF:time4:Chronotype5 -13.209954 11.9008628
## conditionMF:time5:Chronotype5 -19.573590  5.5372264
## conditionMF:time6:Chronotype5 -17.478135  7.6326809
```

#### Post HOC

```
emmeans(model_Chronotype_RPE_2, ~ time | Chronotype)
## NOTE: Results may be misleading due to involvement in interactions
## Chronotype = 1:
##  time emmean   SE  df lower.CL upper.CL
##  1      14.8 2.70 187     9.49     20.1
##  2      40.8 2.70 187    35.47     46.1
##  3      52.0 2.70 187    46.72     57.4
##  4      61.6 2.70 187    56.26     66.9
##  5      71.6 2.70 187    66.26     76.9
##  6      80.5 2.70 187    75.13     85.8
##
## Chronotype = 2:
##  time emmean   SE  df lower.CL upper.CL
##  1      14.5 3.07 187     8.41     20.5
##  2      34.4 3.07 187    28.35     40.5
##  3      46.2 3.07 187    40.12     52.2
##  4      53.7 3.07 187    47.67     59.8
##  5      63.5 3.07 187    57.47     69.6
##  6      73.7 3.07 187    67.62     79.7
##
## Chronotype = 3:
##  time emmean   SE  df lower.CL upper.CL
##  1      14.8 2.39 187    10.06     19.5
##  2      39.3 2.39 187    34.55     44.0
##  3      51.3 2.39 187    46.62     56.1
##  4      59.8 2.39 187    55.12     64.6
##  5      70.7 2.39 187    65.96     75.4
##  6      79.8 2.39 187    75.12     84.6
##
## Chronotype = 4:
##  time emmean   SE  df lower.CL upper.CL
##  1      21.5 3.51 187    14.53     28.4
##  2      42.7 3.51 187    35.76     49.6
##  3      49.8 3.51 187    42.88     56.7
##  4      59.0 3.51 187    52.11     66.0
##  5      67.5 3.51 187    60.57     74.4
##  6      75.8 3.51 187    68.84     82.7
##
## Chronotype = 5:
##  time emmean   SE  df lower.CL upper.CL
##  1      11.3 2.83 187     5.69     16.9
##  2      45.0 2.83 187    39.39     50.6
##  3      53.8 2.83 187    48.16     59.3
##  4      61.1 2.83 187    55.49     66.7
##  5      71.2 2.83 187    65.59     76.8
##  6      81.8 2.83 187    76.21     87.4
##
## Results are averaged over the levels of: condition
## Degrees-of-freedom method: kenward-roger
## Confidence level used: 0.95
pairs(emmeans(model_Chronotype_RPE_2, ~ time | Chronotype))
## NOTE: Results may be misleading due to involvement in interactions
## Chronotype = 1:
##  contrast      estimate   SE   df t.ratio p.value
##  time1 - time2   -25.98 2.27 1045 -11.465 <0.0001
##  time1 - time3   -37.23 2.27 1045 -16.431 <0.0001
##  time1 - time4   -46.77 2.27 1045 -20.644 <0.0001
##  time1 - time5   -56.77 2.27 1045 -25.058 <0.0001
##  time1 - time6   -65.64 2.27 1045 -28.970 <0.0001
##  time2 - time3   -11.25 2.27 1045  -4.965 <0.0001
##  time2 - time4   -20.80 2.27 1045  -9.178 <0.0001
##  time2 - time5   -30.80 2.27 1045 -13.592 <0.0001
##  time2 - time6   -39.66 2.27 1045 -17.504 <0.0001
##  time3 - time4    -9.55 2.27 1045  -4.213  0.0004
##  time3 - time5   -19.55 2.27 1045  -8.627 <0.0001
##  time3 - time6   -28.41 2.27 1045 -12.539 <0.0001
##  time4 - time5   -10.00 2.27 1045  -4.414  0.0002
##  time4 - time6   -18.86 2.27 1045  -8.326 <0.0001
##  time5 - time6    -8.86 2.27 1045  -3.912  0.0014
##
## Chronotype = 2:
##  contrast      estimate   SE   df t.ratio p.value
##  time1 - time2   -19.94 2.58 1045  -7.737 <0.0001
##  time1 - time3   -31.71 2.58 1045 -12.301 <0.0001
##  time1 - time4   -39.26 2.58 1045 -15.234 <0.0001
##  time1 - time5   -49.06 2.58 1045 -19.034 <0.0001
##  time1 - time6   -59.21 2.58 1045 -22.971 <0.0001
##  time2 - time3   -11.76 2.58 1045  -4.564 <0.0001
##  time2 - time4   -19.32 2.58 1045  -7.497 <0.0001
##  time2 - time5   -29.12 2.58 1045 -11.297 <0.0001
##  time2 - time6   -39.26 2.58 1045 -15.234 <0.0001
##  time3 - time4    -7.56 2.58 1045  -2.933  0.0401
##  time3 - time5   -17.35 2.58 1045  -6.733 <0.0001
##  time3 - time6   -27.50 2.58 1045 -10.669 <0.0001
##  time4 - time5    -9.79 2.58 1045  -3.800  0.0021
##  time4 - time6   -19.94 2.58 1045  -7.737 <0.0001
##  time5 - time6   -10.15 2.58 1045  -3.937  0.0012
##
## Chronotype = 3:
##  contrast      estimate   SE   df t.ratio p.value
##  time1 - time2   -24.48 2.01 1045 -12.190 <0.0001
##  time1 - time3   -36.55 2.01 1045 -18.201 <0.0001
##  time1 - time4   -45.05 2.01 1045 -22.433 <0.0001
##  time1 - time5   -55.89 2.01 1045 -27.831 <0.0001
##  time1 - time6   -65.05 2.01 1045 -32.392 <0.0001
##  time2 - time3   -12.07 2.01 1045  -6.011 <0.0001
##  time2 - time4   -20.57 2.01 1045 -10.243 <0.0001
##  time2 - time5   -31.41 2.01 1045 -15.640 <0.0001
##  time2 - time6   -40.57 2.01 1045 -20.202 <0.0001
##  time3 - time4    -8.50 2.01 1045  -4.232  0.0004
##  time3 - time5   -19.34 2.01 1045  -9.630 <0.0001
##  time3 - time6   -28.50 2.01 1045 -14.191 <0.0001
##  time4 - time5   -10.84 2.01 1045  -5.397 <0.0001
##  time4 - time6   -20.00 2.01 1045  -9.959 <0.0001
##  time5 - time6    -9.16 2.01 1045  -4.561 <0.0001
##
## Chronotype = 4:
##  contrast      estimate   SE   df t.ratio p.value
##  time1 - time2   -21.23 2.95 1045  -7.203 <0.0001
##  time1 - time3   -28.35 2.95 1045  -9.617 <0.0001
##  time1 - time4   -37.58 2.95 1045 -12.749 <0.0001
##  time1 - time5   -46.04 2.95 1045 -15.620 <0.0001
##  time1 - time6   -54.31 2.95 1045 -18.426 <0.0001
##  time2 - time3    -7.12 2.95 1045  -2.414  0.1522
##  time2 - time4   -16.35 2.95 1045  -5.546 <0.0001
##  time2 - time5   -24.81 2.95 1045  -8.417 <0.0001
##  time2 - time6   -33.08 2.95 1045 -11.222 <0.0001
##  time3 - time4    -9.23 2.95 1045  -3.132  0.0220
##  time3 - time5   -17.69 2.95 1045  -6.003 <0.0001
##  time3 - time6   -25.96 2.95 1045  -8.808 <0.0001
##  time4 - time5    -8.46 2.95 1045  -2.871  0.0478
##  time4 - time6   -16.73 2.95 1045  -5.676 <0.0001
##  time5 - time6    -8.27 2.95 1045  -2.806  0.0573
##
## Chronotype = 5:
##  contrast      estimate   SE   df t.ratio p.value
##  time1 - time2   -33.70 2.38 1045 -14.182 <0.0001
##  time1 - time3   -42.48 2.38 1045 -17.875 <0.0001
##  time1 - time4   -49.80 2.38 1045 -20.957 <0.0001
##  time1 - time5   -59.90 2.38 1045 -25.207 <0.0001
##  time1 - time6   -70.53 2.38 1045 -29.679 <0.0001
##  time2 - time3    -8.78 2.38 1045  -3.693  0.0032
##  time2 - time4   -16.10 2.38 1045  -6.775 <0.0001
##  time2 - time5   -26.20 2.38 1045 -11.026 <0.0001
##  time2 - time6   -36.83 2.38 1045 -15.497 <0.0001
##  time3 - time4    -7.33 2.38 1045  -3.083  0.0256
##  time3 - time5   -17.43 2.38 1045  -7.333 <0.0001
##  time3 - time6   -28.05 2.38 1045 -11.804 <0.0001
##  time4 - time5   -10.10 2.38 1045  -4.250  0.0003
##  time4 - time6   -20.73 2.38 1045  -8.722 <0.0001
##  time5 - time6   -10.62 2.38 1045  -4.471  0.0001
##
## Results are averaged over the levels of: condition
## Degrees-of-freedom method: kenward-roger
## P value adjustment: tukey method for comparing a family of 6 estimates
```

### Karolinska sleepiness scale

#### Model

Data_Chronotype_KSS_Pivot$Chronotype <- as.factor(Data_Chronotype_KSS_Pivot$Chronotype)

Data_Chronotype_KSS_Pivot$value <- factor(Data_Chronotype_KSS_Pivot$value, ordered = TRUE)

Data_Chronotype_KSS_Pivot$time <- as.factor(Data_Chronotype_KSS_Pivot$time)

Data_Chronotype_KSS_Pivot$condition <- as.factor(Data_Chronotype_KSS_Pivot$condition)

ordinalmodel_Chronotype_KSS <- clm(value ~ condition*time*Chronotype + CCQ_z[, 1] + Leeftijd, data = Data_Chronotype_KSS_Pivot)

summary(ordinalmodel_Chronotype_KSS)

```
## formula: value ~ condition * time * Chronotype + CCQ_z[, 1] + Leeftijd
## data:    Data_Chronotype_KSS_Pivot
##
##  link  threshold nobs logLik  AIC     niter max.grad cond.H
##  logit flexible  400  -681.85 1421.70 7(0)  3.91e-08 1.1e+06
##
## Coefficients:
##                               Estimate Std. Error z value Pr(>|z|)
## conditionMF                    0.45237    0.56688   0.798   0.4249
## time2                          0.65395    0.57941   1.129   0.2590
## Chronotype2                    0.98473    0.60382   1.631   0.1029
## Chronotype3                    0.51736    0.52761   0.981   0.3268
## Chronotype4                    0.34042    0.66660   0.511   0.6096
## Chronotype5                   -1.32140    0.58872  -2.245   0.0248 *
## CCQ_z[, 1]                     0.06401    0.10035   0.638   0.5236
## Leeftijd                      -0.02244    0.01182  -1.899   0.0575 .
## conditionMF:time2              1.28870    0.79628   1.618   0.1056
## conditionMF:Chronotype2       -0.07960    0.82731  -0.096   0.9233
## conditionMF:Chronotype3       -0.10906    0.73424  -0.149   0.8819
## conditionMF:Chronotype4        0.43646    0.90640   0.482   0.6301
## conditionMF:Chronotype5        0.77043    0.80895   0.952   0.3409
## time2:Chronotype2              0.17940    0.84280   0.213   0.8314
## time2:Chronotype3              0.36718    0.73663   0.498   0.6182
## time2:Chronotype4              0.14879    0.89552   0.166   0.8680
## time2:Chronotype5              0.88735    0.82187   1.080   0.2803
## conditionMF:time2:Chronotype2 -0.41731    1.19046  -0.351   0.7259
## conditionMF:time2:Chronotype3 -0.51919    1.03556  -0.501   0.6161
## conditionMF:time2:Chronotype4 -0.77408    1.23662  -0.626   0.5313
## conditionMF:time2:Chronotype5 -1.16364    1.13447  -1.026   0.3050
## ---
## Signif. codes:  0 '***' 0.001 '**' 0.01 '*' 0.05 '.' 0.1 ' ' 1
##
## Threshold coefficients:
##     Estimate Std. Error z value
## 1|2  -6.2023     1.1626  -5.335
## 2|3  -2.8879     0.6311  -4.576
## 3|4  -0.7684     0.6026  -1.275
## 4|5   0.5358     0.5994   0.894
## 5|6   1.1217     0.6012   1.866
## 6|7   2.2619     0.6090   3.714
## 7|8   3.4561     0.6299   5.487
## 8|9   5.7782     0.8339   6.929
```

The model showed no influence of age or caffeine consumption. An additional model was therefore constructed.

ordinalmodel_Chronotype_KSS_2 <- clm(value ~ condition*time*Chronotype, data = Data_Chronotype_KSS_Pivot)

summarykss1 <- drop1(ordinalmodel_Chronotype_KSS_2, test = "Chisq")

summary(ordinalmodel_Chronotype_KSS_2)

```
## formula: value ~ condition * time * Chronotype
## data:    Data_Chronotype_KSS_Pivot
##
##  link  threshold nobs logLik  AIC     niter max.grad cond.H
##  logit flexible  400  -683.67 1421.33 7(0)  1.11e-08 2.5e+03
##
## Coefficients:
##                               Estimate Std. Error z value Pr(>|z|)
## conditionMF                    0.43603    0.56515   0.772   0.4404
## time2                          0.64848    0.57741   1.123   0.2614
## Chronotype2                    1.13032    0.60267   1.876   0.0607 .
## Chronotype3                    0.63224    0.52269   1.210   0.2264
## Chronotype4                    0.42722    0.66175   0.646   0.5185
## Chronotype5                   -1.21588    0.58170  -2.090   0.0366 *
## conditionMF:time2              1.29923    0.79411   1.636   0.1018
## conditionMF:Chronotype2       -0.07533    0.83151  -0.091   0.9278
## conditionMF:Chronotype3       -0.07563    0.73242  -0.103   0.9178
## conditionMF:Chronotype4        0.47133    0.90366   0.522   0.6020
## conditionMF:Chronotype5        0.76835    0.80721   0.952   0.3412
## time2:Chronotype2              0.14402    0.84701   0.170   0.8650
## time2:Chronotype3              0.38195    0.73479   0.520   0.6032
## time2:Chronotype4              0.15806    0.89221   0.177   0.8594
## time2:Chronotype5              0.89685    0.81870   1.095   0.2733
## conditionMF:time2:Chronotype2 -0.38538    1.19813  -0.322   0.7477
## conditionMF:time2:Chronotype3 -0.52728    1.03213  -0.511   0.6094
## conditionMF:time2:Chronotype4 -0.79012    1.23195  -0.641   0.5213
## conditionMF:time2:Chronotype5 -1.18529    1.13342  -1.046   0.2957
## ---
## Signif. codes:  0 '***' 0.001 '**' 0.01 '*' 0.05 '.' 0.1 ' ' 1
##
## Threshold coefficients:
##     Estimate Std. Error z value
## 1|2 -5.37555    1.07638  -4.994
## 2|3 -2.05302    0.44860  -4.576
## 3|4  0.06117    0.41337   0.148
## 4|5  1.35028    0.41879   3.224
## 5|6  1.93288    0.42368   4.562
## 6|7  3.06862    0.43819   7.003
## 7|8  4.25876    0.46964   9.068
## 8|9  6.57837    0.72163   9.116
print(summarykss1)
## Single term deletions
##
## Model:
## value ~ condition * time * Chronotype
##                           Df    AIC    LRT Pr(>Chi)
## <none>                       1421.3
## condition:time:Chronotype  4 1414.5 1.1949   0.8789
confint(ordinalmodel_Chronotype_KSS_2)
##                                     2.5 %     97.5 %
## conditionMF                   -0.66977420  1.5515467
## time2                         -0.48328284  1.7855582
## Chronotype2                   -0.04929498  2.3196782
## Chronotype3                   -0.38815462  1.6662653
## Chronotype4                   -0.87101711  1.7329759
## Chronotype5                   -2.36225919 -0.0761974
## conditionMF:time2             -0.25572654  2.8613797
## conditionMF:Chronotype2       -1.70964593  1.5551406
## conditionMF:Chronotype3       -1.51551289  1.3597884
## conditionMF:Chronotype4       -1.30469625  2.2449644
## conditionMF:Chronotype5       -0.81503752  2.3545016
## time2:Chronotype2             -1.51478718  1.8110361
## time2:Chronotype3             -1.05890574  1.8254887
## time2:Chronotype4             -1.59338328  1.9113914
## time2:Chronotype5             -0.70524973  2.5090356
## conditionMF:time2:Chronotype2 -2.73966763  1.9616234
## conditionMF:time2:Chronotype3 -2.55354377  1.4956330
## conditionMF:time2:Chronotype4 -3.20932925  1.6254061
## conditionMF:time2:Chronotype5 -3.41208962  1.0350295
ordinalmodel_Chronotype_KSS_3 <- clm(value ~ condition*Chronotype, data = Data_Chronotype_KSS_Pivot)
```

summarykss2 <- drop1(ordinalmodel_Chronotype_KSS_3, test = "Chisq")

summary(ordinalmodel_Chronotype_KSS_3)

```
## formula: value ~ condition * Chronotype
## data:    Data_Chronotype_KSS_Pivot
##
##  link  threshold nobs logLik  AIC     niter max.grad cond.H
##  logit flexible  400  -713.45 1460.91 7(0)  9.08e-09 7.4e+02
##
## Coefficients:
##                         Estimate Std. Error z value Pr(>|z|)
## conditionMF              1.03867    0.39379   2.638  0.00835 **
## Chronotype2              1.12860    0.41537   2.717  0.00659 **
## Chronotype3              0.77940    0.36190   2.154  0.03127 *
## Chronotype4              0.54081    0.43627   1.240  0.21512
## Chronotype5             -0.71739    0.40631  -1.766  0.07746 .
## conditionMF:Chronotype2 -0.43588    0.59050  -0.738  0.46042
## conditionMF:Chronotype3 -0.37168    0.51136  -0.727  0.46732
## conditionMF:Chronotype4 -0.01138    0.60917  -0.019  0.98510
## conditionMF:Chronotype5  0.14386    0.56374   0.255  0.79858
## ---
## Signif. codes:  0 '***' 0.001 '**' 0.01 '*' 0.05 '.' 0.1 ' ' 1
##
## Threshold coefficients:
##     Estimate Std. Error z value
## 1|2  -5.4909     1.0340  -5.311
## 2|3  -2.2115     0.3349  -6.604
## 3|4  -0.2119     0.2875  -0.737
## 4|5   0.9638     0.2905   3.318
## 5|6   1.4903     0.2944   5.062
## 6|7   2.5311     0.3104   8.155
## 7|8   3.6316     0.3486  10.417
## 8|9   5.8754     0.6462   9.092
print(summarykss2)
## Single term deletions
##
## Model:
## value ~ condition * Chronotype
##                      Df    AIC    LRT Pr(>Chi)
## <none>                  1460.9
## condition:Chronotype  4 1454.5 1.6209    0.805
ordinalmodel_Chronotype_KSS_4 <- clm(value ~ time*Chronotype, data = Data_Chronotype_KSS_Pivot)
```

summarykss3 <- drop1(ordinalmodel_Chronotype_KSS_4, test = "Chisq")

summary(ordinalmodel_Chronotype_KSS_4)

```
## formula: value ~ time * Chronotype
## data:    Data_Chronotype_KSS_Pivot
##
##  link  threshold nobs logLik  AIC     niter max.grad cond.H
##  logit flexible  400  -701.47 1436.94 7(0)  9.91e-09 6.8e+02
##
## Coefficients:
##                   Estimate Std. Error z value Pr(>|z|)
## time2              1.26382    0.39386   3.209  0.00133 **
## Chronotype2        1.03185    0.41241   2.502  0.01235 *
## Chronotype3        0.55248    0.36155   1.528  0.12650
## Chronotype4        0.65781    0.44785   1.469  0.14188
## Chronotype5       -0.81590    0.40446  -2.017  0.04367 *
## time2:Chronotype2 -0.14471    0.59635  -0.243  0.80826
## time2:Chronotype3  0.06383    0.50979   0.125  0.90036
## time2:Chronotype4 -0.30781    0.61465  -0.501  0.61652
## time2:Chronotype5  0.30044    0.56538   0.531  0.59514
## ---
## Signif. codes:  0 '***' 0.001 '**' 0.01 '*' 0.05 '.' 0.1 ' ' 1
##
## Threshold coefficients:
##     Estimate Std. Error z value
## 1|2  -5.4766     1.0338  -5.298
## 2|3  -2.1743     0.3324  -6.541
## 3|4  -0.1467     0.2825  -0.519
## 4|5   1.0803     0.2882   3.749
## 5|6   1.6249     0.2939   5.529
## 6|7   2.6760     0.3104   8.620
## 7|8   3.8020     0.3494  10.881
## 8|9   6.0814     0.6476   9.391
print(summarykss3)
## Single term deletions
##
## Model:
## value ~ time * Chronotype
##                 Df    AIC    LRT Pr(>Chi)
## <none>             1436.9
## time:Chronotype  4 1430.0 1.0911   0.8957
ordinalmodel_Chronotype_KSS_5 <- clm(value ~ Chronotype, data = Data_Chronotype_KSS_Pivot)
```

summarykss4 <- drop1(ordinalmodel_Chronotype_KSS_5, test = "Chisq")

summary(ordinalmodel_Chronotype_KSS_5)

```
## formula: value ~ Chronotype
## data:    Data_Chronotype_KSS_Pivot
##
##  link  threshold nobs logLik  AIC     niter max.grad cond.H
##  logit flexible  400  -726.26 1476.52 7(0)  8.25e-09 5.0e+02
##
## Coefficients:
##             Estimate Std. Error z value Pr(>|z|)
## Chronotype2   0.8506     0.2943   2.891  0.00384 **
## Chronotype3   0.5439     0.2538   2.143  0.03213 *
## Chronotype4   0.5057     0.3052   1.657  0.09752 .
## Chronotype5  -0.6231     0.2828  -2.203  0.02757 *
## ---
## Signif. codes:  0 '***' 0.001 '**' 0.01 '*' 0.05 '.' 0.1 ' ' 1
##
## Threshold coefficients:
##     Estimate Std. Error z value
## 1|2  -5.8881     1.0160  -5.796
## 2|3  -2.6259     0.2720  -9.655
## 3|4  -0.7071     0.2038  -3.470
## 4|5   0.4186     0.2010   2.083
## 5|6   0.9215     0.2045   4.506
## 6|7   1.9201     0.2224   8.633
## 7|8   3.0004     0.2712  11.065
## 8|9   5.2355     0.6075   8.618
print(summarykss4)
## Single term deletions
##
## Model:
## value ~ Chronotype
##            Df    AIC    LRT  Pr(>Chi)
## <none>        1476.5
## Chronotype  4 1499.5 30.994 3.071e-06 ***
## ---
## Signif. codes:  0 '***' 0.001 '**' 0.01 '*' 0.05 '.' 0.1 ' ' 1
exp(coef(ordinalmodel_Chronotype_KSS_2))
##                           1|2                           2|3
##                    0.00462839                    0.12834675
##                           3|4                           4|5
##                    1.06307537                    3.85850082
##                           5|6                           6|7
##                    6.90935232                   21.51229638
##                           7|8                           8|9
##                   70.72188117                  719.36656211
##                   conditionMF                         time2
##                    1.54654919                    1.91263755
##                   Chronotype2                   Chronotype3
##                    3.09665776                    1.88183009
##                   Chronotype4                   Chronotype5
##                    1.53298876                    0.29644850
##             conditionMF:time2       conditionMF:Chronotype2
##                    3.66647970                    0.92744182
##       conditionMF:Chronotype3       conditionMF:Chronotype4
##                    0.92715499                    1.60212775
##       conditionMF:Chronotype5             time2:Chronotype2
##                    2.15619770                    1.15491086
##             time2:Chronotype3             time2:Chronotype4
##                    1.46514356                    1.17124033
##             time2:Chronotype5 conditionMF:time2:Chronotype2
##                    2.45185681                    0.68019496
## conditionMF:time2:Chronotype3 conditionMF:time2:Chronotype4
##                    0.59020923                    0.45379015
## conditionMF:time2:Chronotype5
##                    0.30565689
```

#### Post Hoc

```
pairs(emmeans(ordinalmodel_Chronotype_KSS_2, ~ Chronotype))
## NOTE: Results may be misleading due to involvement in interactions
##  contrast                  estimate    SE  df z.ratio p.value
##  Chronotype1 - Chronotype2   -1.068 0.303 Inf  -3.530  0.0038
##  Chronotype1 - Chronotype3   -0.654 0.259 Inf  -2.521  0.0861
##  Chronotype1 - Chronotype4   -0.544 0.309 Inf  -1.764  0.3948
##  Chronotype1 - Chronotype5    0.680 0.285 Inf   2.386  0.1192
##  Chronotype2 - Chronotype3    0.415 0.280 Inf   1.483  0.5736
##  Chronotype2 - Chronotype4    0.524 0.326 Inf   1.606  0.4933
##  Chronotype2 - Chronotype5    1.748 0.312 Inf   5.605 <0.0001
##  Chronotype3 - Chronotype4    0.109 0.288 Inf   0.379  0.9956
##  Chronotype3 - Chronotype5    1.333 0.268 Inf   4.973 <0.0001
##  Chronotype4 - Chronotype5    1.224 0.316 Inf   3.876  0.0010
##
## Results are averaged over the levels of: condition, time
## P value adjustment: tukey method for comparing a family of 5 estimates
```

## Difference in Mental Fatigue variables between PSQI groups

### Subjective feeling of mental fatigue

#### Outliers

outliersMVASPSQI <- lapply(Data_PSQI_MVASMC[, -1], function(col) {

detect_outliers_z(col, Data_PSQI_MVASMC[, 1])

})

outliersMVASPSQI

```
## $PSQI
## NULL
##
## $CCQ
## NULL
##
## $MVAS_MF_2
## NULL
##
## $MVAS_MF_5
## NULL
##
## $MVAS_CON_2
## NULL
##
## $MVAS_CON_5
## [1] "MF_INDIFF_V1_278"
##
## $CCQ_z
## NULL
##
## $PSQI_group
## NULL
```

#### Normality

shapiro_results_PSQI_MVAS <- lapply(Data_PSQI_MVASMC, shapiro_test)

shapiro_results_PSQI_MVAS

```
## $PP_number
## [1] NA
##
## $PSQI
## [1] 0.0002765065
##
## $CCQ
## [1] 5.021543e-05
##
## $MVAS_MF_2
## [1] 0.01862963
##
## $MVAS_MF_5
## [1] 0.06245681
##
## $MVAS_CON_2
## [1] 0.0004747385
##
## $MVAS_CON_5
## [1] 0.003220264
##
## $CCQ_z
## [1] 5.021543e-05
##
## $PSQI_group
## [1] 5.96751e-09
```

Results of the MVAS were not normally distributed. We therefore chose to perform a square root transformation, as with the original data analysis. This led to a normalized distribution.

sqrtMVAS_PSQI <- Data_PSQI_MVASMC

sqrtMVAS_PSQI[, -c(1:3, 8:9)] <- sqrt(Data_PSQI_MVASMC[, -c(1:3, 8:9)])

Data_PSQIgroups_sqrtMVAS_Pivot <- sqrtMVAS_PSQI %>%

pivot_longer(

cols = starts_with("MVAS"),

names_to = c("condition", "time"),

names_pattern = "MVAS_(.*)_(.*)",

values_to = "value"

) %>%

mutate(time = case_when(

time == "2" ~ "1",

time == "5" ~ "2",

TRUE ~ time

))

shapiro_results_PSQI_sqrtMVAS <- lapply(sqrtMVAS_PSQI, shapiro_test)

shapiro_results_PSQI_sqrtMVAS

```
## $PP_number
## [1] NA
##
## $PSQI
## [1] 0.0002765065
##
## $CCQ
## [1] 5.021543e-05
##
## $MVAS_MF_2
## [1] 0.569261
##
## $MVAS_MF_5
## [1] 0.01733542
##
## $MVAS_CON_2
## [1] 0.1873001
##
## $MVAS_CON_5
## [1] 0.5307133
##
## $CCQ_z
## [1] 5.021543e-05
##
## $PSQI_group
## [1] 5.96751e-09
sqrtMVAS_PSQI_cont <- Data_PSQI_MVASMC_cont
```

sqrtMVAS_PSQI_cont[, -c(1:3, 8)] <- sqrt(Data_PSQI_MVASMC_cont[, -c(1:3, 8)])

Data_PSQIcont_sqrtMVAS_Pivot <- sqrtMVAS_PSQI_cont %>%

pivot_longer(

cols = starts_with("MVAS"),

names_to = c("condition", "time"),

names_pattern = "MVAS_(.*)_(.*)",

values_to = "value"

) %>%

mutate(time = case_when(

time == "2" ~ "1",

time == "5" ~ "2",

TRUE ~ time

))

#### Models

Data_PSQIgroups_sqrtMVAS_Pivot$PSQI_group <- as.factor(Data_PSQIgroups_sqrtMVAS_Pivot$PSQI_group)

Data_PSQIgroups_sqrtMVAS_Pivot$condition <- as.factor(Data_PSQIgroups_sqrtMVAS_Pivot$condition)

Data_PSQIgroups_sqrtMVAS_Pivot$time <- as.factor(Data_PSQIgroups_sqrtMVAS_Pivot$time)

model_PSQI_sqrtMVAS <- lmer(value ~ condition*time*PSQI_group + CCQ_z[, 1] + (1 | PP_number), data = Data_PSQIgroups_sqrtMVAS_Pivot)

summary(model_PSQI_sqrtMVAS)

```
## Linear mixed model fit by REML. t-tests use Satterthwaite's method [
## lmerModLmerTest]
## Formula: value ~ condition * time * PSQI_group + CCQ_z[, 1] + (1 | PP_number)
##    Data: Data_PSQIgroups_sqrtMVAS_Pivot
##
## REML criterion at convergence: 639.6
##
## Scaled residuals:
##      Min       1Q   Median       3Q      Max
## -2.42871 -0.53217 -0.08308  0.53439  2.58723
##
## Random effects:
##  Groups    Name        Variance Std.Dev.
##  PP_number (Intercept) 0.8249   0.9082
##  Residual              2.2234   1.4911
## Number of obs: 167, groups:  PP_number, 42
##
## Fixed effects:
##                                Estimate Std. Error        df t value Pr(>|t|)
## (Intercept)                     4.10616    0.38315 126.75310  10.717  < 2e-16
## conditionMF                    -0.04070    0.46016 118.88382  -0.088    0.930
## time2                           0.60156    0.46016 118.88382   1.307    0.194
## PSQI_group2                     0.88064    0.54161 126.86474   1.626    0.106
## CCQ_z[, 1]                     -0.01906    0.16308  38.65476  -0.117    0.908
## conditionMF:time2               3.17572    0.65553 119.29340   4.845 3.85e-06
## conditionMF:PSQI_group2         0.37683    0.65077 118.88382   0.579    0.564
## time2:PSQI_group2              -0.39619    0.65077 118.88382  -0.609    0.544
## conditionMF:time2:PSQI_group2  -0.23521    0.92370 119.09011  -0.255    0.799
##
## (Intercept)                   ***
## conditionMF
## time2
## PSQI_group2
## CCQ_z[, 1]
## conditionMF:time2             ***
## conditionMF:PSQI_group2
## time2:PSQI_group2
## conditionMF:time2:PSQI_group2
## ---
## Signif. codes:  0 '***' 0.001 '**' 0.01 '*' 0.05 '.' 0.1 ' ' 1
##
## Correlation of Fixed Effects:
##             (Intr) cndtMF time2  PSQI_2 CCQ_[1 cnMF:2 cMF:PS t2:PSQ
## conditionMF -0.601
## time2       -0.601  0.500
## PSQI_group2 -0.710  0.425  0.425
## CCQ_z[, 1]  -0.106  0.000  0.000  0.102
## cndtnMF:tm2  0.422 -0.702 -0.702 -0.298 -0.001
## cnMF:PSQI_2  0.425 -0.707 -0.354 -0.601  0.000  0.496
## tm2:PSQI_g2  0.425 -0.354 -0.707 -0.601  0.000  0.496  0.500
## cMF:2:PSQI_ -0.299  0.498  0.498  0.423  0.001 -0.710 -0.705 -0.705
```

The model showed no influence of caffeine consumption. An additional model was therefore constructed.

model_PSQI_sqrtMVAS_2 <- lmer(value ~ condition*time*PSQI_group + (1 | PP_number), data = Data_PSQIgroups_sqrtMVAS_Pivot)

anova_PSQI_sqrtMVAS <- anova(model_PSQI_sqrtMVAS_2)

effectsize_PSQI_sqrtMVAS <- eta_squared(model_PSQI_sqrtMVAS_2, partial = TRUE)

summary(model_PSQI_sqrtMVAS_2)

```
## Linear mixed model fit by REML. t-tests use Satterthwaite's method [
## lmerModLmerTest]
## Formula: value ~ condition * time * PSQI_group + (1 | PP_number)
##    Data: Data_PSQIgroups_sqrtMVAS_Pivot
##
## REML criterion at convergence: 637.8
##
## Scaled residuals:
##      Min       1Q   Median       3Q      Max
## -2.41548 -0.53332 -0.08831  0.52469  2.60032
##
## Random effects:
##  Groups    Name        Variance Std.Dev.
##  PP_number (Intercept) 0.7905   0.8891
##  Residual              2.2235   1.4911
## Number of obs: 167, groups:  PP_number, 42
##
## Fixed effects:
##                               Estimate Std. Error       df t value Pr(>|t|)
## (Intercept)                     4.1014     0.3788 131.8505  10.826  < 2e-16 ***
## conditionMF                    -0.0407     0.4602 118.8726  -0.088    0.930
## time2                           0.6016     0.4602 118.8726   1.307    0.194
## PSQI_group2                     0.8871     0.5358 131.8505   1.656    0.100
## conditionMF:time2               3.1748     0.6555 119.2905   4.843 3.88e-06 ***
## conditionMF:PSQI_group2         0.3768     0.6508 118.8726   0.579    0.564
## time2:PSQI_group2              -0.3962     0.6508 118.8726  -0.609    0.544
## conditionMF:time2:PSQI_group2  -0.2343     0.9237 119.0830  -0.254    0.800
## ---
## Signif. codes:  0 '***' 0.001 '**' 0.01 '*' 0.05 '.' 0.1 ' ' 1
##
## Correlation of Fixed Effects:
##             (Intr) cndtMF time2  PSQI_2 cnMF:2 cMF:PS t2:PSQ
## conditionMF -0.607
## time2       -0.607  0.500
## PSQI_group2 -0.707  0.429  0.429
## cndtnMF:tm2  0.426 -0.702 -0.702 -0.301
## cnMF:PSQI_2  0.429 -0.707 -0.354 -0.607  0.496
## tm2:PSQI_g2  0.429 -0.354 -0.707 -0.607  0.496  0.500
## cMF:2:PSQI_ -0.303  0.498  0.498  0.428 -0.710 -0.705 -0.705
print(anova_PSQI_sqrtMVAS)
## Type III Analysis of Variance Table with Satterthwaite's method
##                            Sum Sq Mean Sq NumDF   DenDF F value    Pr(>F)
## condition                 117.195 117.195     1 119.083 52.7078 4.324e-11 ***
## time                      155.677 155.677     1 119.083 70.0149 1.303e-13 ***
## PSQI_group                 11.592  11.592     1  39.843  5.2133   0.02783 *
## condition:time             97.454  97.454     1 119.083 43.8293 1.084e-09 ***
## condition:PSQI_group        0.703   0.703     1 119.083  0.3161   0.57499
## time:PSQI_group             2.747   2.747     1 119.083  1.2353   0.26861
## condition:time:PSQI_group   0.143   0.143     1 119.083  0.0643   0.80021
## ---
## Signif. codes:  0 '***' 0.001 '**' 0.01 '*' 0.05 '.' 0.1 ' ' 1
print(effectsize_PSQI_sqrtMVAS)
## # Effect Size for ANOVA (Type III)
##
## Parameter                 | Eta2 (partial) |       95% CI
## ---------------------------------------------------------
## condition                 |           0.31 | [0.20, 1.00]
## time                      |           0.37 | [0.26, 1.00]
## PSQI_group                |           0.12 | [0.01, 1.00]
## condition:time            |           0.27 | [0.16, 1.00]
## condition:PSQI_group      |       2.65e-03 | [0.00, 1.00]
## time:PSQI_group           |           0.01 | [0.00, 1.00]
## condition:time:PSQI_group |       5.40e-04 | [0.00, 1.00]
##
## - One-sided CIs: upper bound fixed at [1.00].
confint(model_PSQI_sqrtMVAS_2)
## Computing profile confidence intervals ...
##                                    2.5 %    97.5 %
## .sig01                         0.5472533 1.2307286
## .sigma                         1.2915727 1.6559040
## (Intercept)                    3.3717988 4.8310351
## conditionMF                   -0.9275509 0.8461416
## time2                         -0.2852900 1.4884026
## PSQI_group2                   -0.1447578 1.9189141
## conditionMF:time2              1.9103629 4.4372030
## conditionMF:PSQI_group2       -0.8773566 1.6310235
## time2:PSQI_group2             -1.6503809 0.8579992
## conditionMF:time2:PSQI_group2 -2.0134957 1.5469665
Data_PSQIcont_sqrtMVAS_Pivot$condition <- as.factor(Data_PSQIcont_sqrtMVAS_Pivot$condition)
```

Data_PSQIcont_sqrtMVAS_Pivot$time <- as.factor(Data_PSQIcont_sqrtMVAS_Pivot$time)

model_PSQIcont_sqrtMVAS <- lmer(value ~ condition*time*PSQI + (1 | PP_number), data = Data_PSQIcont_sqrtMVAS_Pivot)

anova_PSQIcont_sqrtMVAS <- anova(model_PSQIcont_sqrtMVAS)

summary(model_PSQIcont_sqrtMVAS )

```
## Linear mixed model fit by REML. t-tests use Satterthwaite's method [
## lmerModLmerTest]
## Formula: value ~ condition * time * PSQI + (1 | PP_number)
##    Data: Data_PSQIcont_sqrtMVAS_Pivot
##
## REML criterion at convergence: 1559
##
## Scaled residuals:
##      Min       1Q   Median       3Q      Max
## -3.01216 -0.56646  0.00215  0.55559  2.79619
##
## Random effects:
##  Groups    Name        Variance Std.Dev.
##  PP_number (Intercept) 0.8621   0.9285
##  Residual              2.0408   1.4286
## Number of obs: 407, groups:  PP_number, 102
##
## Fixed effects:
##                          Estimate Std. Error         df t value Pr(>|t|)
## (Intercept)              4.023482   0.359472 315.631760  11.193  < 2e-16 ***
## conditionMF             -0.198470   0.426253 298.929191  -0.466   0.6418
## time2                    0.527413   0.426253 298.929191   1.237   0.2169
## PSQI                     0.132305   0.076183 315.631760   1.737   0.0834 .
## conditionMF:time2        3.499484   0.604399 299.275414   5.790 1.78e-08 ***
## conditionMF:PSQI         0.030483   0.090336 298.929191   0.337   0.7360
## time2:PSQI              -0.059251   0.090336 298.929191  -0.656   0.5124
## conditionMF:time2:PSQI  -0.009062   0.127935 299.116096  -0.071   0.9436
## ---
## Signif. codes:  0 '***' 0.001 '**' 0.01 '*' 0.05 '.' 0.1 ' ' 1
##
## Correlation of Fixed Effects:
##             (Intr) cndtMF time2  PSQI   cnMF:2 cMF:PS t2:PSQ
## conditionMF -0.593
## time2       -0.593  0.500
## PSQI        -0.883  0.524  0.524
## cndtnMF:tm2  0.418 -0.705 -0.705 -0.369
## cndtMF:PSQI  0.524 -0.883 -0.442 -0.593  0.623
## time2:PSQI   0.524 -0.442 -0.883 -0.593  0.623  0.500
## cnMF:2:PSQI -0.370  0.624  0.624  0.419 -0.883 -0.706 -0.706
print(anova_PSQIcont_sqrtMVAS)
## Type III Analysis of Variance Table with Satterthwaite's method
##                      Sum Sq Mean Sq NumDF   DenDF F value    Pr(>F)
## condition            53.776  53.776     1 299.275 26.3505 5.135e-07 ***
## time                115.878 115.878     1 299.275 56.7804 5.824e-13 ***
## PSQI                  9.939   9.939     1  99.896  4.8702   0.02961 *
## condition:time       68.417  68.417     1 299.275 33.5244 1.778e-08 ***
## condition:PSQI        0.336   0.336     1 299.116  0.1646   0.68525
## time:PSQI             2.029   2.029     1 299.116  0.9942   0.31952
## condition:time:PSQI   0.010   0.010     1 299.116  0.0050   0.94358
## ---
## Signif. codes:  0 '***' 0.001 '**' 0.01 '*' 0.05 '.' 0.1 ' ' 1
```

### Go stimuli

#### Models

Data_PSQIgroups_zscore_transformed$conditie <- factor(Data_PSQIgroups_zscore_transformed$conditie, levels = c("MF", "CON"))

Data_PSQIgroups_zscore_transformed$tijd <- factor(Data_PSQIgroups_zscore_transformed$tijd, levels = c("Pre", "Post"))

Data_PSQIgroups_zscore_transformed$PSQI_group <- factor(Data_PSQIgroups_zscore_transformed$PSQI_group, levels = c("1", "2"))

model_PSQI_zscore <- lmer(z_score[, 1] ~ conditie*tijd*PSQI_group + CCQ_z[, 1] + (1 | PP_number), data = Data_PSQIgroups_zscore_transformed)

summary(model_PSQI_zscore)

```
## Linear mixed model fit by REML. t-tests use Satterthwaite's method [
## lmerModLmerTest]
## Formula: z_score[, 1] ~ conditie * tijd * PSQI_group + CCQ_z[, 1] + (1 |
##     PP_number)
##    Data: Data_PSQIgroups_zscore_transformed
##
## REML criterion at convergence: 291.5
##
## Scaled residuals:
##      Min       1Q   Median       3Q      Max
## -3.04869 -0.44650  0.09073  0.54049  2.03956
##
## Random effects:
##  Groups    Name        Variance Std.Dev.
##  PP_number (Intercept) 0.2516   0.5016
##  Residual              0.1945   0.4410
## Number of obs: 168, groups:  PP_number, 42
##
## Fixed effects:
##                                   Estimate Std. Error        df t value
## (Intercept)                        0.04811    0.14696  78.96931   0.327
## conditieCON                       -0.00777    0.13609 120.00000  -0.057
## tijdPost                          -0.45138    0.13609 120.00000  -3.317
## PSQI_group2                        0.12563    0.20770  79.04589   0.605
## CCQ_z[, 1]                        -0.04390    0.07604  39.00000  -0.577
## conditieCON:tijdPost               0.39542    0.19246 120.00000   2.055
## conditieCON:PSQI_group2            0.21676    0.19246 120.00000   1.126
## tijdPost:PSQI_group2              -0.14363    0.19246 120.00000  -0.746
## conditieCON:tijdPost:PSQI_group2   0.04012    0.27218 120.00000   0.147
##                                  Pr(>|t|)
## (Intercept)                       0.74423
## conditieCON                       0.95457
## tijdPost                          0.00121 **
## PSQI_group2                       0.54699
## CCQ_z[, 1]                        0.56704
## conditieCON:tijdPost              0.04209 *
## conditieCON:PSQI_group2           0.26231
## tijdPost:PSQI_group2              0.45697
## conditieCON:tijdPost:PSQI_group2  0.88307
## ---
## Signif. codes:  0 '***' 0.001 '**' 0.01 '*' 0.05 '.' 0.1 ' ' 1
##
## Correlation of Fixed Effects:
##             (Intr) cndCON tjdPst PSQI_2 CCQ_[1 cnCON:P cCON:PS tP:PSQ
## conditieCON -0.463
## tijdPost    -0.463  0.500
## PSQI_group2 -0.712  0.328  0.328
## CCQ_z[, 1]  -0.129  0.000  0.000  0.124
## cndtCON:tjP  0.327 -0.707 -0.707 -0.232  0.000
## cCON:PSQI_2  0.327 -0.707 -0.354 -0.463  0.000  0.500
## tjdP:PSQI_2  0.327 -0.354 -0.707 -0.463  0.000  0.500   0.500
## cCON:P:PSQI -0.232  0.500  0.500  0.328  0.000 -0.707  -0.707  -0.707
```

The model showed no influence of caffeine consumption. An additional model was therefore constructed.

model_PSQI_zscore_1 <- lmer(z_score[, 1] ~ conditie*tijd*PSQI_group + (1 | PP_number), data = Data_PSQIgroups_zscore_transformed)

anova_PSQI_zscore <- anova(model_PSQI_zscore_1)

effectsize_PSQI_zscore <- eta_squared(model_PSQI_zscore_1, partial = TRUE)

summary(model_PSQI_zscore_1)

```
## Linear mixed model fit by REML. t-tests use Satterthwaite's method [
## lmerModLmerTest]
## Formula: z_score[, 1] ~ conditie * tijd * PSQI_group + (1 | PP_number)
##    Data: Data_PSQIgroups_zscore_transformed
##
## REML criterion at convergence: 288.6
##
## Scaled residuals:
##      Min       1Q   Median       3Q      Max
## -3.03607 -0.43586  0.08837  0.53207  2.03164
##
## Random effects:
##  Groups    Name        Variance Std.Dev.
##  PP_number (Intercept) 0.2466   0.4965
##  Residual              0.1945   0.4410
## Number of obs: 168, groups:  PP_number, 42
##
## Fixed effects:
##                                   Estimate Std. Error        df t value
## (Intercept)                        0.03719    0.14492  82.57624   0.257
## conditieCON                       -0.00777    0.13609 120.00000  -0.057
## tijdPost                          -0.45138    0.13609 120.00000  -3.317
## PSQI_group2                        0.14047    0.20494  82.57624   0.685
## conditieCON:tijdPost               0.39542    0.19246 120.00000   2.055
## conditieCON:PSQI_group2            0.21676    0.19246 120.00000   1.126
## tijdPost:PSQI_group2              -0.14363    0.19246 120.00000  -0.746
## conditieCON:tijdPost:PSQI_group2   0.04012    0.27218 120.00000   0.147
##                                  Pr(>|t|)
## (Intercept)                       0.79811
## conditieCON                       0.95457
## tijdPost                          0.00121 **
## PSQI_group2                       0.49500
## conditieCON:tijdPost              0.04209 *
## conditieCON:PSQI_group2           0.26231
## tijdPost:PSQI_group2              0.45697
## conditieCON:tijdPost:PSQI_group2  0.88307
## ---
## Signif. codes:  0 '***' 0.001 '**' 0.01 '*' 0.05 '.' 0.1 ' ' 1
##
## Correlation of Fixed Effects:
##             (Intr) cndCON tjdPst PSQI_2 cnCON:P cCON:PS tP:PSQ
## conditieCON -0.470
## tijdPost    -0.470  0.500
## PSQI_group2 -0.707  0.332  0.332
## cndtCON:tjP  0.332 -0.707 -0.707 -0.235
## cCON:PSQI_2  0.332 -0.707 -0.354 -0.470  0.500
## tjdP:PSQI_2  0.332 -0.354 -0.707 -0.470  0.500   0.500
## cCON:P:PSQI -0.235  0.500  0.500  0.332 -0.707  -0.707  -0.707
print(anova_PSQI_zscore)
## Type III Analysis of Variance Table with Satterthwaite's method
##                          Sum Sq Mean Sq NumDF DenDF F value    Pr(>F)
## conditie                 3.9934  3.9934     1   120 20.5348 1.394e-05 ***
## tijd                     4.1796  4.1796     1   120 21.4923 9.111e-06 ***
## PSQI_group               0.2421  0.2421     1    40  1.2448  0.271198
## conditie:tijd            1.8126  1.8126     1   120  9.3206  0.002791 **
## conditie:PSQI_group      0.5889  0.5889     1   120  3.0281  0.084397 .
## tijd:PSQI_group          0.1603  0.1603     1   120  0.8244  0.365703
## conditie:tijd:PSQI_group 0.0042  0.0042     1   120  0.0217  0.883074
## ---
## Signif. codes:  0 '***' 0.001 '**' 0.01 '*' 0.05 '.' 0.1 ' ' 1
print(effectsize_PSQI_zscore)
## # Effect Size for ANOVA (Type III)
##
## Parameter                | Eta2 (partial) |       95% CI
## --------------------------------------------------------
## conditie                 |           0.15 | [0.06, 1.00]
## tijd                     |           0.15 | [0.07, 1.00]
## PSQI_group               |           0.03 | [0.00, 1.00]
## conditie:tijd            |           0.07 | [0.02, 1.00]
## conditie:PSQI_group      |           0.02 | [0.00, 1.00]
## tijd:PSQI_group          |       6.82e-03 | [0.00, 1.00]
## conditie:tijd:PSQI_group |       1.81e-04 | [0.00, 1.00]
##
## - One-sided CIs: upper bound fixed at [1.00].
confint(model_PSQI_zscore_1)
## Computing profile confidence intervals ...
##                                        2.5 %     97.5 %
## .sig01                            0.37589816  0.6320666
## .sigma                            0.38223301  0.4894983
## (Intercept)                      -0.24315335  0.3175295
## conditieCON                      -0.27007206  0.2545321
## tijdPost                         -0.71368684 -0.1890827
## PSQI_group2                      -0.25599132  0.5369340
## conditieCON:tijdPost              0.02447218  0.7663745
## conditieCON:PSQI_group2          -0.15419051  0.5877118
## tijdPost:PSQI_group2             -0.51457874  0.2273236
## conditieCON:tijdPost:PSQI_group2 -0.48448816  0.5647202
Data_PSQIgroups_zscore_transformed$conditie <- factor(Data_PSQIgroups_zscore_transformed$conditie, levels = c("MF", "CON"))
```

Data_PSQIgroups_zscore_transformed$tijd <- factor(Data_PSQIgroups_zscore_transformed$tijd, levels = c("Pre", "Post"))

model_PSQIcont_zscore <- lmer(z_score[, 1] ~ conditie*tijd*PSQI + (1 | PP_number), data = Data_PSQIcont_zscore_transformed)

anova_PSQIcont_zscore <- anova(model_PSQIcont_zscore)

summary(model_PSQIcont_zscore)

```
## Linear mixed model fit by REML. t-tests use Satterthwaite's method [
## lmerModLmerTest]
## Formula: z_score[, 1] ~ conditie * tijd * PSQI + (1 | PP_number)
##    Data: Data_PSQIcont_zscore_transformed
##
## REML criterion at convergence: 690.2
##
## Scaled residuals:
##     Min      1Q  Median      3Q     Max
## -3.1456 -0.5245  0.0715  0.5644  2.4853
##
## Random effects:
##  Groups    Name        Variance Std.Dev.
##  PP_number (Intercept) 0.2585   0.5084
##  Residual              0.1839   0.4288
## Number of obs: 408, groups:  PP_number, 102
##
## Fixed effects:
##                           Estimate Std. Error         df t value Pr(>|t|)
## (Intercept)              -0.082295   0.140324 197.600197  -0.586   0.5582
## conditieMF               -0.195225   0.127946 300.000000  -1.526   0.1281
## tijdPre                  -0.057174   0.127946 300.000000  -0.447   0.6553
## PSQI                      0.037135   0.029739 197.600197   1.249   0.2133
## conditieMF:tijdPre        0.370125   0.180942 300.000000   2.046   0.0417 *
## conditieMF:PSQI          -0.052429   0.027115 300.000000  -1.934   0.0541 .
## tijdPre:PSQI              0.033052   0.027115 300.000000   1.219   0.2238
## conditieMF:tijdPre:PSQI   0.001538   0.038347 300.000000   0.040   0.9680
## ---
## Signif. codes:  0 '***' 0.001 '**' 0.01 '*' 0.05 '.' 0.1 ' ' 1
##
## Correlation of Fixed Effects:
##             (Intr) cndtMF tijdPr PSQI   cnMF:P cMF:PS tP:PSQ
## conditieMF  -0.456
## tijdPre     -0.456  0.500
## PSQI        -0.883  0.403  0.403
## cndtMF:tjdP  0.322 -0.707 -0.707 -0.285
## cndtMF:PSQI  0.403 -0.883 -0.442 -0.456  0.624
## tijdPr:PSQI  0.403 -0.442 -0.883 -0.456  0.624  0.500
## cnMF:P:PSQI -0.285  0.624  0.624  0.322 -0.883 -0.707 -0.707
print(anova_PSQIcont_zscore)
## Type III Analysis of Variance Table with Satterthwaite's method
##                     Sum Sq Mean Sq NumDF DenDF F value  Pr(>F)
## conditie           0.00232 0.00232     1   300  0.0126 0.91064
## tijd               0.36742 0.36742     1   300  1.9982 0.15852
## PSQI               0.23399 0.23399     1   100  1.2726 0.26199
## conditie:tijd      0.76937 0.76937     1   300  4.1843 0.04167 *
## conditie:PSQI      1.33480 1.33480     1   300  7.2594 0.00745 **
## tijd:PSQI          0.57214 0.57214     1   300  3.1116 0.07875 .
## conditie:tijd:PSQI 0.00030 0.00030     1   300  0.0016 0.96803
## ---
## Signif. codes:  0 '***' 0.001 '**' 0.01 '*' 0.05 '.' 0.1 ' ' 1
```

### Time trial

#### Outliers

outliersTT<- lapply(Data_PSQI_TT[, -1], function(col) {

detect_outliers_z(col, Data_PSQI_TT[, 1])

})

outliersTT

```
## $PSQI
## NULL
##
## $CCQ
## [1] "MF_INDIFF_V1_238"
##
## $TT_MF_Distance
## NULL
##
## $TT_CON_Distance
## NULL
##
## $CCQ_z
## [1] "MF_INDIFF_V1_238"
##
## $PSQI_group
## NULL
```

#### Normality

shapiro_results_PSQI_TT <- lapply(Data_PSQI_TT, shapiro_test)

shapiro_results_PSQI_TT

```
## $PP_number
## [1] NA
##
## $PSQI
## [1] 0.0002725602
##
## $CCQ
## [1] 4.574406e-05
##
## $TT_MF_Distance
## [1] 0.2159815
##
## $TT_CON_Distance
## [1] 0.09094022
##
## $CCQ_z
## [1] 4.574406e-05
##
## $PSQI_group
## [1] 1.015736e-08
```

#### Models

Data_PSQIgroups_TT_Pivot$PSQI_group <- as.factor(Data_PSQIgroups_TT_Pivot$PSQI_group)

Data_PSQIgroups_TT_Pivot$condition <- as.factor(Data_PSQIgroups_TT_Pivot$condition)

model_PSQI_TT <- lmer(distance ~ condition*PSQI_group + CCQ_z[, 1] + (1 | PP_number), data = Data_PSQIgroups_TT_Pivot)

anova_PSQI_TT <- anova(model_PSQI_TT)

summary(model_PSQI_TT)

```
## Linear mixed model fit by REML. t-tests use Satterthwaite's method [
## lmerModLmerTest]
## Formula: distance ~ condition * PSQI_group + CCQ_z[, 1] + (1 | PP_number)
##    Data: Data_PSQIgroups_TT_Pivot
##
## REML criterion at convergence: 266.3
##
## Scaled residuals:
##      Min       1Q   Median       3Q      Max
## -2.24395 -0.35837 -0.01978  0.38601  1.91544
##
## Random effects:
##  Groups    Name        Variance Std.Dev.
##  PP_number (Intercept) 3.3102   1.8194
##  Residual              0.3971   0.6302
## Number of obs: 80, groups:  PP_number, 40
##
## Fixed effects:
##                         Estimate Std. Error      df t value Pr(>|t|)
## (Intercept)              13.0343     0.4259 41.0611  30.608   <2e-16 ***
## conditionMF              -0.1519     0.1945 38.0000  -0.781   0.4396
## PSQI_group2              -0.4859     0.6200 41.0324  -0.784   0.4377
## CCQ_z[, 1]                0.5238     0.2755 37.0000   1.901   0.0651 .
## conditionMF:PSQI_group2  -0.2870     0.2822 38.0000  -1.017   0.3155
## ---
## Signif. codes:  0 '***' 0.001 '**' 0.01 '*' 0.05 '.' 0.1 ' ' 1
##
## Correlation of Fixed Effects:
##             (Intr) cndtMF PSQI_2 CCQ_[1
## conditionMF -0.228
## PSQI_group2 -0.698  0.157
## CCQ_z[, 1]  -0.163  0.000  0.182
## cnMF:PSQI_2  0.157 -0.689 -0.228  0.000
```

The model showed no influence of caffeine consumption. An additional model was therefore constructed.

model_PSQI_TT_2 <- lmer(distance ~ condition*PSQI_group + (1 | PP_number), data = Data_PSQIgroups_TT_Pivot)

anova_PSQI_TT_2 <- anova(model_PSQI_TT_2)

effectsize_PSQI_TT <- eta_squared(model_PSQI_TT_2, partial = TRUE)

summary(model_PSQI_TT_2)

```
## Linear mixed model fit by REML. t-tests use Satterthwaite's method [
## lmerModLmerTest]
## Formula: distance ~ condition * PSQI_group + (1 | PP_number)
##    Data: Data_PSQIgroups_TT_Pivot
##
## REML criterion at convergence: 269.1
##
## Scaled residuals:
##     Min      1Q  Median      3Q     Max
## -2.2348 -0.3519  0.0305  0.3849  1.9246
##
## Random effects:
##  Groups    Name        Variance Std.Dev.
##  PP_number (Intercept) 3.5516   1.8846
##  Residual              0.3971   0.6302
## Number of obs: 80, groups:  PP_number, 40
##
## Fixed effects:
##                         Estimate Std. Error      df t value Pr(>|t|)
## (Intercept)              13.1662     0.4336 42.0124  30.363   <2e-16 ***
## conditionMF              -0.1519     0.1945 38.0000  -0.781    0.440
## PSQI_group2              -0.7004     0.6292 42.0124  -1.113    0.272
## conditionMF:PSQI_group2  -0.2870     0.2822 38.0000  -1.017    0.315
## ---
## Signif. codes:  0 '***' 0.001 '**' 0.01 '*' 0.05 '.' 0.1 ' ' 1
##
## Correlation of Fixed Effects:
##             (Intr) cndtMF PSQI_2
## conditionMF -0.224
## PSQI_group2 -0.689  0.155
## cnMF:PSQI_2  0.155 -0.689 -0.224
print(anova_PSQI_TT_2)
## Type III Analysis of Variance Table with Satterthwaite's method
##                       Sum Sq Mean Sq NumDF DenDF F value  Pr(>F)
## condition            1.74117 1.74117     1    38  4.3848 0.04299 *
## PSQI_group           0.75225 0.75225     1    38  1.8944 0.17677
## condition:PSQI_group 0.41094 0.41094     1    38  1.0349 0.31545
## ---
## Signif. codes:  0 '***' 0.001 '**' 0.01 '*' 0.05 '.' 0.1 ' ' 1
print(effectsize_PSQI_TT)
## # Effect Size for ANOVA (Type III)
##
## Parameter            | Eta2 (partial) |       95% CI
## ----------------------------------------------------
## condition            |           0.10 | [0.00, 1.00]
## PSQI_group           |           0.05 | [0.00, 1.00]
## condition:PSQI_group |           0.03 | [0.00, 1.00]
##
## - One-sided CIs: upper bound fixed at [1.00].
confint(model_PSQI_TT_2)
## Computing profile confidence intervals ...
##                              2.5 %     97.5 %
## .sig01                   1.4749262  2.3512715
## .sigma                   0.5007420  0.7779736
## (Intercept)             12.3193947 14.0129863
## conditionMF             -0.5325074  0.2286979
## PSQI_group2             -1.9290615  0.5282595
## conditionMF:PSQI_group2 -0.8392790  0.2651938
Data_PSQIcont_TT_Pivot$condition <- as.factor(Data_PSQIcont_TT_Pivot$condition)
```

model_PSQIcont_TT <- lmer(distance ~ condition*PSQI + (1 | PP_number), data = Data_PSQIcont_TT_Pivot)

anova_PSQIcont_TT <- anova(model_PSQIcont_TT)

summary(model_PSQIcont_TT)

```
## Linear mixed model fit by REML. t-tests use Satterthwaite's method [
## lmerModLmerTest]
## Formula: distance ~ condition * PSQI + (1 | PP_number)
##    Data: Data_PSQIcont_TT_Pivot
##
## REML criterion at convergence: 633
##
## Scaled residuals:
##      Min       1Q   Median       3Q      Max
## -2.39550 -0.39655 -0.00783  0.40827  2.38812
##
## Random effects:
##  Groups    Name        Variance Std.Dev.
##  PP_number (Intercept) 2.7429   1.6562
##  Residual              0.4166   0.6454
## Number of obs: 190, groups:  PP_number, 95
##
## Fixed effects:
##                    Estimate Std. Error         df t value Pr(>|t|)
## (Intercept)       13.316102   0.379433 106.062170  35.095   <2e-16 ***
## conditionMF       -0.009954   0.194844  93.000001  -0.051   0.9594
## PSQI              -0.149025   0.081051 106.062170  -1.839   0.0688 .
## conditionMF:PSQI  -0.040652   0.041621  93.000001  -0.977   0.3312
## ---
## Signif. codes:  0 '***' 0.001 '**' 0.01 '*' 0.05 '.' 0.1 ' ' 1
##
## Correlation of Fixed Effects:
##             (Intr) cndtMF PSQI
## conditionMF -0.257
## PSQI        -0.877  0.225
## cndtMF:PSQI  0.225 -0.877 -0.257
print(anova_PSQIcont_TT)
## Type III Analysis of Variance Table with Satterthwaite's method
##                 Sum Sq Mean Sq NumDF DenDF F value  Pr(>F)
## condition      0.00109 0.00109     1    93  0.0026 0.95937
## PSQI           1.94699 1.94699     1    93  4.6739 0.03319 *
## condition:PSQI 0.39741 0.39741     1    93  0.9540 0.33123
## ---
## Signif. codes:  0 '***' 0.001 '**' 0.01 '*' 0.05 '.' 0.1 ' ' 1
```

### Rating of perceived exertion

#### Outliers

outliersRPE<- lapply(Data_PSQI_RPE[, -1], function(col) {

detect_outliers_z(col, Data_PSQI_RPE[, 1])

})

outliersRPE

```
## $PSQI
## NULL
##
## $CCQ
## NULL
##
## $RPE_1_MF
## [1] "MF_INDIFF_V1_257"
##
## $RPE_2_MF
## NULL
##
## $RPE_3_MF
## NULL
##
## $RPE_4_MF
## NULL
##
## $RPE_5_MF
## NULL
##
## $RPE_6_MF
## NULL
##
## $RPE_1_CON
## [1] "MF_INDIFF_V1_038"
##
## $RPE_2_CON
## NULL
##
## $RPE_3_CON
## NULL
##
## $RPE_4_CON
## NULL
##
## $RPE_5_CON
## NULL
##
## $RPE_6_CON
## NULL
##
## $CCQ_z
## NULL
##
## $PSQI_group
## NULL
```

#### Normality

shapiro_results_PSQI_RPE <- lapply(Data_PSQI_RPE, shapiro_test)

shapiro_results_PSQI_RPE

```
## $PP_number
## [1] NA
##
## $PSQI
## [1] 0.0002765065
##
## $CCQ
## [1] 5.021543e-05
##
## $RPE_1_MF
## [1] 0.0002184208
##
## $RPE_2_MF
## [1] 0.3894465
##
## $RPE_3_MF
## [1] 0.1214114
##
## $RPE_4_MF
## [1] 0.01677609
##
## $RPE_5_MF
## [1] 0.002133549
##
## $RPE_6_MF
## [1] 0.02084383
##
## $RPE_1_CON
## [1] 2.05221e-05
##
## $RPE_2_CON
## [1] 0.2601139
##
## $RPE_3_CON
## [1] 0.8184088
##
## $RPE_4_CON
## [1] 0.1941098
##
## $RPE_5_CON
## [1] 0.4311324
##
## $RPE_6_CON
## [1] 0.006431939
##
## $CCQ_z
## [1] 5.021543e-05
##
## $PSQI_group
## [1] 5.96751e-09
```

#### Models

Data_PSQIgroups_RPE_Pivot$PSQI_group <- as.factor(Data_PSQIgroups_RPE_Pivot$PSQI_group)

Data_PSQIgroups_RPE_Pivot$condition<- as.factor(Data_PSQIgroups_RPE_Pivot$condition)

Data_PSQIgroups_RPE_Pivot$time <- as.factor(Data_PSQIgroups_RPE_Pivot$time)

model_PSQI_RPE <- lmer(value ~ condition*time*PSQI_group + CCQ_z[, 1] + (1 | PP_number), data = Data_PSQIgroups_RPE_Pivot)

summary(model_PSQI_RPE)

```
## Linear mixed model fit by REML. t-tests use Satterthwaite's method [
## lmerModLmerTest]
## Formula: value ~ condition * time * PSQI_group + CCQ_z[, 1] + (1 | PP_number)
##    Data: Data_PSQIgroups_RPE_Pivot
##
## REML criterion at convergence: 3788.7
##
## Scaled residuals:
##     Min      1Q  Median      3Q     Max
## -3.0192 -0.5527  0.0465  0.5320  3.4845
##
## Random effects:
##  Groups    Name        Variance Std.Dev.
##  PP_number (Intercept) 103.5    10.18
##  Residual              110.1    10.49
## Number of obs: 504, groups:  PP_number, 42
##
## Fixed effects:
##                               Estimate Std. Error       df t value Pr(>|t|)
## (Intercept)                    12.5932     3.2107 128.2328   3.922 0.000142 ***
## conditionMF                     2.0952     3.2384 440.0000   0.647 0.517972
## time2                          29.5238     3.2384 440.0000   9.117  < 2e-16 ***
## time3                          35.7619     3.2384 440.0000  11.043  < 2e-16 ***
## time4                          45.0000     3.2384 440.0000  13.896  < 2e-16 ***
## time5                          56.6190     3.2384 440.0000  17.484  < 2e-16 ***
## time6                          66.4286     3.2384 440.0000  20.513  < 2e-16 ***
## PSQI_group2                     0.7358     4.5383 128.4305   0.162 0.871458
## CCQ_z[, 1]                      0.4863     1.4734  39.0000   0.330 0.743142
## conditionMF:time2              -1.0476     4.5798 440.0000  -0.229 0.819171
## conditionMF:time3               2.1429     4.5798 440.0000   0.468 0.640093
## conditionMF:time4              -2.6667     4.5798 440.0000  -0.582 0.560685
## conditionMF:time5              -6.5714     4.5798 440.0000  -1.435 0.152034
## conditionMF:time6              -3.6667     4.5798 440.0000  -0.801 0.423785
## conditionMF:PSQI_group2        -2.0000     4.5798 440.0000  -0.437 0.662543
## time2:PSQI_group2              -4.0000     4.5798 440.0000  -0.873 0.382921
## time3:PSQI_group2               0.7143     4.5798 440.0000   0.156 0.876133
## time4:PSQI_group2              -1.4286     4.5798 440.0000  -0.312 0.755242
## time5:PSQI_group2              -2.9524     4.5798 440.0000  -0.645 0.519488
## time6:PSQI_group2              -3.6667     4.5798 440.0000  -0.801 0.423785
## conditionMF:time2:PSQI_group2   1.6667     6.4768 440.0000   0.257 0.797046
## conditionMF:time3:PSQI_group2   0.8571     6.4768 440.0000   0.132 0.894776
## conditionMF:time4:PSQI_group2   7.8571     6.4768 440.0000   1.213 0.225736
## conditionMF:time5:PSQI_group2   9.9524     6.4768 440.0000   1.537 0.125106
## conditionMF:time6:PSQI_group2   6.9524     6.4768 440.0000   1.073 0.283669
## ---
## Signif. codes:  0 '***' 0.001 '**' 0.01 '*' 0.05 '.' 0.1 ' ' 1
##
## Correlation matrix not shown by default, as p = 25 > 12.
## Use print(x, correlation=TRUE)  or
##     vcov(x)        if you need it
```

The model showed no influence of caffeine consumption. An additional model was therefore constructed.

model_PSQI_RPE_2 <- lmer(value ~ condition*time*PSQI_group + (1 | PP_number), data = Data_PSQIgroups_RPE_Pivot)

anova_PSQI_RPE <- anova(model_PSQI_RPE_2)

effectsize_PSQI_RPE <- eta_squared(model_PSQI_RPE_2, partial = TRUE)

summary(model_PSQI_RPE_2)

```
## Linear mixed model fit by REML. t-tests use Satterthwaite's method [
## lmerModLmerTest]
## Formula: value ~ condition * time * PSQI_group + (1 | PP_number)
##    Data: Data_PSQIgroups_RPE_Pivot
##
## REML criterion at convergence: 3791.4
##
## Scaled residuals:
##     Min      1Q  Median      3Q     Max
## -3.0189 -0.5542  0.0488  0.5298  3.4804
##
## Random effects:
##  Groups    Name        Variance Std.Dev.
##  PP_number (Intercept) 101.0    10.05
##  Residual              110.1    10.49
## Number of obs: 504, groups:  PP_number, 42
##
## Fixed effects:
##                               Estimate Std. Error       df t value Pr(>|t|)
## (Intercept)                    12.7143     3.1709 136.4254   4.010 9.96e-05 ***
## conditionMF                     2.0952     3.2384 440.0000   0.647    0.518
## time2                          29.5238     3.2384 440.0000   9.117  < 2e-16 ***
## time3                          35.7619     3.2384 440.0000  11.043  < 2e-16 ***
## time4                          45.0000     3.2384 440.0000  13.896  < 2e-16 ***
## time5                          56.6190     3.2384 440.0000  17.484  < 2e-16 ***
## time6                          66.4286     3.2384 440.0000  20.513  < 2e-16 ***
## PSQI_group2                     0.5714     4.4843 136.4254   0.127    0.899
## conditionMF:time2              -1.0476     4.5798 440.0000  -0.229    0.819
## conditionMF:time3               2.1429     4.5798 440.0000   0.468    0.640
## conditionMF:time4              -2.6667     4.5798 440.0000  -0.582    0.561
## conditionMF:time5              -6.5714     4.5798 440.0000  -1.435    0.152
## conditionMF:time6              -3.6667     4.5798 440.0000  -0.801    0.424
## conditionMF:PSQI_group2        -2.0000     4.5798 440.0000  -0.437    0.663
## time2:PSQI_group2              -4.0000     4.5798 440.0000  -0.873    0.383
## time3:PSQI_group2               0.7143     4.5798 440.0000   0.156    0.876
## time4:PSQI_group2              -1.4286     4.5798 440.0000  -0.312    0.755
## time5:PSQI_group2              -2.9524     4.5798 440.0000  -0.645    0.519
## time6:PSQI_group2              -3.6667     4.5798 440.0000  -0.801    0.424
## conditionMF:time2:PSQI_group2   1.6667     6.4768 440.0000   0.257    0.797
## conditionMF:time3:PSQI_group2   0.8571     6.4768 440.0000   0.132    0.895
## conditionMF:time4:PSQI_group2   7.8571     6.4768 440.0000   1.213    0.226
## conditionMF:time5:PSQI_group2   9.9524     6.4768 440.0000   1.537    0.125
## conditionMF:time6:PSQI_group2   6.9524     6.4768 440.0000   1.073    0.284
## ---
## Signif. codes:  0 '***' 0.001 '**' 0.01 '*' 0.05 '.' 0.1 ' ' 1
##
## Correlation matrix not shown by default, as p = 24 > 12.
## Use print(x, correlation=TRUE)  or
##     vcov(x)        if you need it
print(anova_PSQI_RPE)
## Type III Analysis of Variance Table with Satterthwaite's method
##                           Sum Sq Mean Sq NumDF DenDF  F value Pr(>F)
## condition                    247     247     1   440   2.2453 0.1347
## time                      216179   43236     5   440 392.6371 <2e-16 ***
## PSQI_group                     0       0     1    40   0.0002 0.9893
## condition:time               216      43     5   440   0.3918 0.8545
## condition:PSQI_group         204     204     1   440   1.8566 0.1737
## time:PSQI_group              437      87     5   440   0.7944 0.5541
## condition:time:PSQI_group    465      93     5   440   0.8443 0.5188
## ---
## Signif. codes:  0 '***' 0.001 '**' 0.01 '*' 0.05 '.' 0.1 ' ' 1
print(effectsize_PSQI_RPE)
## # Effect Size for ANOVA (Type III)
##
## Parameter                 | Eta2 (partial) |       95% CI
## ---------------------------------------------------------
## condition                 |       5.08e-03 | [0.00, 1.00]
## time                      |           0.82 | [0.79, 1.00]
## PSQI_group                |       4.54e-06 | [0.00, 1.00]
## condition:time            |       4.43e-03 | [0.00, 1.00]
## condition:PSQI_group      |       4.20e-03 | [0.00, 1.00]
## time:PSQI_group           |       8.95e-03 | [0.00, 1.00]
## condition:time:PSQI_group |       9.50e-03 | [0.00, 1.00]
##
## - One-sided CIs: upper bound fixed at [1.00].
confint(model_PSQI_RPE_2)
## Computing profile confidence intervals ...
##                                    2.5 %    97.5 %
## .sig01                          7.851356 12.554314
## .sigma                          9.614313 10.938282
## (Intercept)                     6.607323 18.821248
## conditionMF                    -4.111858  8.302335
## time2                          23.316713 35.730906
## time3                          29.554808 41.969001
## time4                          38.792903 51.207097
## time5                          50.411951 62.826144
## time6                          60.221475 72.635668
## PSQI_group2                    -8.065120  9.207977
## conditionMF:time2              -9.825779  7.730541
## conditionMF:time3              -6.635303 10.921017
## conditionMF:time4             -11.444827  6.111493
## conditionMF:time5             -15.349589  2.206732
## conditionMF:time6             -12.444827  5.111493
## conditionMF:PSQI_group2       -10.778160  6.778160
## time2:PSQI_group2             -12.778160  4.778160
## time3:PSQI_group2              -8.063874  9.492446
## time4:PSQI_group2             -10.206732  7.349589
## time5:PSQI_group2             -11.730541  5.825779
## time6:PSQI_group2             -12.444827  5.111493
## conditionMF:time2:PSQI_group2 -10.747526 14.080860
## conditionMF:time3:PSQI_group2 -11.557050 13.271336
## conditionMF:time4:PSQI_group2  -4.557050 20.271336
## conditionMF:time5:PSQI_group2  -2.461812 22.366574
## conditionMF:time6:PSQI_group2  -5.461812 19.366574
Data_PSQIcont_RPE_Pivot$condition<- as.factor(Data_PSQIcont_RPE_Pivot$condition)
```

Data_PSQIcont_RPE_Pivot$time <- as.factor(Data_PSQIcont_RPE_Pivot$time)

model_PSQIcont_RPE <- lmer(value ~ condition*time*PSQI + (1 | PP_number), data = Data_PSQIcont_RPE_Pivot)

anova_PSQIcont_RPE <- anova(model_PSQIcont_RPE)

### Karolinska sleepiness scale

#### Models

Data_PSQIgroups_KSS_Pivot$PSQI_group <- as.factor(Data_PSQIgroups_KSS_Pivot$PSQI_group)

Data_PSQIgroups_KSS_Pivot$value <- factor(Data_PSQIgroups_KSS_Pivot$value, ordered = TRUE)

Data_PSQIgroups_KSS_Pivot$condition <- as.factor(Data_PSQIgroups_KSS_Pivot$condition)

Data_PSQIgroups_KSS_Pivot$time <- as.factor(Data_PSQIgroups_KSS_Pivot$time)

ordinalmodel_PSQI_KSS <- clm(value ~ condition*time*PSQI_group + CCQ_z[, 1], data = Data_PSQIgroups_KSS_Pivot)

summary(ordinalmodel_PSQI_KSS)

```
## formula: value ~ condition * time * PSQI_group + CCQ_z[, 1]
## data:    Data_PSQIgroups_KSS_Pivot
##
##  link  threshold nobs logLik  AIC    niter max.grad cond.H
##  logit flexible  168  -291.83 615.66 6(0)  6.19e-08 4.6e+02
##
## Coefficients:
##                               Estimate Std. Error z value Pr(>|z|)
## conditionMF                    0.53301    0.54977   0.970   0.3323
## time2                          1.02680    0.54979   1.868   0.0618 .
## PSQI_group2                    0.94840    0.57247   1.657   0.0976 .
## CCQ_z[, 1]                     0.02946    0.11934   0.247   0.8051
## conditionMF:time2              0.67642    0.76444   0.885   0.3762
## conditionMF:PSQI_group2       -0.04467    0.77602  -0.058   0.9541
## time2:PSQI_group2              0.18819    0.78305   0.240   0.8101
## conditionMF:time2:PSQI_group2  0.25134    1.10424   0.228   0.8199
## ---
## Signif. codes:  0 '***' 0.001 '**' 0.01 '*' 0.05 '.' 0.1 ' ' 1
##
## Threshold coefficients:
##     Estimate Std. Error z value
## 1|2  -4.0769     1.0516  -3.877
## 2|3  -1.5856     0.4515  -3.512
## 3|4   0.4122     0.4078   1.011
## 4|5   1.5911     0.4314   3.688
## 5|6   2.2170     0.4468   4.962
## 6|7   3.4095     0.4832   7.056
## 7|8   4.7478     0.5631   8.432
## 8|9   6.5107     0.8541   7.623
```

The model showed no influence of caffeine consumption. An additional model was therefore constructed.

ordinalmodel_PSQI_KSS_2 <- clm(value ~ condition*time*PSQI_group, data = Data_PSQIgroups_KSS_Pivot)

summaryksspsqi1 <- drop1(ordinalmodel_PSQI_KSS_2, test = "Chisq")

summary(ordinalmodel_PSQI_KSS_2)

```
## formula: value ~ condition * time * PSQI_group
## data:    Data_PSQIgroups_KSS_Pivot
##
##  link  threshold nobs logLik  AIC    niter max.grad cond.H
##  logit flexible  168  -291.86 613.72 6(0)  6.23e-08 4.6e+02
##
## Coefficients:
##                               Estimate Std. Error z value Pr(>|z|)
## conditionMF                     0.5284     0.5494   0.962   0.3361
## time2                           1.0227     0.5496   1.861   0.0628 .
## PSQI_group2                     0.9344     0.5698   1.640   0.1011
## conditionMF:time2               0.6783     0.7646   0.887   0.3750
## conditionMF:PSQI_group2        -0.0409     0.7762  -0.053   0.9580
## time2:PSQI_group2               0.1878     0.7832   0.240   0.8105
## conditionMF:time2:PSQI_group2   0.2557     1.1042   0.232   0.8168
## ---
## Signif. codes:  0 '***' 0.001 '**' 0.01 '*' 0.05 '.' 0.1 ' ' 1
##
## Threshold coefficients:
##     Estimate Std. Error z value
## 1|2  -4.0865     1.0510  -3.888
## 2|3  -1.5971     0.4490  -3.557
## 3|4   0.3980     0.4035   0.986
## 4|5   1.5745     0.4258   3.698
## 5|6   2.2001     0.4411   4.987
## 6|7   3.3945     0.4790   7.087
## 7|8   4.7350     0.5604   8.449
## 8|9   6.4993     0.8529   7.621
print(summaryksspsqi1)
## Single term deletions
##
## Model:
## value ~ condition * time * PSQI_group
##                           Df    AIC      LRT Pr(>Chi)
## <none>                       613.72
## condition:time:PSQI_group  1 611.77 0.053645   0.8168
confint(ordinalmodel_PSQI_KSS_2)
##                                     2.5 %   97.5 %
## conditionMF                   -0.54745924 1.613846
## time2                         -0.04875347 2.112897
## PSQI_group2                   -0.18089340 2.060365
## conditionMF:time2             -0.82154210 2.181388
## conditionMF:PSQI_group2       -1.56567492 1.483228
## time2:PSQI_group2             -1.34952362 1.726486
## conditionMF:time2:PSQI_group2 -1.91078386 2.423147
ordinalmodel_PSQI_KSS_3 <- clm(value ~ condition*PSQI_group, data = Data_PSQIgroups_KSS_Pivot)
```

summaryksspsqi2 <- drop1(ordinalmodel_PSQI_KSS_3, test = "Chisq")

summary(ordinalmodel_PSQI_KSS_3)

```
## formula: value ~ condition * PSQI_group
## data:    Data_PSQIgroups_KSS_Pivot
##
##  link  threshold nobs logLik  AIC    niter max.grad cond.H
##  logit flexible  168  -306.94 635.88 6(0)  5.24e-08 2.2e+02
##
## Coefficients:
##                         Estimate Std. Error z value Pr(>|z|)
## conditionMF              0.76864    0.38370   2.003   0.0452 *
## PSQI_group2              0.90935    0.39186   2.321   0.0203 *
## conditionMF:PSQI_group2 -0.04621    0.54802  -0.084   0.9328
## ---
## Signif. codes:  0 '***' 0.001 '**' 0.01 '*' 0.05 '.' 0.1 ' ' 1
##
## Threshold coefficients:
##     Estimate Std. Error z value
## 1|2 -4.45909    1.02125  -4.366
## 2|3 -1.99690    0.36740  -5.435
## 3|4 -0.09914    0.27655  -0.358
## 4|5  0.95887    0.29161   3.288
## 5|6  1.49908    0.30569   4.904
## 6|7  2.52732    0.34184   7.393
## 7|8  3.71806    0.42978   8.651
## 8|9  5.40270    0.76629   7.050
print(summaryksspsqi2)
## Single term deletions
##
## Model:
## value ~ condition * PSQI_group
##                      Df    AIC       LRT Pr(>Chi)
## <none>                  635.88
## condition:PSQI_group  1 633.89 0.0071089   0.9328
ordinalmodel_PSQI_KSS_4 <- clm(value ~ time*PSQI_group, data = Data_PSQIgroups_KSS_Pivot)
```

summaryksspsqi3 <- drop1(ordinalmodel_PSQI_KSS_4, test = "Chisq")

summary(ordinalmodel_PSQI_KSS_4)

```
## formula: value ~ time * PSQI_group
## data:    Data_PSQIgroups_KSS_Pivot
##
##  link  threshold nobs logLik  AIC    niter max.grad cond.H
##  logit flexible  168  -298.08 618.17 6(0)  5.92e-08 1.9e+02
##
## Coefficients:
##                   Estimate Std. Error z value Pr(>|z|)
## time2               1.3059     0.3935   3.318 0.000905 ***
## PSQI_group2         0.8828     0.3926   2.249 0.024536 *
## time2:PSQI_group2   0.2232     0.5546   0.403 0.687298
## ---
## Signif. codes:  0 '***' 0.001 '**' 0.01 '*' 0.05 '.' 0.1 ' ' 1
##
## Threshold coefficients:
##     Estimate Std. Error z value
## 1|2  -4.3098     1.0193  -4.228
## 2|3  -1.8250     0.3648  -5.003
## 3|4   0.1357     0.2826   0.480
## 4|5   1.2655     0.3044   4.158
## 5|6   1.8495     0.3216   5.751
## 6|7   2.9497     0.3626   8.135
## 7|8   4.2028     0.4518   9.302
## 8|9   5.9200     0.7805   7.585
print(summaryksspsqi3)
## Single term deletions
##
## Model:
## value ~ time * PSQI_group
##                 Df    AIC     LRT Pr(>Chi)
## <none>             618.17
## time:PSQI_group  1 616.33 0.16208   0.6873
ordinalmodel_PSQI_KSS_5 <- clm(value ~ PSQI_group, data = Data_PSQIgroups_KSS_Pivot)
```

summaryksspsqi4 <- drop1(ordinalmodel_PSQI_KSS_5, test = "Chisq")

summary(ordinalmodel_PSQI_KSS_5)

```
## formula: value ~ PSQI_group
## data:    Data_PSQIgroups_KSS_Pivot
##
##  link  threshold nobs logLik  AIC    niter max.grad cond.H
##  logit flexible  168  -310.61 639.22 6(0)  5.02e-08 2.0e+02
##
## Coefficients:
##             Estimate Std. Error z value Pr(>|z|)
## PSQI_group2   0.8539     0.2795   3.055  0.00225 **
## ---
## Signif. codes:  0 '***' 0.001 '**' 0.01 '*' 0.05 '.' 0.1 ' ' 1
##
## Threshold coefficients:
##     Estimate Std. Error z value
## 1|2  -4.7820     1.0083  -4.743
## 2|3  -2.3249     0.3287  -7.074
## 3|4  -0.4639     0.2059  -2.254
## 4|5   0.5628     0.2098   2.683
## 5|6   1.0838     0.2222   4.878
## 6|7   2.0793     0.2639   7.880
## 7|8   3.2538     0.3692   8.813
## 8|9   4.9294     0.7336   6.720
print(summaryksspsqi4)
## Single term deletions
##
## Model:
## value ~ PSQI_group
##            Df    AIC    LRT Pr(>Chi)
## <none>        639.22
## PSQI_group  1 646.73 9.5138 0.002039 **
## ---
## Signif. codes:  0 '***' 0.001 '**' 0.01 '*' 0.05 '.' 0.1 ' ' 1
exp(coef(ordinalmodel_PSQI_KSS_3))
##                     1|2                     2|3                     3|4
##              0.01157288              0.13575489              0.90561832
##                     4|5                     5|6                     6|7
##              2.60874116              4.47755717             12.51989948
##                     7|8                     8|9             conditionMF
##             41.18458850            222.00458618              2.15682792
##             PSQI_group2 conditionMF:PSQI_group2
##              2.48270046              0.95484580
Data_PSQIcont_KSS_Pivot$value <- factor(Data_PSQIcont_KSS_Pivot$value, ordered = TRUE)
```

Data_PSQIcont_KSS_Pivot$condition <- as.factor(Data_PSQIcont_KSS_Pivot$condition)

Data_PSQIcont_KSS_Pivot$time <- as.factor(Data_PSQIcont_KSS_Pivot$time)

ordinalmodel_PSQIcont_KSS <- clm(value ~ condition*time*PSQI, data = Data_PSQIcont_KSS_Pivot)

summary(ordinalmodel_PSQIcont_KSS)

```
## formula: value ~ condition * time * PSQI
## data:    Data_PSQIcont_KSS_Pivot
##
##  link  threshold nobs logLik  AIC     niter max.grad cond.H
##  logit flexible  408  -722.10 1474.20 7(0)  5.41e-13 9.7e+03
##
## Coefficients:
##                         Estimate Std. Error z value Pr(>|z|)
## conditionMF             0.558793   0.555166   1.007    0.314
## time2                   0.827734   0.556184   1.488    0.137
## PSQI                    0.133694   0.088558   1.510    0.131
## conditionMF:time2       0.396222   0.781558   0.507    0.612
## conditionMF:PSQI       -0.005434   0.120534  -0.045    0.964
## time2:PSQI              0.030482   0.121453   0.251    0.802
## conditionMF:time2:PSQI  0.064597   0.171101   0.378    0.706
##
## Threshold coefficients:
##     Estimate Std. Error z value
## 1|2  -4.1592     0.7985  -5.209
## 2|3  -1.4660     0.4214  -3.479
## 3|4   0.3982     0.4042   0.985
## 4|5   1.6008     0.4152   3.855
## 5|6   2.1763     0.4211   5.168
## 6|7   3.2707     0.4350   7.518
## 7|8   4.4444     0.4653   9.551
## 8|9   6.7925     0.7195   9.441
```

[Converted to Word document with WordToHTML.net](https://wordtohtml.net/?utm_source=wth_free_word_conversion_link&utm_medium=external)
